# Population genomics reveals deep diversification in Malayan pangolins

**DOI:** 10.1093/molbev/msag016

**Published:** 2026-01-16

**Authors:** Bo Li, Haimeng Li, Minhui Shi, Qing Wang, Huixin Li, Ce Guo, Jingyang Hu, Boyang Liu, Yinping Tian, Shanlin Liu, Kristen Finch, Shiqing Wang, Shangchen Yang, Liangyu Cui, Jun Li, Xilong Zhao, Zhangwen Deng, Yue Ma, Hyeon Jeong Kim, Samuel K Wasser, Kai Wang, Haorong Lu, Jin Chen, Huabing Guo, Yan Yao, Hui Xie, Yiyi Wang, Jiale Fan, Yu Lin, Yinmeng Hou, Yuan Fu, Chuan Jiang, Jinyao Lu, Siyuan Li, Zhaowen Qiu, Wei Zhang, Suying Bai, Lei Han, Zhen Wang, Chen Wang, Jiahao Li, Yuze Jiang, Shasha Liu, Jiayi Wang, Li Yu, Qiye Li, Li Li, Yan Hua, Tianming Lan, Yanchun Xu

**Affiliations:** College of Wildlife and Protected Area, Northeast Forestry University, Harbin 150040, China; College of Wildlife and Protected Area, Northeast Forestry University, Harbin 150040, China; Heilongjiang Key Laboratory of Complex Traits and Protein Machines in Organisms, Harbin 150040, China; BGI Life Science Joint Research Center, Northeast Forestry University, Harbin 150040, China; BGI Research, Wuhan 430074, China; BGI Life Science Joint Research Center, Northeast Forestry University, Harbin 150040, China; School of Medicine, Nanjing University of Chinese Medicine, Nanjing 210023, China; Chinese Academy of Forestry, Beijing 100091, China; College of Wildlife and Protected Area, Northeast Forestry University, Harbin 150040, China; Guangdong Provincial Key Laboratory of Silviculture, Protection and Utilization, Guangdong Academy of Forestry, Guangzhou 510520, China; State Key Laboratory for Conservation and Utilization of Bio-Resource in Yunnan, School of Life Sciences, Yunnan University, Kunming 650500, China; College of Wildlife and Protected Area, Northeast Forestry University, Harbin 150040, China; College of Wildlife and Protected Area, Northeast Forestry University, Harbin 150040, China; Key Laboratory of Zoological Systematics and Evolution, Institute of Zoology, Chinese Academy of Sciences, Beijing 100101, China; Center for Environmental Forensic Science, University of Washington, Seattle 98195, USA; BGI Research, Wuhan 430074, China; College of Life Sciences, Zhejiang University, Hangzhou 310058, China; College of Wildlife and Protected Area, Northeast Forestry University, Harbin 150040, China; College of Wildlife and Protected Area, Northeast Forestry University, Harbin 150040, China; Guangdong Provincial Key Laboratory of Silviculture, Protection and Utilization, Guangdong Academy of Forestry, Guangzhou 510520, China; College of Wildlife and Protected Area, Northeast Forestry University, Harbin 150040, China; Guangxi Zhuang Autonomous Region Forest Inventory and Planning Institute, Nanning 530011, China; College of Wildlife and Protected Area, Northeast Forestry University, Harbin 150040, China; Center for Environmental Forensic Science, University of Washington, Seattle 98195, USA; Center for Environmental Forensic Science, University of Washington, Seattle 98195, USA; Guangdong Provincial Key Laboratory of Silviculture, Protection and Utilization, Guangdong Academy of Forestry, Guangzhou 510520, China; BGI Research, Wuhan 430074, China; College of Wildlife and Protected Area, Northeast Forestry University, Harbin 150040, China; Forest Inventory and Planning Institute of Jilin Province, Changchun 130022, China; Hunan Biodiversity Conservation Center, Changsha 410000, China; Movement System Injury and Repair Research Center, Xiangya Hospital, Central South University, Changsha 410008, China; Movement System Injury and Repair Research Center, Xiangya Hospital, Central South University, Changsha 410008, China; College of Wildlife and Protected Area, Northeast Forestry University, Harbin 150040, China; College of Wildlife and Protected Area, Northeast Forestry University, Harbin 150040, China; College of Life Sciences, Henan Normal University, Xinxiang 453007, China; College of Wildlife and Protected Area, Northeast Forestry University, Harbin 150040, China; College of Wildlife and Protected Area, Northeast Forestry University, Harbin 150040, China; Hunan Biodiversity Conservation Center, Changsha 410000, China; College of Veterinary Medicine, Huazhong Agriculture University, Wuhan 430070, China; College of Wildlife and Protected Area, Northeast Forestry University, Harbin 150040, China; College of Wildlife and Protected Area, Northeast Forestry University, Harbin 150040, China; College of Wildlife and Protected Area, Northeast Forestry University, Harbin 150040, China; College of Wildlife and Protected Area, Northeast Forestry University, Harbin 150040, China; College of Wildlife and Protected Area, Northeast Forestry University, Harbin 150040, China; College of Wildlife and Protected Area, Northeast Forestry University, Harbin 150040, China; College of Wildlife and Protected Area, Northeast Forestry University, Harbin 150040, China; Hunan Botanical Garden, Changsha 410004, China; College of Veterinary Medicine, South China Agricultural University, Guangzhou 510642, China; College of Veterinary Medicine, South China Agricultural University, Guangzhou 510642, China; State Key Laboratory for Conservation and Utilization of Bio-Resource in Yunnan, School of Life Sciences, Yunnan University, Kunming 650500, China; BGI Research, Wuhan 430074, China; Hunan Biodiversity Conservation Center, Changsha 410000, China; Guangdong Provincial Key Laboratory of Silviculture, Protection and Utilization, Guangdong Academy of Forestry, Guangzhou 510520, China; College of Wildlife and Protected Area, Northeast Forestry University, Harbin 150040, China; Heilongjiang Key Laboratory of Complex Traits and Protein Machines in Organisms, Harbin 150040, China; BGI Life Science Joint Research Center, Northeast Forestry University, Harbin 150040, China; College of Wildlife and Protected Area, Northeast Forestry University, Harbin 150040, China; BGI Life Science Joint Research Center, Northeast Forestry University, Harbin 150040, China

**Keywords:** Malayan pangolin, population genomic, divergence, genetic diversity, conservation

## Abstract

Archipelagos and oceanic islands have remarkably high levels of endemism, which is associated with rapid speciation. The Malayan pangolin (*Manis javanica*), one of critically endangered Asia pangolin species, occurs in southern Yunnan, China, and on oceanic islands via the Malay peninsula. The question of whether the distribution of Malayan pangolins between the mainland and nearby marine islands has led to deep population differentiation is not well addressed. In-depth investigation of population structure and genetic consequences is of vital importance for protection and conservation of Malayan pangolins. Here we carried out a large-scale population genomic analysis for Malayan pangolins, which revealed three highly distinct genetic populations. The largest population was found to be distributed over a wide area extending from mainland China to almost the whole of South East Asia. The other two smaller populations reported in this study were inferred from Borneo. In addition, based on multiple lines of genomic and skull morphological evidences, we confirmed the existence of a fifth Asian pangolin species (*M. mysteria*). Genetic diversity and genome-wide inbreeding were at moderate levels, indicating that anthropogenic factors did not significantly weaken the basis of genetic sustainability for Malayan pangolins. However, Malayan pangolins from northeastern Borneo exhibited low genetic diversity, high levels of inbreeding and mutational load, thereby necessitating attention to their protection.

## Introduction

Compared with mainland regions, islands tend to have rich biodiversity, often with a high percentage of endemism related to rapid adaptive divergence (Stuart et al. 2012). For species that are widely distributed across mainland areas and islands, greater differentiation may be observed in its island populations compared with its mainland ones. The Malayan pangolin (*Manis javanica*, Desmarest 1822) is one of critically endangered pangolin species (Challender et al. 2019) and being listed in the CITES appendix I (Heinrich et al. 2016). The species is a typical mammal that exists on both the mainland of China (Jiang et al. 2017) and Southeast Asia (Challender et al. 2019) and on oceanic islands in the Pacific, namely those in the vicinity of the Malay Peninsula. The range of the Malayan pangolin is characterized by high ecological heterogeneity (Nash et al. 2018) and lies within a geographic region that is recognized as a biodiversity hotspot, harboring some of the highest levels of biodiversity in the world (Myers et al. 2000). Given geographic isolation and habitat fragmentation for the Malayan pangolin, deep differentiation would be expected within the species. The geophylogeny of Malayan pangolin populations requires special attention for conservation, owing to the unique evolutionary significance of local populations. The genetic distinctions between existing populations can be difficult to properly identify, however, placing these populations at risk of being overlooked. Additionally, Malayan pangolin numbers have declined toward the brink of collapse (Liu and Weng 2014), due to extensive habitat loss and illegal poaching (Xing et al. 2020). Therefore, conservation is urgently needed, with focus on poaching and illegal trade prevention, habitat and population restoration, and rescued animal rehabilitation (Wright and Jimerson 2020).The effectiveness of conservation actions is aided significantly by understanding of the genetic background of the threatened species, particularly in terms of its population structures (Buckland et al. 2014).

Genetic differentiation in Malayan pangolins has been explored previously. Philippine pangolin (*M. culionensis*) historically being regarded as a subspecies of the Malayan pangolin, was later defined as an independent species based on significant morphological and genetic differences resulting from long-term geographic separation (Gaubert and Antunes 2005; Zhang et al. 2015; Gaubert et al. 2018) observed a unique monophyletic clade in the group of Philippine pangolin and Malayan pangolin, and proposed it could be the fifth Asian pangolin species diverging from Malayan pangolins 6.95 million years ago (MYA) (Hu et al. 2020a). This new pangolin species was recently named as *M. mysteria*, which was confirmed by genome-wide evidence and limited morphological evidence (scale shape) (Gu et al. 2023). Concurrently, genetic analysis has suggested that Malayan pangolins could be divided into several clades and subclades (Nash et al. 2018; Hu et al. 2020b; Sitam et al. 2023) . For instance, genomic multiplex analysis (Hu et al. 2020b) revealed that the species is divided into two genetically distinct populations: the continentally distributed MJA and island-dwelling MJB. These studies collectively suggest that genetic divergence among Malayan pangolin populations is complex and might not have been inadequately addressed.

In this study, we utilized the largest genomic dataset currently available to conduct a systematic investigation into the fine-scale genetic structure of Malayan pangolin populations. These analyses resulted in the identification of two new Malayan pangolin lineages, while also providing additional evidences (firstly including morphometric features of skulls) in support of the Asian mysterious pangolin newly proposed by Gu et al. (2023). Furthermore, we assessed potential genome-wide risks across all Malayan pangolin populations, thereby offering novel insights for global conservation of this species.

## Results

### Species confirmation and genome sequencing of our samples

We performed whole-genome resequencing of 594 confiscated pangolins (Figure S1). Initially, we constructed a mitogenome phylogenetic tree to conduct a preliminary species confirmation (Figure S2, *N* = 687). Considering that the morphological characteristics were not complete some of our samples, we further confirmed the species via BLAST searches against the NCBI database. Ultimately, we identified 582 Malayan pangolins and 12 individuals belonging to other pangolins species, including one giant pangolin (*Smutsia gigantea*), three African tree pangolins (*Phataginus tricuspis*), four Indian pangolins (*M. crassicaudata*), two Chinese pangolins (*Manis pentadactyla*), and two suspected Asian mysterious pangolins (*M. mysteria*, MSP) (Additional File 1: Figures S1, S2 and Tables S1, S2).

Thirty-nine individuals with sequencing coverage below 80% and 20 individuals with close relationship were discarded from the later whole-genome analyses (Additional File 1: Tables S1 and S3). The clean re-sequencing data of the remaining 525 pangolins showed an average sequencing depth of 18.72 ± 3.82-fold. In addition, we did not detect the deamination-induced C to T changes at either end of the reads for all samples. The level of suspected human contamination estimated was no more than 0.7%, suggesting the credibility and reliability of the sequencing data were acceptable (Additional File 1: Figure S3 and Table S4).

### Additional evidences to support the Asian mysterious pangolin

**Figure 1:**
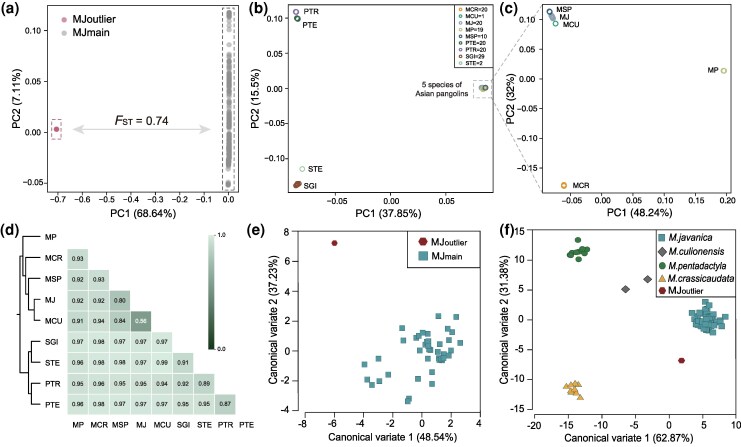
New evidence to support the Asian mysterious pangolins. (a) PCA of 598 pangolin genomes with the *F*_ST_ between MJ_outlier_ (MJ-DCW-89 and MJ-DCW-116) and the MJ_main_ (*N* = 596). (b) PCA results of 141 pangolins were used to identify nine pangolin species. Further analysis of five Asian pangolin species is shown in Figure S5. (c) Genetic clustering of the five Asian pangolin species based on PCA. (d) *F*_ST_ between each pair of nine pangolin species with their evolutionary relationship (Maximum-Likelihood phylogenetic tree). MCR = *Manis crassicaudata* (*N* = 20), MCU = *M. culionensis* (*N* = 1), MJ = *M. javanica* (*N* = 20), MP = *M. pentadactyla* (*N* = 19), MSP (*N* = 10), *M. mysteria* (*N* = 7), MJ_outlier_ (*N* = 2), and PA (*N* = 1). PTE = *Phataginus tetradactyla* (*N* = 20), PTR = *P. tricuspis* (*N* = 20), SGI = *Smutsia gigantea* (*N* = 29), and STE = *S. temminckii* (*N* = 2). (e) Canonical variate scatter plot showing morphological distinctness between skulls of MJ_outlier_ and MJ_main_. (f) Canonical variate scatter plot showing morphological distinctness of skulls of MJ_outlier_ (MJ-DCW-116) and four recognized Asian pangolin species, including MJ_main_, along the first two canonical variate axes.

To further verify the validity of Asian mysterious pangolins, we conducted a fine-scale genetic structure analysis across the aforementioned nine pangolin species. We first performed variants calling by integrating 596 Malayan pangolins (523 from this study and 73 from published data) (Additional File 1: Table S1 and S5) and two suspected Asian mysterious pangolins (MJ-DCW-89 and MJ-DCW-116). From the genome data of 598 pangolins, we identified a total of 96,217,079 SNPs. The principal component analysis (PCA) revealed that MJ-DCW-89 and MJ-DCW-116 formed a distinct cluster (hereafter referred to as MJ_outlier_), which was highly differentiated from the main Malayan pangolin population (hereafter referred to as MJ_main_) (Fig. 1a). The differentiation was further supported by maximum likelihood (ML) phylogenetic tree which was constructed using genome-wide autosomal SNPs showing the two individuals residing in a distinct clade (Additional File 1: Figure S4). In addition, *F*_ST_ value between MJ_outlier_ and MJ_main_ reached 0.74 (Additional File 1: Table S6).

We integrated 141 pangolins including MJ-DCW-89, MJ-DCW-116 and 139 other pangolins representing all pangolin species (Additional File 1: Table S7) to investigate the phylogenetic position of MJ_outlier_ within *Manis*. The PCA using 219,725,558 SNPs identified across the 141 pangolins showed the MSP including MJ-DCW-89, MJ-DCW-116 in this study, Palawan pangolin (PA) identified by Cao et al. (2021), and the Asian mysterious pangolin reported in Gu et al. (2023) formed a well-supported clade (Fig. 1b, c and Figure S5). This clustering pattern was also corroborated by the ML tree and admixture analysis (Additional File 1: Figures S6, S7). Additionally, the pairwise *F*_ST_ values between MSP and each of other pangolin species ranged from 0.80 to 0.98 (Fig. 1d). The interspecific phylogenetic relationships among all currently recognized pangolin species were consistent with those reported in Gu et al. (2023) (Figure S6). These findings indicated that MJ_outlier_, together with the PA in Cao et al. (2021), belong to the recently identified MSP.

To search new morphological evidence for Asian mysterious pangolins, we collected 42 skulls from the sequenced pangolins, including the MJ-DCW-116 from the MJ_outlier_ and 41 individuals from the MJ_main_ (Additional File 1: Table S8). Analysis showed that the skull of MJ-DCW-116 exhibited greater and stronger pterygoid hamulus compared with the 41 skulls from the MJ_main_ (Additional File 1: Figure S8a). Moreover, 13 out of 75 anatomical landmarks on this skull deviated significantly from the average shape of typical Malayan pangolins (Additional File 1: Figures S8b, c and S9 to S11). Canonical variable analysis (CVA) also confirmed the morphological distinctness of MJ-DCW-116 from both MJ_main_ individuals and the four recognized Asian pangolin species (Fig. 1e, f and Additional File 1: Tables S9–S12). Collectively, morphological differentiation of MJ_outlier_ strongly supported the existence of MSP.

### Deep differentiation among Malayan pangolins

#### Genetic structure

**Figure 2:**
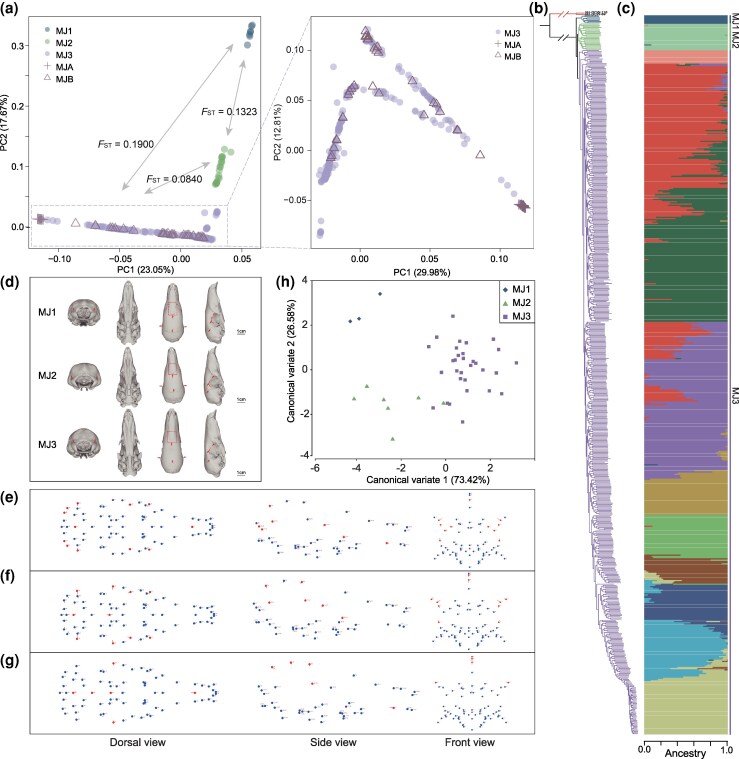
Genetic structure and differentiation of Malayan pangolins. (a) PCA of all 596 Malayan pangolin genomes from the clade MJ_main_ (left), and PCA of 567 Malayan pangolin genomes from the MJ3 (right). In Hu et al. (2020b), the 73 Malayan pangolin samples were divided into MJA and MJB, but were connected as a single clade the MJ3 by additional sample in the present study and classified into the MJ3 population, shown in the figure with hollow triangles and crosses. (b) Maximum-likelihood (ML) tree based on the genome-wide SNPs with MJ_outlier_ (MJ-DCW-89 and MJ-DCW-116) serving as an outgroup. (c) Admixture analysis of all 596 pangolins (*K* = 10). (d) The reconstructed 3D morphology of the skulls of the MJ1, MJ2, and MJ3 populations. The red arrows point to the regions that differed between populations; (e–g) The dorsal, side, and front view of landmark positional variation between the MJ1 and MJ2 populations (e), MJ1 and MJ3 populations (f), and MJ2 and MJ3 populations (g), respectively. Red dots indicate main positional changes. (h) Scatter plot showing the variation in skull shape along the canonical variate axes for the three Malayan pangolin populations.

Since MJ-DCW-89 and MJ-DCW-116 were members of MSP, they were excluded from Malayan pangolins in intraspecific population structure analysis. Therefore, a total of 596 Malayan pangolin genomes containing 1,084,855 SNPs (thinned from an initial set of 82,890,778 SNPs) were used (Additional File 1: Figure S1). PCA revealed that these pangolins clustered into three distinct genetic groups, designated as MJ1 (*N* = 7), MJ2 (*N* = 22), and MJ3 (*N* = 567) (Fig. 2a). This finding was further validated by the ML tree and admixture analysis (Fig. 2b, c and Additional File 1: Figure S12). The ML tree also indicated that the MJ1 was likely the most ancestral, with MJ2 and MJ3 representing more recent and derived lineages. The clade MJ3 was more complex than MJ1 and MJ2. It contained not only 494 pangolins of this study, but all 73 specimens that had been classified into two populations (MJA and MJB) in Hu et al. (2020b). We show MJA and MJB were fully connected by addition of our samples, forming a single integrated large group (Fig. 2a). Furthermore, considerable divergence was observed between MJ1 and MJ2 (*F*_ST_ = 0.13), and between MJ1 and MJ3 (*F*_ST_ = 0.19, Fig. 2a and Additional File 1: Table S6), indicating deep diversification within Malayan pangolins. To mitigate the bias introduced by uneven sample size across populations, we randomly selected 10 individuals from both MJ2 and MJ3 for replicate analyses. The results confirmed the population structure inferred from the full dataset (Additional File 1: Figure S13). Notably, the mitochondrial phylogenetic tree clustered a putative *M. javanica* sample P442988 with MSP (Additional File 1: Table S1<CE: Please check the table 1 is treated as supplementary table S1 here and elsewhere. Please check and take care.>, Figure S2 and S4), while its nuclear genome was entirely affiliated to Malayan pangolin (Fig. 2b), suggesting nuclear-mitochondrial disconcordance of this sample.

#### Morphometric variation of skulls

Three aforementioned Malayan pangolin clades exhibited significant differentiation in skull morphology (Fig. 2d–g). CVA clearly distinguished the three clades in the scatterplot (Fig. 2h), with the first two canonical variates explaining 100% of total variation (CV1 = 73.42%, CV2 = 26.58%). Furthermore, distinct morphological characteristics were identified among the three populations (Additional File 1: Tables S13 to S15). Specifically, the distance from the posterior most point on the supraoccipital skull roof (landmark 64) to the rostral side was obviously greater in MJ2 members than that in MJ1 and MJ3 (dorsal and lateral views). In contrast, the distance from the posterior point of the premaxilla (landmark 3) to the rostral side was much shorter in MJ2 compared with MJ1 and MJ3. Additionally, landmarks 60, 61, and 73 were positioned closer to the rostral side in MJ3 relative to MJ1 and MJ2. Moreover, prominent differences were also observed that the intersection of the interparietal and interfrontal sutures (landmark 60) was positioned higher in MJ1 than in MJ2 and MJ3; The distance between the frontal-parietal-squamosal intersections on both sides (landmarks 61 and 73) was greater in MJ1 than in MJ2 and MJ3; and the distance between the intersections of the squamosal, parietal, and exoccipital bones on both sides (landmarks 63 and 74) was greater in MJ1 skulls compared to the other two clades.

#### Inference of geographic origin of the three populations

**Figure 3:**
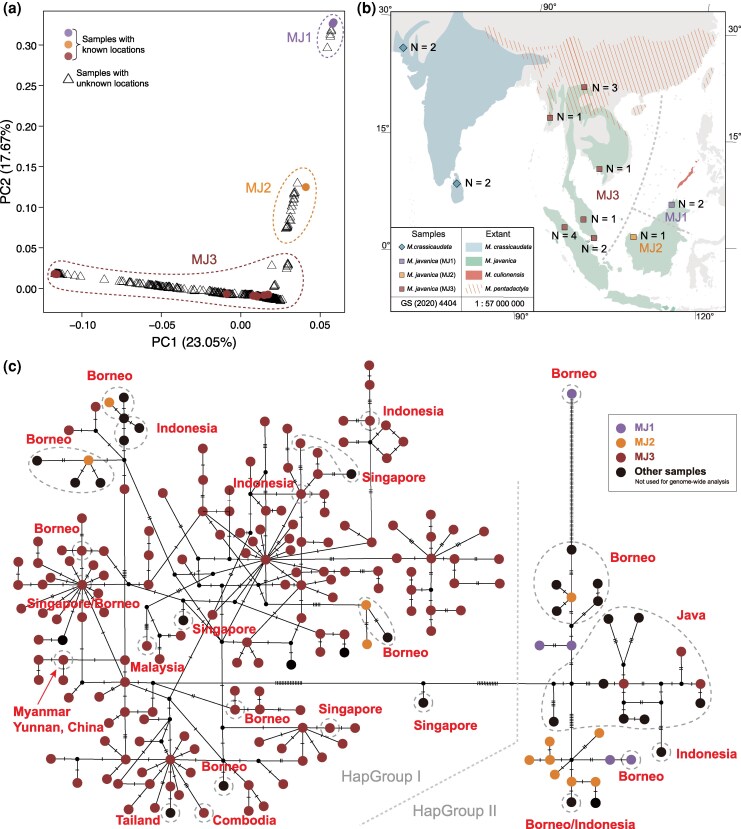
Geographic origin inference of the three Malayan pangolin populations. (a) PCA plot of 596 Malayan pangolin individuals-based genome-wide SNPs. Samples with detailed information on known location were marked as solid colored circles. (b) The geographic range of the typical Malayan pangolin, showing the samples with known sampling sites. (c) Network of 202 mitochondrial haplotypes of Malayan pangolins (*N* = 715). Haplotypes in gray dashed circles are the published haplotypes with known sampling sites. The three Malayan populations defined in this study are shown in different colors.

We sought to assign the geographic origin of the three identified populations by comparing them with 15 Malayan pangolins with known origin (Fig. 3, Table S1). PCA analysis of nuclear genomes showed that two Malaysian samples from northeastern Borneo clustered within the MJ1, while one Malaysian sample from northwestern Borneo clustered within the MJ2 (Fig. 3a, b). This suggests that the typical Malayan pangolins on Borneo may have diverged into two local populations, corresponding to MJ1 in the northeast and MJ2 in the northwest (Fig. 3b). The rest 12 known-origin samples were assigned to the MJ3, including three from China, one from Myanmar, one from Cambodia, four from Singapore, one from Malaysia, and two from Indonesia (Fig. 3b). This indicates that MJ3 is likely a large population encompassing continental, peninsular, and insular (Sumatran) local populations.

We also reconstructed a haplotype network with concatenated *COX1* and *Cytb* sequences were successfully assembled (*N* = 715), including 582 individuals in this study (10 of which had known origins), 73 published individuals (5 with known origins), and 60 mitochondrial sequences from databases (59 with known origins) (Fig. 3c, Additional File 1: Figure S14 and Tables S16, S17). We show haplotypes generally clustered into two main groups: HapGroup I and HapGroup II. A focal examination revealed that the haplotypes from MJ1 and MJ2 originated from Borneo, while those from the MJ3 were widely distributed across the mainland and nearly all East Asian islands (Fig. 3c). This inference is consistent with findings from nuclear genome (Fig. 2a to c).

### Demography of Malayan pangolin populations

**Figure 4:**
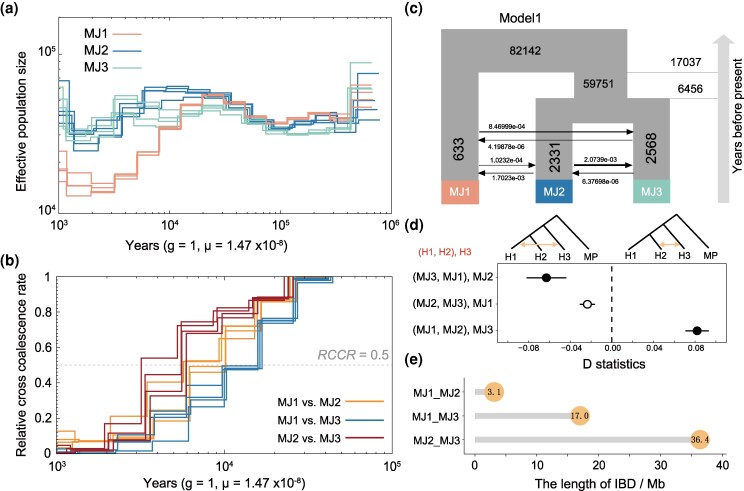
Population dynamics and gene flow among three Malayan pangolin populations. (a) The changes in the early effective population size of the three populations over time inferred by MSMC2. (b) Inferred divergence time among three populations. (c) Most likely demographic model inferred from fastsimcoal2. (d) D-statistics analysis with the Chinese pangolin (MP) as outgroup. H1, H2, and H3 represent population of MJ1, MJ2, and MJ3, respectively. Each solid circle represents the significant gene flow with a *Z*-score larger than 3. The dashed vertical line denotes *D* = 0. (e) The IBD segments shared between each pair of the three populations.

The three Malayan pangolin populations exhibited similar demographic trajectories consisting of four phases (Fig. 4a): a sharp decline from 0.5 to 0.4 million years ago (Mya), followed by a trough period with slight fluctuations until approximately 70 thousand years ago (Kya), then a gradual expansion peaking at around 20 Kya, and then the second decline took place until roughly 2 Kya. However, we observed that the MJ1 initiated its decline earlier in the second decline stage than the other two populations (beginning at 20 Kya) and experienced a more pronounced decline. This pattern contributed to the divergence of MJ1 from MJ2 and MJ3 around 10 to 20 Kya, while MJ2 and MJ3 separated approximately 6 Kya (Fig. 4b). We further inferred the demographic histories of the three populations by comparing different demographic model simulations, and the best model also supported the separation events inferred by the MSMC method (Fig. 4c and Figure S15).

We then investigated genomic signatures of post-divergence gene flow between each pair of the three populations using D-statistics and identity-by-descent (IBD) analysis, with the Chinese pangolin (MP) as the outgroup (Fig. 4d, e). The D-statistics identify shared derived alleles between two populations, which could detect more historical gene flow, whereas the large IBD fragments shared by two population could represent the more recent gene flow between populations (Bai et al. 2018). In this study, signs of both historical and potentially recent gene flow were observed between the MJ2 and MJ3 populations, whereas gene flow between MJ1 and MJ2 or MJ1 and MJ3 appeared to be barely detectable. This suggests that MJ1 is a relatively isolated population. IBD patterns and the demographic model inferred by fastsimocal2 further support the most extensive gene flow between MJ2 and MJ3 (Fig. 4c and e). These shared segments are randomly distributed across the genome, with no apparent clustering in specific regions (Figure S16).

### Genetic diversity, inbreeding, and mutational load

**Figure 5:**
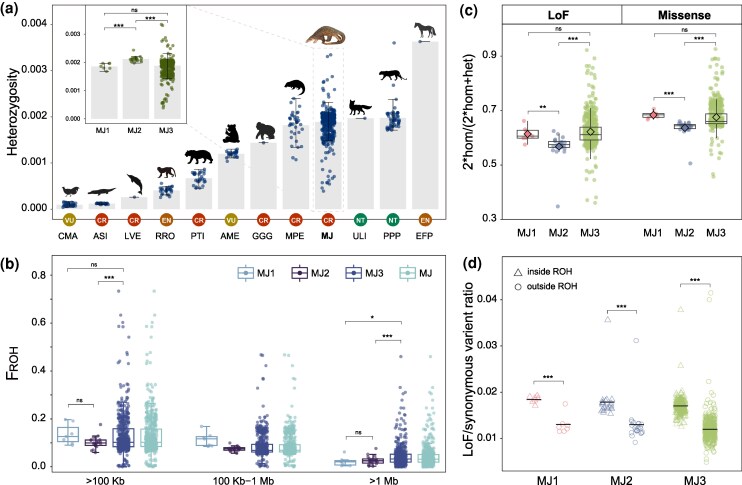
Genetic diversity, ROH, and mutational loads in Malayan pangolin populations. (a) Comparison of genome-wide heterozygosity of Malayan pangolin with that of other endangered species. Colored dots represent individuals and whiskers represent the 95% confidence interval of the genome-wide heterozygosity in each species. *X*-axis abbreviations are as follows: CMA: brown eared pheasant (*Crossoptilon mantchuricum*); ASI: Chinese alligator (*Alligator sinensis*); LVE: baiji (*Lipotes vexillifer*); RRO: golden snub-nosed monkey (*Rhinopithecus roxellana*); PTI: South China tiger (*Panthera tigris amoyensis*) and Siberian tiger (*P. t. altaica*); AME: giant panda (*Ailuropoda melanoleuca*); GGG: western lowland gorilla (*Gorilla gorilla gorilla*); MPE: Chinese pangolin (*M. pentadactyla*); MJ: Malayan pangolin (*Manis javanica*); ULI: island fox (*Urocyon littoralis*); PPP: African leopard (*Panthera pardus pardus*); and EFP: Przewalski's horse (*Equus ferus przewalskii*). (b) F_ROH_ with different length categories (ROH > 100 kb, 100 kb < ROH < 1 Mb, and ROH > 1 Mb) in the genomes of Malayan pangolins. MJ represents the whole population and MJ1, MJ2 and MJ3 represent three populations. (c) Genetic load in Malayan pangolin populations. The proportion of homozygous mutations was calculated with the formula: 2× homozygous sites/(2× homozygous sites + heterozygous site). (d) Comparison of the frequencies of LoF mutations derived from inside and outside the ROH region. The number of LoF mutations was normalized by the number of synonymous mutations in the same region to demonstrate the genetic purging effect in the Malayan pangolin populations.

We estimated genetic diversity of the Malayan pangolin populations using nucleotide diversity (*π*) and average heterozygosity (*He*). The overall *π* and *He* values for Malayan pangolins were 0.00217 ± 0.00118 and 0.00189 ± 0.00031, respectively (Additional File 1: Figure S17). These values were comparable to that of the critically endangered Chinese pangolin (*M. pentadactyla*, CR) and higher than those of other endangered mammalian species, including the giant panda (*Ailuropoda melanoleuca*, VN), the golden snub-nosed monkey (*Rhinopithecus roxellana*, EN), and the western lowland gorilla (*Gorilla gorilla gorilla*, CR) (Fig. 5a and Additional File 1: Table S18). Among the three populations, genetic diversity was the highest in MJ2 (*π:* 0.00231 ± 0.00134*; He:* 0.00212 ± 0.00009), followed by MJ3 (*π:* 0.00214 ± 0.00117*; He:* 0.00188 ± 0.00031) and MJ1 (*π:* 0.00205 ± 0.00130*; He:* 0.00186 ± 0.00011) in sequence.

Inbreeding level was assessed across different populations by screening genome-wide runs of homozygosity (ROHs) (Fig. 5b, Tables S19, S20, Figure S18). Overall, the inbreeding level (F_ROH_) of Malayan pangolins was 13.16 ± 0.38%, with an average ROH length of 332.48 ± 97.65 kb; and ROH fragments were predominantly short (<1 Mb, F_ROH (100kb-1Mb)_ = 8.73 ± 0.22%). Inferred from the number of generations since inbreeding events, the proportion of ROH derived from the most recent ancestors (within the last 26 generations, corresponding to ROH > 1 Mb) was significantly lower across all populations. Further comparison revealed that inbreeding level was negatively correlated with genome-wide heterozygosity among the three populations, lowest in MJ2 (F_ROH_ = 10.16 ± 0.52%), and similarly higher in MJ1 (F_ROH_ = 13.63 ± 1.62%) and MJ3 (F_ROH_ = 13.29 ± 0.40%) with slightly differed distribution patterns of ROH. MJ1 had the highest proportion of short ROH fragments (ranging from 100 Kb to 1 Mb, F_ROH (100kb-1Mb)_ = 11.44 ± 1.13%) among the three populations, indicating early inbreeding events, whereas MJ3 had the highest proportion of long ROH fragments (> 1 Mb, F_ROH > 1Mb_ = 4.55 ± 0.21%), suggesting that this population is experiencing more severe recent inbreeding.

We further screened mutational load by quantifying loss-of-function (LoFs) mutations and missense mutations across individual genomes (Fig. 5c, Table S21). Here, we focused on derived mutational load to mitigate calculation bias introduced by the reference genome. In terms of quantity, the total number of LoF and missense mutations were comparable in the three populations. The homozygous ratio was the lowest in MJ2, both for LoF (56.78 ± 5.28%) or missense mutations (63.65 ± 3.08%). This aligns with its highest genomic heterozygosity. For the homozygous ratio of strong deleterious LoF mutations, MJ3 (62.22 ± 5.98%) was slightly higher than MJ1 (61.39 ± 2.81%). In contrast, MJ1 exhibited a marginally higher homozygous ratio (68.37 ± 1.28%) for missense mutations. We performed GO term enrichment analysis on genes harboring shared strongly deleterious (LoF) mutations across the three populations (Figure S19). The results indicated that LoF mutations were randomly distributed throughout the genome, with no clustering of genes related to fitness (Figure S18). Notably, the density of LoF in ROH regions was significantly higher than that in non-ROH region (*P* < 0.005). Such a pattern could be interpreted as evidence for the active purging of deleterious mutations (Fig. 5d).

## Discussion

### New insights on the Asian mysterious pangolin

The fifth Asian pangolin, *M. mysteria* (MSP), was proposed based on genetic and limited morphological evidence (Hu et al. 2020b; Gu et al. 2023). Similarly, our multi-genomic analyses indicated that two pangolin individuals (MJ-DCW-89 and MJ-DCW-116) belong to this recently identified MSP. Skull morphology plays a pivotal role in mammalian taxonomy, serving as a cornerstone for species identification and phylogenetic classification (Ferreira-Cardoso et al. 2020). However, skull morphological characteristics was absent in the initial report of *M. mysteria* (Gu et al. 2023). In this study, we collected a skull specimen (MJ-DCW-116) of MSP, which exhibited greater and stronger pterygoid hamulus compared to that of Malayan pangolins (Figure S8a). Canonical variable analysis further confirmed the morphological distinctness of MJ-DCW-116 from both MJ_main_ individuals and the other four recognized Asian pangolin species (Fig. 1e, f and Additional File 1: Tables S11, S12). Our combined genomic and morphological evidence aligns with the geno-morph species concept (Hong 2020), supporting the validity of *M. mysteria*. Additionally, our data outlined the phylogenetic relationships within *Manis* as: *pentadactyla* + (*crassicaudata* + (*mysteria* + (*javanica* + *culionensis*))).

In addition to confirming the validity of *M. mysteria*, our analyses highlighted two noteworthy observations. First, a putative *M. javanica* sample P442988 exhibited nuclear-mitochondrial discordance: its mitogenome clustered with *M. mysteria*, while its nuclear genome aligned with *M. javanica* (Fig. 2b and Additional File 1: Figure S2). This pattern suggests historical introgression of *M. mysteria* genomic material into *M. javanica*—likely involving hybrids with maternal *mysteria* ancestry that repeatedly backcrossed with paternal *javanica* individuals (Mondol et al. 2015). Importantly, this sample was collected from northeastern Borneo (Table S1), leading us to hypothesize that *M. mysteria* may currently inhabit, or may have historically inhabited, northeastern Borneo or its adjacent regions.

The second point concerns sample PA, which was previously identified as *M. culionensis* by Cao et al. (2021). However, our analysis of nuclear genome phylogenetic relationships within *Manis* clustered it with the *M. mysteria* clade, indicating that it likely belongs to *M. mysteria* (Fig. 1c and Additional File 1: Figures S5, S6). In the ML tree of mitogenomes, it did not form a monophyletic clade with *M. culionensis*, instead, fell outside the clades containing *M. culionensis* and *M. javanica*. These results collectively suggest that PA may be a member of *M. mysteria* rather than *M. culionensis*. Further analyses incorporating additional genomes of *M. culionensis* would help clarify the phylogenetic relationship between these two pangolin species.

### Population differentiation in Malayan pangolins

Our large-scale population genomic analysis suggests that the Malayan pangolins comprise three genetically distinct groups: MJ1, MJ2, and MJ3. Notably, the continental and island populations proposed by Hu et al. (2020b) are connected in this study through the inclusion of samples from the MJ3. This suggests that MJ3 represents a continent-peninsula-island metapopulation corresponding to the MJ3 clade (Fig. 3a, b). Meanwhile, we detected extensive substructures in MJ3 (Fig. 2a–c), indicating the potential presence of small and isolated populations distributed across Southeast Asian islands. The identification of these substructures within MJ3 further clarifies the broader population framework and enhances the resolution of population structure analyses. On the other hand, Pleistocene climatic conditions may have played a crucial role in the population dynamics of Malayan pangolins (Hu et al. 2020b). Herein, MJ1, MJ2, and MJ3 underwent a second population decline stage—beginning around 20 Kya (Fig. 4a)—which was most likely driven by the Last Glacial Maximum (23–19 Kya, Hamilton et al. 2024). Another key factor influencing the demographic histories of Malayan pangolins was the Toba eruption in Sumatra, which occurred approximately 70 Kya (Martinez-Garcia et al. 2011). This time point nearly coincides with the end of the three population troughs (Fig. 4a).

We assumed that MJ1 and MJ2 inhabited in northeastern and northwestern Borneo, respectively, based on limited anchors of known-origin samples (Fig. 3). This was nearly similar to the finding that Sitam et al. (2023) identified two evolutionarily distinct mtDNA lineages in northern Borneo and western/southern Borneo, respectively. If our inference was true, it was indeed puzzling that two populations inhabiting the same island exhibit substantial genetic differentiation (*F*_ST_ = 0.1214) (Fig. 2a and Additional File 1: Table S6). The potential drivers of this divergence remain somewhat ambiguous, largely due to the absence of clear geographic boundaries on Borneo. Malayan pangolins have been recorded at altitudes up to 1700 m asl on Mount Kinabalu in Borneo (Payne and Francis 2007). However, the MaxEnt niche model indicates a declining presence of the species beyond 200 m asl (Panjang et al. 2024). As myrmecophagous species, pangolins depend solely on ants and termites for food. Yet, the diversity and numbers of these insects dwindle with increased altitude, potentially due to factors such as soil compaction, shallower humus layers, and temperature shifts (Pratiknyo et al. 2018). The scarcity of food sources, coupled with the pangolin's known sensitivity to cold, could restrict their expansion in higher-elevation habitats. Therefore, high mountain ranges on Borneo such as the Crocker Range and Iran Mountains in Sabah (northeastern Borneo), may act as elevation-based barriers that potentially contribute to population isolation. Further investigations involving expanded sampling across Borneo and enhanced ecological and behavioral observations would help clarify other drivers of this divergence.

We also identified significant gene flow between the MJ2 and the MJ3, suggesting that the distribution of MJ2 may extend beyond Borneo, with such additional habitats not being included in the present study. Furthermore, we observed substantial intraspecific diversification in Malayan pangolin. The three identified populations each form a monophyletic clade in the ML tree (Fig. 2b), and show moderate genomic differentiations with *F*_ST_ values ranging from 0.08 to 0.19. These findings support the notion that these populations represent significant evolutionary units. Notably, the noticeable genetic divergence of these populations—rendering their genetic pools irreplaceable—has critical conservation implications. Malayan pangolins in Southeast Asia are severely impacted by rampant trafficking and poaching (Zhang et al. 2015; Nash et al. 2018), with targeting any population risking permanent loss of ancient adaptive traits. Recognizing them as separate conservation units (CUs) is thus imperative to tailor strategies (e.g., anti-poaching, habitat protection) to each population's vulnerabilities, preserving their evolutionary potential amid threats.

### Genetic risk and conservation implications

Genetic diversity is an important factor for understanding the endangerment of species (Robinson et al. 2016) and informing conservation initiatives. We assessed genome-wide genetic diversity for three distinctive populations of the Malayan pangolin. The genome-wide *He* varied greatly in Malayan pangolins, but the majority was at a moderate level (Fig. 5a and Additional File 1: Table S18), suggesting a substantial basis for sustainability of this species. It is worth noting that the MJ3 population showed the most genetic variations among the three Malayan pangolin populations, but the average genetic diversity (*π* and *He*) of population MJ3 was significantly lower than that of population MJ2 and was at a similar level to that of population MJ1 (Fig. 5a and Additional File 1: Figure S17). This likely resulted from its distribution across a variety of habitats from the continent to nearby islands. Although this wide distribution could lead to the enrichment of total genetic variants, which could be also reflected from the scattered distribution of individual *He*, some small and isolated populations distributed at islands may be also included in the MJ3 population, which could reduce the population's average genetic diversity. Such a scenario, however, is very difficult to validate, unless clear sampling information can be traced; doing so could be significant for the future conservation of Malayan pangolins.

Among the three Malayan pangolin populations, the MJ1 presented the highest genome-wide inbreeding, low genetic diversity, and a high proportion of homozygous individuals with deleterious mutational loads. These characteristics align precisely with its position as the basal lineage in the ML phylogeny, which shows strong support for MJ1's early divergence. These genetic patterns reflect long-term isolation and small effective population size since its ancient split from the ancestor of MJ2/MJ3, leading to persistent genetic drift and accumulated deleterious mutations. Therefore, these factors together suggest that the MJ1 might potentially have the increased fitness reduction among all Malayan pangolin populations. Although we do not know the detailed geographical origin of the MJ1, we at least know that it is most likely from northeastern Borneo, an area that is worthy of special attention for pangolin conservation. Fortunately, we observed that the majority of ROH in all clades of typical Malayan pangolin was medium-sized (100 kb to 1 Mb), suggesting the pangolins have experienced high levels of ancient inbreeding instead of recent inbreeding over its population history. In particular, the ROH for medium-sized segments in MJ1 was 11.44 ± 1.13%, while the ROH for long segments was 2.19 ± 0.76%. The MJ3 population had the highest proportion of long segments (4.55 ± 0.21%), further supporting the previously proposed presence of small, isolated island populations within the MJ3 population. This may indicate that recent anthropogenic disturbances have exerted the most severe impacts on the population decline of Malayan pangolins, leaving open the possibility of a rapid population rebound if threatening anthropogenic factors, such as poaching, illegal trade, and habitat destruction, are eliminated.

## Methods

### Sample collection and ethics statement

Samples for a total of 594 pangolin individuals were collected and sequenced in this study, including 14 individuals from natural history museums in the United States (via the University of Washington Center for Environmental Forensic Science), and the remaining samples were from the Guangzhou Wildlife Rescue Center, Guangdong Province, China. Samples were collected under the supervision of Institutional Review Board of BGI and permission was obtained when necessary (BGI-IRB: E21056-T1).

### Morphometry analysis

To test morphometric variation of the sequenced pangolins, we scanned 42 skulls using Micro-CT (Scanco medical, vivaCT 80, Switzerland). A total of 75 three-dimensional landmarks (Ferreira-Cardoso et al. 2020) were located on pangolin skulls using Stratovan CheckPoint® software. The similar data of 32 skulls from four Asian pangolins (Ferreira-Cardoso et al. 2020) were included in the analyses (Additional File 2: Extended Data Table S1). Data on landmarks was imported in MorphoJ (Klingenberg 2011) to analyze morphological differences of skulls. We performed generalized Procrustes analysis (GPA) (Rohlf and Slice 1990) to eliminate effects of orientation, position, and size, and received centroid size (CS) values (Dryden and Mardia 2016) and matrices of shape coordinates (Procrustes coordinates) (Klingenberg et al. 2002). The shape features of each group were distinguished by canonical variable analysis (CVA). The distance of each group from the sample mean was measured in Mahalanobis distance. Procrustes distances were used to quantify the distance of shape differences between groups, which was an absolute measure of the magnitude of shape differences between groups.

### DNA isolation, library preparation and sequencing

For each pangolin sample, genomic DNA was extracted from muscle and integumentary appendage samples using a standard phenol/chloroform extraction method (Gautam 2022). DNAs with sufficient quantity (>500 ng) were selected for library construction. Paired-end sequencing libraries with an insert size of 350 bp were constructed according to the manufacturer's instructions. The libraries were then subjected to the DNBSEQ-T1 sequencer (BGI, Shenzhen, China) for short-read genome sequencing.

### Species identification and mitochondrial phylogeny construction

For species identification, we generated the whole mitogenomes sequence for each individual with NOVOPlasty (Dierckxsens et al. 2017) (version: 4.3.1). We concatenated 13 mitochondrial coding genes (*ND1*, *ND2*, *ND3*, *ND4*, *ND 4L*, *ND5*, *ND6*, *ATP6*, *ATP8*, *COX1*, *COX2*, *COX3*, *Cytb*) to form a super gene with 594 pangolins sequenced in this study, 91 previously published pangolins with clear species classification, and outgroups (*N* = 687,Table S1 and S2). We aligned the dataset of super gene sequences with MEGA (Kumar et al. 2018) (version: X). The alignment was partitioned using PartitionFinder (Lanfear et al. 2017) (version: 2.1.1). The analysis recommended splitting the dataset into one partition and proposed the best-fit nucleotide substitution model “GTR + I + G” for phylogenetic analysis. We then constructed a maximum-likelihood (ML) tree with 1000 bootstraps by using IQ-TREE (Nguyen et al. 2015) (version: 1.6.6). Tree layout was generated using the online tool Interactive Tree of Life (iTOL, http://itol.embl.de). To further explore the geographical origin of the Malayan pangolin populations, we generated a haplotype network using the Median-Joining method in PopART (Leigh and Bryant 2015) (http://popart.otago.ac.nz/) based on the joint *COX1*-*Cytb* gene of 582 Malayan pangolins sequenced in this study and 131 published Malayan pangolin individuals, some of which have known geographical origins (*N* = 715, Table S16).

To verify the species affiliation of MJ_outlier_ (MJ-DW-89 and MJ-DCW-116) and Asian mysterious pangolin newly proposed by Gu et al.(2023), we included 172 mitogenomes sequences covering nine pangolin species (Additional File 1: Table S7), including the aforementioned Indian pangolin, Chinese pangolin, Malayan pangolin, Philippine pangolin, white-bellied pangolin, black-bellied pangolin, giant pangolin, Temminck's pangolin, and Asian mysterious pangolin (Hu et al. 2020b; Cao et al. 2021; Gu et al. 2023; Heighton et al. 2023). Using the above method, a supergene sequence consisting of 13 coding genes was obtained, an ML tree with 1000 bootstraps was constructed at the mitochondrial gene level. We selected the domestic cat (*Felis catus*) (Lopez et al. 1996) and domestic dog (*Canis familiaris*) (Kim et al. 1998) as the outgroup species, and their genome sequences were downloaded from NCBI (Additional File 1: Table S7).

### Genome mapping and variants calling

Reads of 10 Mb were randomly extracted from each sample to conduct DNA damage assessment by mapDamage2.0 (Jónsson et al. 2013). We used the Burrows-Wheeler Aligner (Li and Durbin 2010) (BWA, version: 0.7.10-r789) to map sequencing data to the human (*Homo sapiens*) genome (T2T-CHM13v2.0, GenBank: GCF_009914755.1) (Miga et al. 2020) to evaluate contamination from modern humans. Raw sequence reads were aligned to the *M. javanica* reference genome (MJ_LKY, GenBank: GCF_040802235.1) (Lan et al. 2025) using the Burrows-Wheeler Aligner (Li and Durbin 2010) (BWA, version: 0.7.10-r789) mem algorithm with default parameters. Sequencing coverage of each individual was calculated with Samtools (Danecek et al. 2021) (version: 1.3). Individuals with coverage < 80% were discarded. Next, Picard tools (http://picard.sourceforge.net) (version: 2.1.1) and Genome Analysis Toolkit (DePristo et al. 2011) (GATK, version: 4.0.3.0) were employed to process and filter the BAM format alignment file, including sorting, removing duplicates, and local realignment. Raw variant calling for each individual was then performed by HaplotypeCaller implemented in the GATK, with a Genome Variant Call Format (gVCF) file generated for each individual. Variant joint calling was then performed by combining all gVCF files into a population level VCF file. We finally obtained a set of SNPs from joint-calling variants by GATK with the parameter “SelectVariants – select-type-to-include SNP.”

We then performed a series of analyses to filter the SNPs set. (1) We first performed hard filtering with parameters of “QUAL < 30.0 || QD < 2.0 || FS > 60.0 || MQ < 40.0 || MQRankSum < −12.5 || ReadPosRankSum < −8.0.” (2) SNP sites with extreme depths value (> 99.75% and < 0.25%) were then filtered. (3) Loci with a quality value (PL) greater than 20 and a missing rate less than 0.1 were retained using VCFtools (Danecek et al. 2011) (version: 0.1.13). (4) The final high-quality SNP set was used for further population genetic analyses. For PCA, admixture, and phylogenetic analysis, the SNP set was pruned with VCFtools and the parameter “–thin 2000.” We inferred the family relationship among all Malayan pangolins with KING (Manichaikul et al. 2010) (version: 2.2.7) to remove the potential consanguineous individuals (parent-offspring, monozygotic twin, full-sibling, and 2nd degree) and retained all unrelated individuals for the subsequent analyses (total 598 individuals).

### PCA, phylogenetic tree, and admixture analyses

PCA was performed using Genome-wide Complex Trait Analysis (Yang et al. 2011) (GCTA, version: 1.91.4beta3) software. The ML phylogenetic tree was constructed with 1000 bootstraps using IQ-TREE and the tree layout was generated using the online tool iTOL. The admixture clustering analysis was analyzed with the cluster number K ranging from 2 to 20 by ADMIXTURE (Alexander et al. 2009) (version: 1.3.0).

For the Asian mysterious pangolin identification, we performed PCA and phylogeny on the large population (*N* = 598) of this study (Additional File 1: Figure S1, Table S1 and S5). Two special individuals (MJ-DCW-89/MJ-DCW-116) were named MJ_outlier_ population. We then performed PCA, phylogeny, and admixture clustering analyses among nine pangolin species, including 141 genome sequences, 139 of which are published (Additional File 1: Table S7) and the remaining two are MJ_outlier_ population. The phylogenetic relationships of nine pangolin species were identified by comparing the topological structures of the trees constructed from autosomal SNPs and mitochondrial genes.

For determining the population structure of Malayan pangolins (MJ_main_, *N* = 596), we performed PCA, phylogeny, and admixture clustering analyses (Additional File 1: Figure S1, Table S1 and S5). In addition, 10 samples were selected from each of the MJ2 and MJ3 populations for population structure analysis, this process was repeated three times to avoid errors caused by differences in the number of different populations. R software was used for sampling, and the random number seed was set to 1115.

### Genetic difference and gene flow among different populations

We used Weir and Cockerham's *F*_ST_ to estimate genetic differences, including the differentiation between MJ_outlier_ and the MJ_main_ (*N* = 598), differences between each pair of nine pangolin species (*N* = 141), and the pairwise differentiation among three Malayan pangolin populations (*N* = 596). All bi-allelic SNPs were used for the calculation of genome-wide *F*_ST_ with the parameter “–fst-window-size 50000 –fst-window-step 10000” using VCFtools.

We applied the classic ABBA-BABA (D statistics) to explore the occurrence of gene flow by the software ADMIXTOOLS (version: 5.1) (Patterson et al. 2012) qpDstat module to calculate the number of shared alleles. Chinese pangolin was used as the outgroup (MP), according to the phylogenetic tree. The calculation range of Z-score is the absolute value greater than 3. IBD fragments were screened by RefinedIBD (version: 16May19. ad5) with default parameters. We compared the mean values of IBD fragments within each population at the individual pair level to assess the extent of recent gene flow.

### Demographic analysis

We first used MSMC2 (Schiffels and Durbin 2014) (version: 2.1.2) to infer the separation time and effective population size (*N*_e_) for the three Malayan pangolin populations. Genotype phasing was performed by using Beagle (version: 5.0) software with default parameters before the MSMC2 inference. The population history was inferred using the following parameters: -R -i 20 -t 6 -p “10*1 + 15*2”, with 4 independent replications and 4 randomly selected samples from each population. The calculation parameters of population separation time are as follows: –skipAmbiguous -I 0-3, 0-4, 0-5, 1-3, 1-4, 1-5, 2-3, 2-4, 2-5 -i 20 -t 6 -p “10*1 + 15*2”. The relative cross coalescence rate (RCCR) was calculated using a python script (combineCrossCoal.py) and visualized using a Perl script. The mutation rate and generation -val of *M. javanica* we used here was 1.47 × 10^−8^ per site per generation (Nash et al. 2018) and 1 year (Wei et al. 2024) to visualize.

We used fastsimcoal (version 2.7.0.9) to model demographic history of the three pangolin populations (Excofffier et al. 2021). We first estimated the folded site frequency spectrum (3d-SFS) using easySFS (https://github.com/isaacovercast/easySFS), including only sites without missing data, with a minimum of ten reads supporting all genotypes and a minimum of two reads supporting each allele of heterozygous genotypes. We then considered three distinct demographic models (see Figure S15): (A) Model 1—Divergence of MJ1 followed by the divergence of MJ2 and MJ3; (B) Model 2—Divergence of MJ3 followed by the divergence of MJ1 and MJ2; (C) Model 3—Simultaneous divergence of MJ1, MJ2, and MJ3. To optimize each model, we conducted 100 independent runs. Simulations for all fastsimcoal2 models were conducted with the following parameters: -m -n 100000 -L 40 -s 0 -M -c 12, with a mutation rate of 1.47 × 10⁻⁸. Model comparison was performed using Akaike Information Criterion (AIC) values, calculated via custom R scripts.

### Genetic diversity, ROH, and mutational load

Genome-wide genetic diversity (*He* and π) were computed by the VCFtools in a nonoverlap 50 kb window. ROHs were identified for each individual using the “run of homozygosity” function in PLINK (Purcell et al. 2007) (version: 1.90b4.6). After referring to other pangolin research literature (Gu et al. 2023; Wei et al. 2024), we selected the parameters here as: –homozyg-window-snp 20 –homozyg-kb 100 –homozyg-density 50.We compared the total length of ROH fragments (ROH > 100 kb, 100 kb < ROH < 1 Mb, and ROH > 1 Mb) and their fraction in the reference genome (F_ROH_) among populations. We calculated the relationship between ROH length (cM) and the number of generations they generated with the recombination rate (1.9 cM/Mb) used in Li et al. (2016).

We identified derived mutational loads with all available pangolin genomes as the reference, including: *Manis crassicaudata* (GCA_032201225.1), *Manis culionensis* (GCA_032206105.1), *M. Pentadactyla* (GCA_040802205.1), *Phataginus Tetradactyla* (GCA_032200785.1), *Phataginus Tricuspis* (GCA_029783875.1), *Smutsia Gigantea* (GCA_032199065.1), and *Smutsia Temminckii* (GCA_032201145.1). For LoFs, we annotated derived mutations in coding regions of each pangolin individual via annotation by SnpEff (version: 4.3) (Cingolani et al. 2012). LoFs included *splice_donor_variant*, *splice_acceptor_variant*, and *stop_gained*. We considered “*missence_variant*” as missense mutation, which was annotated through SnpEff (Cingolani et al. 2012). The proportion of homozygous mutations was calculated with the formula: 2 * homozygous sites/(2 * homozygous sites + heterozygous site). In addition, we characterized the genetic purging effect of the Malayan pangolin populations by comparing the frequencies of LoF mutations within and outside the ROH, specifically by normalizing the number of LoF mutations by the number of synonymous mutations in the same region.

## Supplementary Material

msag016_Supplementary_Data

## Data Availability

The data that support the findings of this study have been deposited into CNGB Sequence Archive (CNSA) of the China National GeneBank DataBase (CNGBdb) with accession number CNP0002036.

## References

[msag016-B1] Alexander DH, Novembre J, Lange K. Fast model-based estimation of ancestry in unrelated individuals. Genome Res. 2009:19:1655–1664. 10.1101/gr.094052.109. 19648217 PMC2752134

[msag016-B2] Bai H et al Whole-genome sequencing of 175 Mongolians uncovers population-specific genetic architecture and gene flow throughout north and east Asia. Nat Genet. 2018:50:1696–1704. 10.1038/s41588-018-0250-5. 30397334

[msag016-B4] Buckland S et al High risks of losing genetic diversity in an endemic Mauritian gecko: implications for conservation. PLoS One. 2014:9:e93387. 10.1371/journal.pone.0093387. 24963708 PMC4070904

[msag016-B5] Cao P et al Genome-wide signatures of mammalian skin covering evolution. Sci China Life Sci. 2021:64:1765–1780. 10.1007/s11427-020-1841-5. 33481165

[msag016-B6] Challender D et al *Manis javanica*. *IUCN Red List Threat Species*. 2019:e.T12763A123584856; 2019. 10.2305/IUCN.UK.2019-3.RLTS.T12763A123584856.en

[msag016-B7] Cingolani P et al A program for annotating and predicting the effects of single nucleotide polymorphisms, SnpEff. Fly (Austin). 2012:6:80–92. 10.4161/fly.19695. 22728672 PMC3679285

[msag016-B8] Danecek P et al The variant call format and VCFtools. Bioinformatics. 2011:27:2156–2158. 10.1093/bioinformatics/btr330. 21653522 PMC3137218

[msag016-B9] Danecek P et al Twelve years of SAMtools and BCFtools. Gigascience. 2021:10:giab008. 10.1093/gigascience/giab008. 33590861 PMC7931819

[msag016-B10] DePristo MA et al A framework for variation discovery and genotyping using next-generation DNA sequencing data. Nat Genet. 2011:43:491–498. 10.1038/ng.806. 21478889 PMC3083463

[msag016-B11] Dierckxsens N, Mardulyn P, Smits G. NOVOPlasty: de novo assembly of organelle genomes from whole genome data. Nucleic Acids Res. 2017:45:e18. 10.1093/nar/gkw955. 28204566 PMC5389512

[msag016-B12] Dryden IL, Mardia KV. Statistical shape analysis: with applications in R. John Wiley and Sons; 2016.

[msag016-B13] Excofffier L, Marchi N, Marques DA, Matthey-Doret R, Sousa VC. Fastsimcoal2: demographic inference under complex evolutionary scenarios. Bioinformatics. 2021:37:4882–4885. 10.1093/bioinformatics/btab468. 34164653 PMC8665742

[msag016-B14] Ferreira-Cardoso S, Billet G, Gaubert P, Delsuc F, Hautier L. Skull shape variation in extant pangolins (Pholidota: Manidae): allometric patterns and systematic implications. Zool J Linn Soc. 2020:188:255–275. 10.1093/zoolinnean/zlz096

[msag016-B15] Gaubert P, Antunes A. Assessing the taxonomic status of the Palawan pangolin *Manis culionensis* (Pholidota) using discrete morphological characters. J Mammal. 2005:86:1068–1074. 10.1644/1545-1542(2005)86[1068:ATTSOT]2.0.CO;2.

[msag016-B16] Gaubert P et al The complete phylogeny of pangolins: scaling up resources for the molecular tracing of the most trafficked mammals on earth. J Hered. 2018:109:347–359. 10.1093/jhered/esx097. 29140441

[msag016-B3] Gautam A. Phenol-chloroform DNA isolation method. In: DNA and RNA Isolation Techniques for Non-Experts. Techniques in Life Science and Biomedicine for the Non-Expert. Springer, Cham. 2022. 10.1007/978-3-030-94230-4_3.

[msag016-B17] Gu TT et al Genomic analysis reveals a cryptic pangolin species. Proc Natl Acad Sci U S A. 2023:120:e2304096120. 10.1073/pnas.2304096120. 37748052 PMC10556634

[msag016-B18] Hamilton R et al Forest mosaics, not savanna corridors, dominated in Southeast Asia during the last glacial maximum. Proc Natl Acad Sci U S A. 2024:121:8. 10.1073/pnas.2311280120. PMC10769823 38147645

[msag016-B19] Heighton SP et al Pangolin genomes offer key insights and resources for the world's most trafficked wild mammals. Mol Biol Evol. 2023:40:msad190. 10.1093/molbev/msad190. 37794645 PMC10551234

[msag016-B20] Heinrich S et al Where did all the pangolins go? International CITES trade in pangolin species. Glob Ecol Conserv. 2016:8:241–253. 10.1016/j.gecco.2016.09.007

[msag016-B21] Hong DY . Gen-morph species concept-A new and integrative species concept for outbreeding organisms. J Syst Evol. 2020:58:725–742. 10.1111/jse.12660.

[msag016-B22] Hu J, Roos C, Lv X, Kuang W, Yu L. Molecular genetics supports a potential fifth Asian pangolin species (Mammalia, Pholidota, *Manis*). Zool Sci. 2020a:37:538–543. 10.2108/zs200084. 33269869

[msag016-B23] Hu JY et al Genomic consequences of population decline in critically endangered pangolins and their demographic histories. Natl Sci Rev. 2020b:7:798–814. 10.1093/nsr/nwaa031. 34692098 PMC8288997

[msag016-B24] Jiang ZG, Liu SY, Wu Y, Jiang XL, Zhou KY. China's mammal diversity. Biodivers Sci. 2017:25:886–895. 10.17520/biods.2017098.

[msag016-B25] Jónsson H, Ginolhac A, Schubert M, Johnson PLF, Orlando L. mapDamage2. 0: fast approximate Bayesian estimates of ancient DNA damage parameters. Bioinformatics. 2013:29:1682–1684. 10.1093/bioinformatics/btt193. 23613487 PMC3694634

[msag016-B26] Kim KS, Lee SE, Jeong HW, Ha JH. The complete nucleotide sequence of the domestic dog (*Canis familiaris*) mitochondrial genome. Mol Phylogenet Evol. 1998:10:210–220. 10.1006/mpev.1998.0513. 9878232

[msag016-B27] Klingenberg CP . Morphoj: an integrated software package for geometric morphometrics. Mol Ecol Resour. 2011:11:353–357. 10.1111/j.1755-0998.2010.02924.x. 21429143

[msag016-B28] Klingenberg CP, Barluenga M, Meyer A. Shape analysis of symmetric structures: quantifying variation among individuals and asymmetry. Evolution. 2002:56:1909–1920. 10.1111/j.0014-3820.2002.tb00117.x. 12449478

[msag016-B29] Kumar S, Stecher G, Li M, Knyaz C, Tamura K. MEGA X: molecular evolutionary genetics analysis across computing platforms. Mol Biol Evol. 2018:35:1547–1549. 10.1093/molbev/msy096. 29722887 PMC5967553

[msag016-B30] Lan T et al Enhancing inbreeding estimation and global conservation insights through chromosome-level assemblies of the Chinese and malayan pangolin. GigaScience. 2025:14:giaf003. 10.1093/gigascience/giaf003. 39947250 PMC11825179

[msag016-B31] Lanfear R, Frandsen PB, Wright AM, Senfeld T, Calcott B. PartitionFinder 2: new methods for selecting partitioned models of evolution for molecular and morphological phylogenetic analyses. Mol Biol Evol. 2017:34:772–773. 10.1093/molbev/msw260. 28013191

[msag016-B32] Leigh JW, Bryant D. POPART: full-feature software for haplotype network construction. Methods Ecol Evol. 2015:6:1110–1116. 10.1111/2041-210X.12410.

[msag016-B33] Li G et al A high-resolution SNP array-based linkage map anchors a new domestic cat draft genome assembly and provides detailed patterns of recombination. G3: Genes Genomes Genet. 2016:6:1607–1616. 10.1534/g3.116.028746. PMC4889657 27172201

[msag016-B34] Li H, Durbin R. Fast and accurate long-read alignment with Burrows-Wheeler transform. Bioinformatics. 2010:26:589–595. 10.1093/bioinformatics/btp698. 20080505 PMC2828108

[msag016-B35] Liu Y, Weng Q. Fauna in decline: plight of the pangolin. Science. 2014:345:884–884. 10.1126/science.345.6199.884-a. 25146276

[msag016-B36] Lopez JV, Cevario S, O'Brien SJ. Complete nucleotide sequences of the domestic cat (*Felis catus*) mitochondrial genome and a transposed mtDNA tandem repeat (numt) in the nuclear genome. Genomics. 1996:33:229–246. 10.1006/geno.1996.0188. 8660972

[msag016-B37] Manichaikul A et al Robust relationship inference in genome-wide association studies. Bioinformatics. 2010:26:2867–2873. 10.1093/bioinformatics/btq559. 20926424 PMC3025716

[msag016-B38] Martinez-Garcia A et al Southern Ocean dust-climate coupling over the past four million years. Nature. 2011:476:312–315. 10.1038/nature10310. 21814203

[msag016-B39] Miga KH et al Telomere-to-telomere assembly of a complete human X chromosome. Nature. 2020:585:79–84. 10.1038/s41586-020-2547-7. 32663838 PMC7484160

[msag016-B40] Mondol S et al New evidence for hybrid zones of forest and savanna elephants in central and West Africa. Mol Ecol. 2015:24:6134–6147. 10.1111/mec.13472. 26577954

[msag016-B41] Myers N, Mittermeier RA, Mittermeie CG, da Fonseca GAB, Kent J. Biodiversity hotspots for conservation priorities. Nature. 2000:403:853–858. 10.1038/35002501. 10706275

[msag016-B42] Nash HC et al Conservation genomics reveals possible illegal trade routes and admixture across pangolin lineages in Southeast Asia. Conserv Genet. 2018:19:1083–1095. 10.1007/s10592-018-1080-9.

[msag016-B43] Nguyen LT, Schmidt HA, Von Haeseler A, Minh BQ. IQ-TREE: a fast and effective stochastic algorithm for estimating maximum-likelihood phylogenies. Mol Biol Evol. 2015:32:268–274. 10.1093/molbev/msu300. 25371430 PMC4271533

[msag016-B44] Panjang E et al Mapping the distribution of the Sunda pangolin (*Manis javanica*) within natural forest in Sabah, Malaysian Borneo. Glob Ecol Conserv. 2024:52:e02962. 10.1016/j.gecco.2024.e02962

[msag016-B45] Patterson N et al Ancient admixture in human history. Genetics. 2012:19:1065–1093. 10.1534/genetics.112.145037. PMC3522152 22960212

[msag016-B46] Payne J, Francis CM. A field guide to mammals of borneo. The Sabah Society; 2007.

[msag016-B47] Pratiknyo H, Ahmad I, Budiyanto BH. Diversity and abundance of termites along altitudinal gradient and slopes in mount slamet, central Java, Indonesia. Biodiversitas. 2018:19:1649–1658. 10.13057/biodiv/d190508.

[msag016-B48] Purcell S et al PLINK: a tool set for whole-genome association and population-based linkage analyses. Am J Hum Genet. 2007:81:559–575. 10.1086/519795. 17701901 PMC1950838

[msag016-B49] Robinson JA et al Genomic flatlining in the endangered island fox. Curr Biol. 2016:26:1183–1189. 10.1016/j.cub.2016.02.062. 27112291

[msag016-B50] Rohlf FJ, Slice D. Extensions of the procrustes method for the optimal superimposition of landmarks. Syst Biol. 1990:39:40–59. 10.2307/2992207

[msag016-B51] Schiffels S, Durbin R. Inferring human population size and separation history from multiple genome sequences. Nat Genet. 2014:46:919–925. 10.1038/ng.3015. 24952747 PMC4116295

[msag016-B52] Sitam FT et al Phylogeography of the Sunda pangolin, *Manis javanica*: implications for taxonomy, conservation management and wildlife forensics. Ecol Evol. 2023:13:e10373. 10.1002/ece3.10373. 37593756 PMC10427774

[msag016-B53] Stuart YE, Losos JB, Algar AC. The island–mainland species turnover relationship. Proc Royal Soc B: Biol Sci. 2012:279:4071–4077. 10.1098/rspb.2012.0816. PMC3427569 22874754

[msag016-B54] Wei S et al Conservation genomics of the critically endangered Chinese pangolin. Sci China Life Sci. 2024:67:2051–2061. 10.1007/s11427-023-2540-y. 38970727

[msag016-B55] Wright N, Jimerson J. The rescue, rehabilitation and release of pangolins. In: Challender DWS, Nash HC, Waterman C, editors. Pangolins. Academic Press; 2020. p. 495–504.

[msag016-B56] Xing S et al Meat and medicine: historic and contemporary use in Asia. In: Challender DWS, Nash HC, Waterman C, editors. Pangolins. Academic Press; 2020. p. 227–239.

[msag016-B57] Yang J, Lee SH, Goddard ME, Visscher PM. GCTA: a tool for genome-wide complex trait analysis. Am J Hum Genet. 2011:88:76–82. 10.1016/j.ajhg.2010.11.011. 21167468 PMC3014363

[msag016-B58] Zhang H et al Molecular tracing of confiscated pangolin scales for conservation and illegal trade monitoring in Southeast Asia. Glob Ecol Conserv. 2015:4:414–422. 10.1016/j.gecco.2015.08.002

