# Supplementary material for: Population genomics reveals deep diversification in Malayan pangolins
Source: Mol Biol Evol. 2026 Jan 16;43(2):msag016. doi: 10.1093/molbev/msag016 (PMC12888049; doi:10.1093/molbev/msag016)
Supplement: msag016_Supplementary_Data [file msag016_supplementary_data.docx]

**Tables of Contents:**

**Supplementary Tables 1 to 21**

**Supplementary Figures 1 to 19**

**Reference for supplementary information**

**Supplementary Tables**

**Supplementary** **Table 1. Sequencing sample information for this study (*N* = 594)**.

| **Species** | **ID** | **Numbers** | **Type** | **Location** | **Population** |
| --- | --- | --- | --- | --- | --- |
| *Smutsia gigantea* | PA-DCW-3 | 1 | Skin | Africa | Outgroup |
| *Phataginus tricuspis* | PA-DCW-6, PA-DCW-10, PA-DCW-11 | 3 | Skin | Africa | Outgroup |
| *Manis pentadactyla* | PA-DCW-1, CSJ-369 | 2 | Skin, Muscle | China | Outgroup |
| *Manis crassicaudata* | P442993, P442994 | 2 | Muscle | Pakistan | Outgroup |
|  | P442983, P442984 | 2 | Muscle | Sri Lanka |  |
| *Manis javanica* | CSJ-149, CSJ-157, CSJ-167, CSJ-168, CSJ-170, CSJ-186, CSJ-189, CSJ-192, CSJ-(196-200), CSJ-203, CSJ-(207-208), CSJ-(211-212), CSJ-(215-217), CSJ-302, CSJ-306, CSJ-308, CSJ-(315-316), CSJ-(321-322), CSJ-(327-328), CSJ-332, CSJ-337, CSJ-346, CSJ-349, CSJ-355, CSJ-375, CSJ-376, CSJ-68, CSJ-81, CSJ-83 | 39 | Muscle | / | Coverage  < 80% |
| *Manis javanica* | CSJ-18, CSJ-67, CSJ-76, CSJ-94, CSJ-178, CSJ-331, MJ-DCW-(14-15), MJ-DCW-19, MJ-DCW-22, MJ-DCW-24, MJ-DCW-26, MJ-DCW-35, MJ-DCW-77, MJ-DCW-85, MJ-DCW-(87-88), MJ-DCW-102, MJ-DCW-(109-110) | 20^#^ | Muscle | / | Close individuals (kinship coefficients > 0.180) |
| *Manis mysteria* | MJ-DCW-89/116 | 2* | Muscle | Southeast Asia | MJoutlier (*M. mysteria*) |
| *Manis javanica* | MJ-DCW-21, MJ-DCW-23, CSJ-439, CSJ-255, CSJ-151 | 5* | Muscle | Southeast Asia | MJmain (MJ1) |
|  | P442988, P442989 | 2* | Muscle | Malaysia | MJmain (MJ1) |
|  | CSJ-252, MJ-DCW-10, MJ-DCW-105, MJ-DCW-106, MJ-DCW-108, MJ-DCW-113, MJ-DCW-114, MJ-DCW-12, MJ-DCW-122, MJ-DCW-16, MJ-DCW-25, MJ-DCW-28, MJ-DCW-38, MJ-DCW-40, MJ-DCW-64, MJ-DCW-66, MJ-DCW-78, MJ-DCW-86, MJ-DCW-90, MJ-DCW-91, PA-DCW-8 | 21* | Muscle | Southeast Asia | MJmain (MJ2) |
|  | P442990 | 1* | Muscle | Malaysia | MJmain (MJ2) |
|  | CSJ-(1-17), CSJ-(19-59), CSJ-(61-66), CSJ-69, CSJ-(71-75), CSJ-(77-80), CSJ-82, CSJ-(84-85), CSJ-(87-91), CSJ-93, CSJ-(95-127), CSJ-(129-135), CSJ-(128-147), CSJ-(150-152), CSJ-154, CSJ-(158-164), CSJ-169, CSJ-171, CSJ-(175-177), CSJ-179, CSJ-181, CSJ-191, CSJ-194, CSJ-199, CSJ-201, CSJ-202, CSJ-(205-206), CSJ-(209-210), CSJ-213, CSJ-(218-220), CSJ-(222-236), CSJ-(238-300), CSJ-(303-305), CSJ-307, CSJ-(309-311), CSJ-(313-314), CSJ-319, CSJ-(329-330), CSJ-333, CSJ-335, CSJ-340, CSJ-348, CSJ-350, CSJ-(352-353), CSJ-(357-359), CSJ-362, CSJ-(364-365) , CSJ-(367-368), CSJ-(371-374), CSJ-(377-380), CSJ-(382-386), CSJ-388, CSJ-390, CSJ-(393-410), CSJ-(412-415), CSJ-(417-422), CSJ-(424-428), CSJ-(430-439), CSJ-441, CSJ-(443-446), CSJ-449, CSJ-(451-456), CSJ-(458-470), CSJ-(472-475), CSJ-(447-484), MJ-DCW-(1-9), MJ-DCW-11, MJ-DCW-13, MJ-DCW-(17-18), MJ-DCW-20, MJ-DCW-27, MJ-DCW-(29-34), MJ-DCW-(36-37), MJ-DCW-39, MJ-DCW-(41-63), MJ-DCW-65, MJ-DCW-(67-76), MJ-DCW-(79-84), MJ-DCW-(92-101), MJ-DCW-(103-104), MJ-DCW-107, MJ-DCW-(111-112), MJ-DCW-115, MJ-DCW-117, MJ-DCW-121, MJ-DCW-127, MJ-DCW-142, MJ-DCW-144, MJ-DCW-146, PA-DCW-5, PA-DCW-7 | 487* | Muscle | Southeast Asia | MJmain (MJ3) |
|  | P10594, P10595, P10596, P10600 | 4* | Muscle | Singapore | MJmain (MJ3) |
|  | P336744 | 1* | Muscle | Cambodia | MJmain (MJ3) |
|  | P442979, P442980 | 2* | Muscle | Indonesia | MJmain (MJ3) |

Note: Muscle and skin samples from Southeast Asia or Africa with no detail location were confiscated by Chinese Customs. ^#^close individuals (*N* = 20, kinship coefficients > 0.180) samples identified and removed by the kinship analyses (**Supplementary Table 3**). * Individuals used in the whole genome analysis of the Malayan pangolin population.

**Supplementary Table 2. Samples used** **for species identification analysis.**

**91 pangolin samples and outgroups (cat and dog) from databases or published studies were used for preliminary species identification of the 594 pangolin individuals sequenced in this study (Supplementary Figure 2, *N* = 687).**

| **Sample name** | **NCBI ID** | **Species** | **reference** |
| --- | --- | --- | --- |
| PA | / | *Manis mysteria* | ^1^ |
| *Felis catus* | NC_001700.1 | *Felis catus* | ^2^ |
| *Canis lupus familiaris* | NC_002008.4 | *Canis familiaris* | ^3^ |
| *S. temminckii* (KP306516.1) | KP306516.1 | *Smutsia temminckii* | ^4^ |
| *P. tricuspis* (NC026780.1) | NC026780.1 | *Phataginus tricuspis* | ^4^ |
| *M. javanica* (NC026781.1) | NC_026781.1 | *Manis javanica* | ^4^ |
| *M. pentadactyla* (MG196307.1) | MG196307.1 | *Manis pentadactyla* | ^4^ |
| GAEM0006 | / | *Manis pentadactyla* | ^5^ |
| GAFM0021 | / | *Manis pentadactyla* | ^5^ |
| GAEM0016 | / | *Manis pentadactyla* | ^5^ |
| GAEM0015 | / | *Manis pentadactyla* | ^5^ |
| GAEM0012 | / | *Manis pentadactyla* | ^5^ |
| *M. crassicaudata* | NC_036433.1 | *Manis crassicaudata* | ^6^ |
| *M. crassicaudata* (MG196306.1) | MG196306.1 | *Manis crassicaudata* | ^6^ |
| *M. crassicaudata* (MG196305.1) | MG196305.1 | *Manis crassicaudata* | ^6^ |
| *M. crassicaudata* (MG196304.1) | MG196304.1 | *Manis crassicaudata* | ^6^ |
| *M. culionensis* (NC036434.1) | NC_036434.1 | *Manis culionensis* | ^6^ |
| *M. culionensis* (MG196308.1) | MG196308.1 | *Manis culionensis* | ^6^ |
| *S. gigantea* (MG196303.1) | MG196303.1 | *Smutsia gigantea* | ^6^ |
| *P. tetradactyla* (MG196299.1) | MG196299.1 | *Phataginus tetradactyla* | ^6^ |
| MJ-PRE-(1-72) | SRR9018586 ~ SRR9018675 | *Manis javanica* | ^7^ |
| MJ-PRE-73 | SRR3949728 | *Manis javanica* | ^8^ |

**Supplementary Table 3. Statistical information of kinship between individuals.** Individuals with bold font were deleted **(*N* = 20)** and only unrelated individuals were included in population genomic analysis.

| **ID1** | **ID2** | **N_SNP** | **Kinship** | **PropIBD** | **InfType** |
| --- | --- | --- | --- | --- | --- |
| CSJ-114 | **CSJ-18** | 9086074 | 0.1885 | 0.2964 | 2nd |
| **CSJ-178** | CSJ-179 | 9073455 | 0.4686 | 0.5739 | Dup/MZ |
| **CSJ-331** | CSJ-447 | 9061361 | 0.2170 | 0.3507 | 2nd |
| CSJ-37 | **CSJ-67** | 9089744 | 0.4795 | 0.6577 | Dup/MZ |
| CSJ-5 | **CSJ-94** | 9089185 | 0.2260 | 0.0974 | 3rd |
| CSJ-75 | **CSJ-76** | 9008403 | 0.2339 | 0.2675 | 2nd |
| **MJ-DCW-102** | MJ-DCW-32 | 9090114 | 0.4772 | 0.6797 | Dup/MZ |
| MJ-DCW-105 | **MJ-DCW-19** | 9084921 | 0.4742 | 0.7110 | Dup/MZ |
| MJ-DCW-106 | **MJ-DCW-14** | 9086884 | 0.4770 | 0.6308 | Dup/MZ |
| MJ-DCW-108 | **MJ-DCW-15** | 9086725 | 0.4544 | 0.6792 | Dup/MZ |
| **MJ-DCW-110** | MJ-DCW-122 | 9090566 | 0.4802 | 0.5804 | Dup/MZ |
| **MJ-DCW-109** | MJ-DCW-34 | 9090326 | 0.4767 | 0.5916 | Dup/MZ |
| MJ-DCW-113 | **MJ-DCW-24** | 9085212 | 0.4691 | 0.6070 | Dup/MZ |
| MJ-DCW-107 | **MJ-DCW-77** | 9086831 | 0.4709 | 0.5938 | Dup/MZ |
| MJ-DCW-21 | **MJ-DCW-88** | 9083344 | 0.4782 | 0.6740 | Dup/MZ |
| **MJ-DCW-22** | MJ-DCW-86 | 9086406 | 0.4741 | 0.7517 | Dup/MZ |
| **MJ-DCW-35** | MJ-DCW-74 | 9088663 | 0.4721 | 0.6510 | Dup/MZ |
| MJ-DCW-23 | **MJ-DCW-87** | 9085745 | 0.4784 | 0.6080 | Dup/MZ |
| **MJ-DCW-26** | **MJ-DCW-85** | 9086621 | 0.4723 | 0.6940 | Dup/MZ |
| **MJ-DCW-26** | MJ-DCW-91 | 9086686 | 0.4749 | 0.6860 | Dup/MZ |
| **MJ-DCW-85** | MJ-DCW-91 | 9089970 | 0.4765 | 0.6647 | Dup/MZ |

**Supplementary Table 4.** Proportion of pollution from humans in the raw data generated by this study.

| Sample | Proportion of pollution | Sample | Proportion of pollution | Sample | Proportion of pollution |
| --- | --- | --- | --- | --- | --- |
| CSJ-1 | 0.002483 | CSJ-290 | 0.0024396 | CSJ-50 | 0.0024595 |
| CSJ-10 | 0.0026506 | CSJ-291 | 0.0024673 | CSJ-51 | 0.0024069 |
| CSJ-100 | 0.0024254 | CSJ-292 | 0.0024938 | CSJ-52 | 0.0024155 |
| CSJ-101 | 0.0025203 | CSJ-293 | 0.0028313 | CSJ-53 | 0.0024488 |
| CSJ-102 | 0.0024542 | CSJ-294 | 0.0024493 | CSJ-54 | 0.002439 |
| CSJ-103 | 0.0024615 | CSJ-295 | 0.0028536 | CSJ-55 | 0.0024298 |
| CSJ-104 | 0.0024748 | CSJ-296 | 0.0028225 | CSJ-56 | 0.0024573 |
| CSJ-105 | 0.0023499 | CSJ-297 | 0.002712 | CSJ-57 | 0.0024347 |
| CSJ-106 | 0.0024713 | CSJ-298 | 0.0027343 | CSJ-58 | 0.0023619 |
| CSJ-107 | 0.0025342 | CSJ-299 | 0.0026404 | CSJ-59 | 0.002422 |
| CSJ-108 | 0.0023333 | CSJ-3 | 0.0027837 | CSJ-6 | 0.0026351 |
| CSJ-109 | 0.0024968 | CSJ-30 | 0.0026117 | CSJ-60 | 0.0024128 |
| CSJ-11 | 0.0026133 | CSJ-300 | 0.002841 | CSJ-61 | 0.0024571 |
| CSJ-110 | 0.0024219 | CSJ-302 | 0.0003294 | CSJ-62 | 0.0024676 |
| CSJ-111 | 0.0024467 | CSJ-303 | 0.0025462 | CSJ-63 | 0.0024732 |
| CSJ-112 | 0.0024572 | CSJ-304 | 0.0028827 | CSJ-64 | 0.0025371 |
| CSJ-113 | 0.0025058 | CSJ-305 | 0.0014979 | CSJ-65 | 0.0024225 |
| CSJ-114 | 0.0025177 | CSJ-306 | 0.0001807 | CSJ-66 | 0.0024082 |
| CSJ-115 | 0.0025014 | CSJ-307 | 0.0021448 | CSJ-67 | 0.0024699 |
| CSJ-116 | 0.0022824 | CSJ-308 | 0.0003992 | CSJ-68 | 0.000705 |
| CSJ-117 | 0.0024498 | CSJ-309 | 0.0023796 | CSJ-69 | 0.0016119 |
| CSJ-118 | 0.0024932 | CSJ-31 | 0.0025328 | CSJ-7 | 0.0025082 |
| CSJ-119 | 0.0024618 | CSJ-310 | 0.0027369 | CSJ-70 | 0.0022949 |
| CSJ-12 | 0.0025727 | CSJ-311 | 0.0022866 | CSJ-71 | 0.0025198 |
| CSJ-120 | 0.0024944 | CSJ-312 | 0.0004881 | CSJ-72 | 0.0006306 |
| CSJ-121 | 0.0024393 | CSJ-313 | 0.002408 | CSJ-73 | 0.0025328 |
| CSJ-122 | 0.00245 | CSJ-314 | 0.0025326 | CSJ-74 | 0.0025492 |
| CSJ-123 | 0.0024277 | CSJ-315 | 0.0000297 | CSJ-75 | 0.0025136 |
| CSJ-124 | 0.0024778 | CSJ-316 | 0.0001801 | CSJ-76 | 0.0009259 |
| CSJ-125 | 0.0023897 | CSJ-317 | 0.0013872 | CSJ-77 | 0.0025256 |
| CSJ-126 | 0.0024468 | CSJ-318 | 0.0005907 | CSJ-78 | 0.0024807 |
| CSJ-127 | 0.0023114 | CSJ-319 | 0.0021837 | CSJ-79 | 0.0020541 |
| CSJ-128 | 0.0024507 | CSJ-32 | 0.0025935 | CSJ-8 | 0.0025934 |
| CSJ-129 | 0.0023456 | CSJ-321 | 0.0004046 | CSJ-80 | 0.0025485 |
| CSJ-13 | 0.0026507 | CSJ-322 | 0.0001399 | CSJ-81 | 0.0003444 |
| CSJ-130 | 0.0024084 | CSJ-325 | 0.0012746 | CSJ-82 | 0.0025662 |
| CSJ-131 | 0.0024287 | CSJ-327 | 0.0000412 | CSJ-83 | 0.0006074 |
| CSJ-132 | 0.0024637 | CSJ-328 | 0.0007766 | CSJ-84 | 0.0025085 |
| CSJ-133 | 0.0024036 | CSJ-329 | 0.0025051 | CSJ-85 | 0.0025848 |
| CSJ-134 | 0.0024079 | CSJ-33 | 0.0025748 | CSJ-87 | 0.0024951 |
| CSJ-135 | 0.0022651 | CSJ-330 | 0.0023451 | CSJ-88 | 0.0024807 |
| CSJ-136 | 0.0023971 | CSJ-331 | 0.0023712 | CSJ-89 | 0.0026524 |
| CSJ-138 | 0.002501 | CSJ-332 | 0.000183 | CSJ-9 | 0.0025727 |
| CSJ-139 | 0.0024484 | CSJ-333 | 0.0019776 | CSJ-90 | 0.0025494 |
| CSJ-14 | 0.0026089 | CSJ-335 | 0.0023171 | CSJ-91 | 0.0023998 |
| CSJ-140 | 0.0023968 | CSJ-336 | 0.0016497 | CSJ-92 | 0.0024107 |
| CSJ-141 | 0.0023739 | CSJ-337 | 0.000058 | CSJ-93 | 0.002451 |
| CSJ-142 | 0.0024972 | CSJ-338 | 0.001677 | CSJ-94 | 0.0023973 |
| CSJ-143 | 0.0024917 | CSJ-34 | 0.0025801 | CSJ-95 | 0.0024842 |
| CSJ-144 | 0.0024907 | CSJ-340 | 0.0024081 | CSJ-96 | 0.0024783 |
| CSJ-145 | 0.0025157 | CSJ-346 | 0.0002387 | CSJ-97 | 0.0024417 |
| CSJ-146 | 0.0024472 | CSJ-348 | 0.0024521 | CSJ-98 | 0.002477 |
| CSJ-147 | 0.0025228 | CSJ-349 | 0.0001063 | CSJ-99 | 0.002547 |
| CSJ-149 | 0.0000926 | CSJ-35 | 0.0024349 | MJ-DCW-1 | 0.0023749 |
| CSJ-15 | 0.0026029 | CSJ-350 | 0.0023562 | MJ-DCW-10 | 0.0017568 |
| CSJ-150 | 0.0024749 | CSJ-352 | 0.0019594 | MJ-DCW-100 | 0.0022486 |
| CSJ-151 | 0.0024522 | CSJ-353 | 0.0024295 | MJ-DCW-101 | 0.0023274 |
| CSJ-152 | 0.0025141 | CSJ-355 | 0.0000849 | MJ-DCW-102 | 0.0022711 |
| CSJ-153 | 0.0007715 | CSJ-357 | 0.0023083 | MJ-DCW-103 | 0.00236 |
| CSJ-154 | 0.0024278 | CSJ-358 | 0.001492 | MJ-DCW-104 | 0.0023998 |
| CSJ-156 | 0.0006485 | CSJ-359 | 0.0024234 | MJ-DCW-105 | 0.0024318 |
| CSJ-157 | 0.0004149 | CSJ-36 | 0.0023928 | MJ-DCW-106 | 0.0023735 |
| CSJ-158 | 0.0024947 | CSJ-361 | 0.0014188 | MJ-DCW-107 | 0.0023189 |
| CSJ-159 | 0.0024734 | CSJ-362 | 0.0023725 | MJ-DCW-108 | 0.0024236 |
| CSJ-16 | 0.002597 | CSJ-363 | 0.0018738 | MJ-DCW-109 | 0.0023226 |
| CSJ-160 | 0.0024484 | CSJ-364 | 0.0016253 | MJ-DCW-11 | 0.0021883 |
| CSJ-161 | 0.0021743 | CSJ-365 | 0.0016804 | MJ-DCW-110 | 0.0023915 |
| CSJ-162 | 0.0025698 | CSJ-367 | 0.0018103 | MJ-DCW-111 | 0.0023338 |
| CSJ-163 | 0.0026621 | CSJ-368 | 0.0016997 | MJ-DCW-112 | 0.0023729 |
| CSJ-164 | 0.0022326 | CSJ-369 | 0.001501 | MJ-DCW-113 | 0.0024065 |
| CSJ-166 | 0.0021412 | CSJ-37 | 0.0024555 | MJ-DCW-114 | 0.0023933 |
| CSJ-167 | 0.0000822 | CSJ-370 | 0.001526 | MJ-DCW-115 | 0.0023218 |
| CSJ-168 | 0.0004138 | CSJ-371 | 0.0018569 | MJ-DCW-116 | 0.0019265 |
| CSJ-169 | 0.0027034 | CSJ-372 | 0.0017699 | MJ-DCW-117 | 0.0027012 |
| CSJ-17 | 0.0025481 | CSJ-373 | 0.0018095 | MJ-DCW-12 | 0.002333 |
| CSJ-170 | 0.0001828 | CSJ-374 | 0.001733 | MJ-DCW-121 | 0.0027816 |
| CSJ-171 | 0.0024496 | CSJ-375 | 0.0004227 | MJ-DCW-122 | 0.0025049 |
| CSJ-172 | 0.0007343 | CSJ-376 | 0.0001439 | MJ-DCW-127 | 0.0012083 |
| CSJ-174 | 0.0022574 | CSJ-377 | 0.0017239 | MJ-DCW-13 | 0.0021465 |
| CSJ-175 | 0.0025781 | CSJ-378 | 0.0017833 | MJ-DCW-14 | 0.0022974 |
| CSJ-176 | 0.0009091 | CSJ-379 | 0.0017767 | MJ-DCW-142 | 0.0024351 |
| CSJ-177 | 0.0026613 | CSJ-38 | 0.0024561 | MJ-DCW-144 | 0.002522 |
| CSJ-178 | 0.0028547 | CSJ-380 | 0.0016072 | MJ-DCW-146 | 0.0011321 |
| CSJ-179 | 0.0028402 | CSJ-382 | 0.0018343 | MJ-DCW-15 | 0.0022366 |
| CSJ-18 | 0.0025175 | CSJ-383 | 0.0018266 | MJ-DCW-16 | 0.0022142 |
| CSJ-180 | 0.0007705 | CSJ-384 | 0.0017968 | MJ-DCW-17 | 0.001776 |
| CSJ-181 | 0.0028767 | CSJ-385 | 0.0018214 | MJ-DCW-18 | 0.0023049 |
| CSJ-185 | 0.0014042 | CSJ-386 | 0.0018346 | MJ-DCW-19 | 0.0023839 |
| CSJ-186 | 0.0017157 | CSJ-388 | 0.0017056 | MJ-DCW-2 | 0.0032246 |
| CSJ-188 | 0.0006437 | CSJ-389 | 0.0019346 | MJ-DCW-20 | 0.0023994 |
| CSJ-189 | 0.0067725 | CSJ-39 | 0.0024658 | MJ-DCW-21 | 0.0023459 |
| CSJ-19 | 0.0025996 | CSJ-390 | 0.0017712 | MJ-DCW-22 | 0.0023617 |
| CSJ-191 | 0.0010524 | CSJ-391 | 0.0019361 | MJ-DCW-23 | 0.0023341 |
| CSJ-192 | 0.0038209 | CSJ-392 | 0.0003972 | MJ-DCW-24 | 0.00211 |
| CSJ-194 | 0.0027348 | CSJ-393 | 0.0023853 | MJ-DCW-25 | 0.0023766 |
| CSJ-196 | 0.000437 | CSJ-394 | 0.0024565 | MJ-DCW-26 | 0.0022651 |
| CSJ-197 | 0.0002024 | CSJ-395 | 0.0024012 | MJ-DCW-27 | 0.0024473 |
| CSJ-198 | 0.000192 | CSJ-396 | 0.0025238 | MJ-DCW-28 | 0.0018354 |
| CSJ-199 | 0.0014973 | CSJ-397 | 0.0024372 | MJ-DCW-29 | 0.0023527 |
| CSJ-2 | 0.0024488 | CSJ-398 | 0.0023566 | MJ-DCW-3 | 0.0023336 |
| CSJ-20 | 0.0025914 | CSJ-399 | 0.002434 | MJ-DCW-30 | 0.0023149 |
| CSJ-200 | 0.0005499 | CSJ-4 | 0.00259 | MJ-DCW-31 | 0.0023352 |
| CSJ-201 | 0.0038657 | CSJ-40 | 0.0024664 | MJ-DCW-32 | 0.0022656 |
| CSJ-202 | 0.0025945 | CSJ-400 | 0.0024353 | MJ-DCW-33 | 0.0023071 |
| CSJ-203 | 0.0002456 | CSJ-401 | 0.0024374 | MJ-DCW-34 | 0.0022246 |
| CSJ-205 | 0.0010573 | CSJ-402 | 0.002438 | MJ-DCW-35 | 0.0020005 |
| CSJ-206 | 0.0022382 | CSJ-403 | 0.0024676 | MJ-DCW-36 | 0.0022796 |
| CSJ-207 | 0.0008767 | CSJ-404 | 0.0024845 | MJ-DCW-37 | 0.0019817 |
| CSJ-208 | 0.0002375 | CSJ-405 | 0.0024515 | MJ-DCW-38 | 0.002228 |
| CSJ-209 | 0.0026432 | CSJ-406 | 0.0024496 | MJ-DCW-39 | 0.0022664 |
| CSJ-21 | 0.0025933 | CSJ-407 | 0.002461 | MJ-DCW-4 | 0.0021822 |
| CSJ-210 | 0.0024483 | CSJ-408 | 0.0024511 | MJ-DCW-40 | 0.0021235 |
| CSJ-211 | 0.0002931 | CSJ-409 | 0.0024308 | MJ-DCW-41 | 0.0020839 |
| CSJ-212 | 0.0002864 | CSJ-41 | 0.0024776 | MJ-DCW-42 | 0.0022101 |
| CSJ-213 | 0.0025815 | CSJ-410 | 0.002513 | MJ-DCW-43 | 0.0021495 |
| CSJ-215 | 0.0004896 | CSJ-411 | 0.0019009 | MJ-DCW-44 | 0.0022654 |
| CSJ-216 | 0.0003094 | CSJ-412 | 0.002485 | MJ-DCW-45 | 0.0022385 |
| CSJ-217 | 0.000536 | CSJ-413 | 0.0025112 | MJ-DCW-46 | 0.0022747 |
| CSJ-218 | 0.0028104 | CSJ-414 | 0.0025491 | MJ-DCW-47 | 0.0022143 |
| CSJ-219 | 0.0026182 | CSJ-415 | 0.0024454 | MJ-DCW-48 | 0.0023328 |
| CSJ-22 | 0.0025755 | CSJ-416 | 0.0018944 | MJ-DCW-49 | 0.0022988 |
| CSJ-220 | 0.0026599 | CSJ-417 | 0.0024339 | MJ-DCW-5 | 0.0024135 |
| CSJ-221 | 0.002553 | CSJ-418 | 0.0025134 | MJ-DCW-50 | 0.002375 |
| CSJ-222 | 0.0026464 | CSJ-419 | 0.0025012 | MJ-DCW-51 | 0.002329 |
| CSJ-223 | 0.0026638 | CSJ-42 | 0.0026806 | MJ-DCW-52 | 0.0024264 |
| CSJ-224 | 0.0026309 | CSJ-420 | 0.0025052 | MJ-DCW-53 | 0.0023154 |
| CSJ-225 | 0.0021601 | CSJ-421 | 0.0024462 | MJ-DCW-54 | 0.0021877 |
| CSJ-226 | 0.0021849 | CSJ-422 | 0.0024822 | MJ-DCW-55 | 0.0028634 |
| CSJ-227 | 0.0022546 | CSJ-423 | 0.0018596 | MJ-DCW-56 | 0.0023807 |
| CSJ-228 | 0.0021689 | CSJ-424 | 0.0024906 | MJ-DCW-57 | 0.0021759 |
| CSJ-229 | 0.0021764 | CSJ-425 | 0.0024712 | MJ-DCW-58 | 0.0020543 |
| CSJ-23 | 0.0025481 | CSJ-426 | 0.0025532 | MJ-DCW-59 | 0.0023601 |
| CSJ-230 | 0.0021444 | CSJ-427 | 0.0022844 | MJ-DCW-6 | 0.0022626 |
| CSJ-231 | 0.0021508 | CSJ-428 | 0.0023686 | MJ-DCW-60 | 0.0023634 |
| CSJ-232 | 0.0021702 | CSJ-429 | 0.0025864 | MJ-DCW-61 | 0.0022882 |
| CSJ-233 | 0.0023591 | CSJ-43 | 0.002452 | MJ-DCW-62 | 0.002332 |
| CSJ-234 | 0.0026071 | CSJ-430 | 0.0024274 | MJ-DCW-63 | 0.0025147 |
| CSJ-235 | 0.0022825 | CSJ-431 | 0.0024957 | MJ-DCW-64 | 0.0022359 |
| CSJ-236 | 0.0022399 | CSJ-432 | 0.0025718 | MJ-DCW-65 | 0.0024164 |
| CSJ-237 | 0.0024317 | CSJ-433 | 0.002528 | MJ-DCW-66 | 0.00247 |
| CSJ-238 | 0.0022787 | CSJ-434 | 0.0025231 | MJ-DCW-67 | 0.0020638 |
| CSJ-239 | 0.0021944 | CSJ-435 | 0.0024797 | MJ-DCW-68 | 0.0024727 |
| CSJ-24 | 0.0025432 | CSJ-436 | 0.0025085 | MJ-DCW-69 | 0.0025773 |
| CSJ-240 | 0.0022585 | CSJ-437 | 0.0025215 | MJ-DCW-7 | 0.0022451 |
| CSJ-241 | 0.0022066 | CSJ-438 | 0.0025029 | MJ-DCW-70 | 0.0024909 |
| CSJ-242 | 0.0026461 | CSJ-439 | 0.0025152 | MJ-DCW-71 | 0.0024933 |
| CSJ-243 | 0.0025009 | CSJ-44 | 0.0022408 | MJ-DCW-72 | 0.0023689 |
| CSJ-244 | 0.002612 | CSJ-440 | 0.0018903 | MJ-DCW-73 | 0.0024422 |
| CSJ-245 | 0.0026924 | CSJ-441 | 0.0025364 | MJ-DCW-74 | 0.0024114 |
| CSJ-246 | 0.00258 | CSJ-442 | 0.0019132 | MJ-DCW-75 | 0.0024112 |
| CSJ-247 | 0.0021836 | CSJ-443 | 0.002545 | MJ-DCW-76 | 0.0025064 |
| CSJ-248 | 0.0025688 | CSJ-444 | 0.0026054 | MJ-DCW-77 | 0.0022995 |
| CSJ-249 | 0.0023726 | CSJ-445 | 0.0025146 | MJ-DCW-78 | 0.0024052 |
| CSJ-25 | 0.0025752 | CSJ-446 | 0.0024194 | MJ-DCW-79 | 0.0024357 |
| CSJ-250 | 0.0014986 | CSJ-447 | 0.0019407 | MJ-DCW-8 | 0.0022432 |
| CSJ-251 | 0.0021821 | CSJ-448 | 0.0019579 | MJ-DCW-80 | 0.0024098 |
| CSJ-252 | 0.0022253 | CSJ-449 | 0.0025182 | MJ-DCW-81 | 0.0023677 |
| CSJ-253 | 0.0023436 | CSJ-45 | 0.0024521 | MJ-DCW-82 | 0.0023646 |
| CSJ-254 | 0.00362 | CSJ-450 | 0.0019696 | MJ-DCW-83 | 0.0024185 |
| CSJ-255 | 0.002194 | CSJ-451 | 0.0024665 | MJ-DCW-84 | 0.0023751 |
| CSJ-256 | 0.0021966 | CSJ-452 | 0.0024726 | MJ-DCW-85 | 0.0023567 |
| CSJ-257 | 0.0023042 | CSJ-453 | 0.0024692 | MJ-DCW-86 | 0.0025035 |
| CSJ-258 | 0.0021427 | CSJ-454 | 0.0023798 | MJ-DCW-87 | 0.0024477 |
| CSJ-259 | 0.0023312 | CSJ-455 | 0.0024249 | MJ-DCW-88 | 0.0024674 |
| CSJ-26 | 0.0026481 | CSJ-456 | 0.0024715 | MJ-DCW-89 | 0.0023724 |
| CSJ-260 | 0.0022733 | CSJ-457 | 0.0025593 | MJ-DCW-9 | 0.0022071 |
| CSJ-261 | 0.0020621 | CSJ-458 | 0.0024065 | MJ-DCW-90 | 0.0023899 |
| CSJ-262 | 0.0023286 | CSJ-459 | 0.0024307 | MJ-DCW-91 | 0.0023907 |
| CSJ-263 | 0.0021955 | CSJ-46 | 0.0024363 | MJ-DCW-92 | 0.002415 |
| CSJ-264 | 0.0021984 | CSJ-460 | 0.0023314 | MJ-DCW-93 | 0.0023848 |
| CSJ-265 | 0.0022783 | CSJ-461 | 0.0024037 | MJ-DCW-94 | 0.0024145 |
| CSJ-266 | 0.0026261 | CSJ-462 | 0.002296 | MJ-DCW-95 | 0.0024594 |
| CSJ-267 | 0.0024871 | CSJ-463 | 0.0026446 | MJ-DCW-96 | 0.0023793 |
| CSJ-268 | 0.0026699 | CSJ-464 | 0.0025498 | MJ-DCW-97 | 0.0025076 |
| CSJ-269 | 0.0026302 | CSJ-465 | 0.0025866 | MJ-DCW-98 | 0.0023804 |
| CSJ-27 | 0.0025839 | CSJ-466 | 0.0026157 | MJ-DCW-99 | 0.0023441 |
| CSJ-270 | 0.0025626 | CSJ-467 | 0.0027372 | P10594 | 0.0000416 |
| CSJ-271 | 0.0023563 | CSJ-468 | 0.0022933 | P10595 | 0.0000403 |
| CSJ-272 | 0.0025375 | CSJ-469 | 0.0026649 | P10596 | 0.0000385 |
| CSJ-273 | 0.002357 | CSJ-47 | 0.0024776 | P10600 | 0.0000426 |
| CSJ-274 | 0.0024402 | CSJ-470 | 0.0026518 | P336744 | 0.0020546 |
| CSJ-275 | 0.0022736 | CSJ-471 | 0.002559 | P442979 | 0.0000024 |
| CSJ-276 | 0.0021948 | CSJ-472 | 0.0026235 | P442980 | 0.0000023 |
| CSJ-277 | 0.0027043 | CSJ-473 | 0.002601 | P442983 | 0.000007 |
| CSJ-278 | 0.002474 | CSJ-474 | 0.0025889 | P442984 | 0.000004 |
| CSJ-279 | 0.0022908 | CSJ-475 | 0.0022542 | P442988 | 0.0000019 |
| CSJ-28 | 0.0025477 | CSJ-476 | 0.0026478 | P442989 | 0.0000018 |
| CSJ-280 | 0.0023512 | CSJ-477 | 0.0024619 | P442990 | 0.0000017 |
| CSJ-281 | 0.002411 | CSJ-478 | 0.0025019 | P442993 | 0.0000028 |
| CSJ-282 | 0.0024083 | CSJ-479 | 0.0024546 | P442994 | 0.000005 |
| CSJ-283 | 0.0024395 | CSJ-48 | 0.0023839 | PA-DCW-1 | 0.002238 |
| CSJ-284 | 0.0024408 | CSJ-480 | 0.0019812 | PA-DCW-10 | 0.0018496 |
| CSJ-285 | 0.0022262 | CSJ-481 | 0.0021289 | PA-DCW-11 | 0.0021405 |
| CSJ-286 | 0.0024746 | CSJ-482 | 0.0017181 | PA-DCW-3 | 0.0021883 |
| CSJ-287 | 0.0025211 | CSJ-483 | 0.0023929 | PA-DCW-5 | 0.0024827 |
| CSJ-288 | 0.0024864 | CSJ-484 | 0.0024812 | PA-DCW-6 | 0.0019932 |
| CSJ-289 | 0.002469 | CSJ-49 | 0.0023718 | PA-DCW-7 | 0.0009217 |
| CSJ-29 | 0.0025999 | CSJ-5 | 0.0026248 | PA-DCW-8 | 0.0022474 |

**Supplementary Table 5.** Sample information of 73 Malayan pangolin individuals downloaded from NCBI **(*N* = 73)**. *Sample IDs and population (MJA/MJB) in the published study ^7^. Among them, the MJ-PRE-31 sample was collected from Myanmar, the MJ-PRE-55, MJ-PRE-61 and MJ-PRE-62 samples were collected from Yunnan Province, China, and the MJ-PRE-73 was collected from Malaysia ^8^.

| **Sample ID** | **Previous ID*** | **NCBI SRR ID** | **Sample ID** | **Previous ID*** | **NCBI SRR ID** |
| --- | --- | --- | --- | --- | --- |
| MJ-PRE-1 | MJ67 ^B^ | SRR9018586 | MJ-PRE-37 | MJ28 ^A^ | SRR9018639 |
| MJ-PRE-2 | MJ66 ^B^ | SRR9018587 | MJ-PRE-38 | MJ36 ^A^ | SRR9018640 |
| MJ-PRE-3 | MJ70 ^B^ | SRR9018588 | MJ-PRE-39 | MJ37 ^A^ | SRR9018641 |
| MJ-PRE-4 | MJ69 ^B^ | SRR9018589 | MJ-PRE-40 | MJ34 ^A^ | SRR9018642 |
| MJ-PRE-5 | MJ73 ^B^ | SRR9018597 | MJ-PRE-41 | MJ35 ^A^ | SRR9018643 |
| MJ-PRE-6 | MJ71 ^B^ | SRR9018598 | MJ-PRE-42 | MJ32 ^A^ | SRR9018644 |
| MJ-PRE-7 | MJ72 ^B^ | SRR9018599 | MJ-PRE-43 | MJ33 ^A^ | SRR9018645 |
| MJ-PRE-8 | MJ49 ^B^ | SRR9018610 | MJ-PRE-44 | MJ30 ^A^ | SRR9018646 |
| MJ-PRE-9 | MJ48 ^B^ | SRR9018611 | MJ-PRE-45 | MJ31 ^A^ | SRR9018647 |
| MJ-PRE-10 | MJ47 ^B^ | SRR9018612 | MJ-PRE-46 | MJ38 ^A^ | SRR9018648 |
| MJ-PRE-11 | MJ46 ^B^ | SRR9018613 | MJ-PRE-47 | MJ39 ^A^ | SRR9018649 |
| MJ-PRE-12 | MJ45 ^B^ | SRR9018614 | MJ-PRE-48 | MJ03 ^A^ | SRR9018650 |
| MJ-PRE-13 | MJ44 ^B^ | SRR9018615 | MJ-PRE-49 | MJ02 ^A^ | SRR9018651 |
| MJ-PRE-14 | MJ43 ^B^ | SRR9018616 | MJ-PRE-50 | MJ01 ^A^ | SRR9018652 |
| MJ-PRE-15 | MJ42 ^A^ | SRR9018617 | MJ-PRE-51 | MJ07 ^A^ | SRR9018654 |
| MJ-PRE-16 | MJ41 ^A^ | SRR9018618 | MJ-PRE-52 | MJ06 ^A^ | SRR9018655 |
| MJ-PRE-17 | MJ40 ^A^ | SRR9018619 | MJ-PRE-53 | MJ05 ^A^ | SRR9018656 |
| MJ-PRE-18 | MJ58 ^B^ | SRR9018620 | MJ-PRE-54 | MJ04 ^A^ | SRR9018657 |
| MJ-PRE-19 | MJ59 ^B^ | SRR9018621 | MJ-PRE-55 | MJ09 ^A^ | SRR9018658 |
| MJ-PRE-20 | MJ50 ^B^ | SRR9018622 | MJ-PRE-56 | MJ08 ^A^ | SRR9018659 |
| MJ-PRE-21 | MJ51 ^B^ | SRR9018623 | MJ-PRE-57 | MJ18 ^A^ | SRR9018660 |
| MJ-PRE-22 | MJ52 ^B^ | SRR9018624 | MJ-PRE-58 | MJ19 ^A^ | SRR9018661 |
| MJ-PRE-23 | MJ53 ^B^ | SRR9018625 | MJ-PRE-59 | MJ14 ^A^ | SRR9018662 |
| MJ-PRE-24 | MJ54 ^B^ | SRR9018626 | MJ-PRE-60 | MJ15 ^A^ | SRR9018663 |
| MJ-PRE-25 | MJ55 ^B^ | SRR9018627 | MJ-PRE-61 | MJ16 ^A^ | SRR9018664 |
| MJ-PRE-26 | MJ56 ^B^ | SRR9018628 | MJ-PRE-62 | MJ17 ^A^ | SRR9018665 |
| MJ-PRE-27 | MJ57 ^B^ | SRR9018629 | MJ-PRE-63 | MJ10 ^A^ | SRR9018666 |
| MJ-PRE-28 | MJ25^A^ | SRR9018630 | MJ-PRE-64 | MJ11 ^A^ | SRR9018667 |
| MJ-PRE-29 | MJ24 ^A^ | SRR9018631 | MJ-PRE-65 | MJ12 ^A^ | SRR9018668 |
| MJ-PRE-30 | MJ27 ^A^ | SRR9018632 | MJ-PRE-66 | MJ13 ^A^ | SRR9018669 |
| MJ-PRE-31 | MJ26 ^A^ | SRR9018633 | MJ-PRE-67 | MJ61 ^B^ | SRR9018670 |
| MJ-PRE-32 | MJ21 ^A^ | SRR9018634 | MJ-PRE-68 | MJ60 ^B^ | SRR9018671 |
| MJ-PRE-33 | MJ20 ^A^ | SRR9018635 | MJ-PRE-69 | MJ63 ^B^ | SRR9018672 |
| MJ-PRE-34 | MJ23 ^A^ | SRR9018636 | MJ-PRE-70 | MJ62 ^B^ | SRR9018673 |
| MJ-PRE-35 | MJ22 ^A^ | SRR9018637 | MJ-PRE-71 | MJ65 ^B^ | SRR9018674 |
| MJ-PRE-36 | MJ29 ^A^ | SRR9018638 | MJ-PRE-72 | MJ64 ^B^ | SRR9018675 |
| / | / | / | MJ-PRE-73 | MJ68 ^B^ | SRR3949728 |

**Supplementary Table 6.** Pairwise *F*_ST_ among species, subspecies and populations.

|  | **Organism name** | **Latin**  **name** | **Pairwise *F*_ST_** | **Sources** |
| --- | --- | --- | --- | --- |
| **Species** | *Canis* | *Canis* | Coyote-Red wolf: 0.108,  Coyote-North American gray wolf: 0.153,  North American gray wolf-Red wolf: 0.177 | ^9^ |
|  | Camel | *Camelus* | Dromedaries-Bactrian camels: 0.54-0.64 | ^10^ |
| **Subspecies** | Giant Panda | *Ailuropoda melanoleuca* | QLI-SC: 0.14 | ^11^ |
|  | Muskox | *Ovibos moschatus* | CaMW-CaIS: 0.56,  CaMW-GrEN: 0.62,  CaME-CaIS: 0.51,  CaIS-GrEN: 0.58 | ^12^ |
|  | African Green Monkey | *Chlorocebus* | *C. a. cynosurus*-*C. a. Tantalus*: 0.33,  *C. a. cynosurus*-*C. a. Sabaeus*: 0.45,  C. a. cynosurus-*C. a. pygerythrus*: 0.16,  *C. a. cynosurus*-*C. a. Aethiops*: 0.55,  *C. a. aethiops*-*C. a. Tantalus*: 0.53,  *C. a. Aethiops*-*C. a. pygerythrus*: 0.56,  *C. a. aethiops*-*C. a. Sabaeus*: 0.6,  *C. a. Tantalus*-*C. a. pygerythrus*: 0.35,  *C. a. Tantalus*-*C. a. Sabaeus*: 0.4,  *C. a. Sabaeus*-*C. a. pygerythrus*: 0.46 | ^13^ |
|  | Bactrian Camel | *Camelus bactrianus* | Wild Bactrian camels-Domestic Bactrian camels: 0.27-0.31 | ^10^ |
|  | European Rabbit | *Oryctolagus cuniculus* | *Oryctolagus cuniculus algirus*-*O. c. cuniculus*: 0.084 | ^14^ |
|  | Tiger | *Panthera tigris* | Amur-Bengal: 0.2,  Amur-Sumatran: 0.318,  Amur-Malayan: 0.23,  SCT-AT: 0.29 | ^15,16^ |
| **Population** | Chinese Pangolin | *Manis pentadactyla* | CPA-CPB: 0.101,  CPA-CPC: 0.541,  CPB-CPC: 0.492 | ^5^ |
|  | African Lepaord | *Panthera pardus* | Namibia-Ghan: 0.144,  Namibia-TanzaniaN: 0.134,  Namibia-TanzaniaN: 0.1,  Namibia-TanzaniaW: 0.067, TanzaniaW-TanzaniaN: 0.069 | ^17^ |
|  | Giant Panda | *Ailuropoda melanoleuca* | SC_MSH-SC_QLA: 0.093 | ^11^ |
|  | Gray Wolf | *Canis lupus* | North American gray wolf-Great Lakes wolves: 0.057  Eurasian gray wolf-Great Lakes wolves: 0.076 | ^9^ |
|  | Human | *Homo sapiens* | CEU-YRI: 0.071,  YRI-CHB+JPT: 0.083,  CHB+JPT-CEU: 0.052 | ^18^ |
|  | Domestic Bactrian Camels | *Camelus bactrianus bactrianus* | Among the domestic Bactrian camels: 0.05-0.06 | ^10^ |
|  | [Killer Whales](https://onlinelibrary.wiley.com/doi/full/10.1111/mms.12851) | *Orcinus orca* | NWA-SWA: 0.08,  NWA-NZ: 0.17,  SWA-NZ: 0.12 | ^19^ |
|  | Wild Western Lowland Gorillas | *Gorilla gorilla gorilla* | Odzala-Lobéké: 0.027,  Odzala-Ndoki Forest: 0.042,  Ndoki Forest-Lobéké: 0.031 | ^20^ |
|  | Muskox | *Ovibos moschatus* | CaMW-CaME: 0.14,  CaIN-CaIS: 0.15,  GrEN-GrW: 0.15 | ^12^ |
|  | Forest Musk Deer | *Moschus berezovskii* | WSC-EQL: 0.07,  WSC-WQL: 0.05,  WQL-EQL: 0.02 | ^21^ |
|  | Bengal tigers | *Panthera tigris tigris* | small isolated population-Kaziranga: 0.26,  small isolated population-Kanha: 0.22,  small isolated population-Corbett: 0.28 | ^22^ |
| **This study** | Malayan Pangolin | *Manis javanica* | MJ_outlier_-MJ_main_: 0.7399,  MJ1-MJ2: 0.1323,  MJ1-MJ3: 0.1900,  MJ2-MJ3: 0.0840 | / |

**Supplementary Table 7. Samples used to verify the fifth Asian pangolin species (*Manis mysteria*) in this study (Autosomal SNPs analysis, *N* = 141/mitochondrial gene analysis, *N* = 172, Supplementary Figure 6).**

| Sample ID | Species | For phylogenetic analysis of autosomal SNPs | Sample source |
| --- | --- | --- | --- |
| MP_KT445978, MP_MG196307, MP_MT335859, MP_MZ868226 | Chinese pangolin  (*Manis pentadactyla*) | No, *N* = 4 | GenBank |
| MP01, MP02, MP04, MP05, MP06, MP08, MP09, MP11 ~ MP19, MP21, MP22, MP23 |  | Yes, *N* = 19 | ^7^ |
| MCR_MG196304, MCR_MG196305, MCR_MG196306 | Indian pangolin  (*Manis crassicaudata*) | No, *N* = 3 | GenBank |
| MCR01 ~ MCR20 |  | Yes, *N* = 20 | ^23^ |
| **MJ-DCW-116, MJ-DCW-89** | Cryptic pangolin  (*Manis mysteria*) | Yes, *N* = 10 | This study |
| MSP01 ~ MSP07 |  |  | ^23^ |
| PA |  |  | ^1^ |
| MJ_JN411577, MJ_KP306515, MJ_KT445979, MJ_MG196302, MJ_MG196309 | Malayan pangolin  (*Manis javanica*) | No, *N* = 5 | GenBank |
| **MJ07, MJ10, MJ14, MJ17, MJ22, MJ23, MJ27, MJ32, MJ40, MJ44 , MJ45, MJ46, MJ57, MJ60, MJ65, MJ66, MJ69, MJ70, MJ71, MJ74** |  | Yes, *N* = 20 | ^7^ |
| MCU_MG196308 | Philippine pangolin  (*Manis culionensis*) | No, *N* = 1 | GenBank |
| PLB |  | Yes, *N* = 1 | ^24^ |
| SGI_MF536684, SGI_MG196301, SGI_MG196303 | Giant pangolin  (*Smutsia gigantea*) | No, *N* = 3 | GenBank |
| SGI01 ~ SGI29 |  | Yes, *N* = 29 | ^23^ |
| STE_KP125951, STE_KP306516, STE_MF536685, STE_MF536686, STE_MF536687, STE_MG196300 | Temminck’s pangolin  (*Smutsia temminckii*) | No, *N* = 6 | GenBank |
| STE01, STE02 |  | Yes, *N* = 2 | ^23^ |
| PTR_AJ421454, PTR_KP306514, PTR_MF536683, PTR_MG196296, PTR_MG196297, PTR_MG196298, PTR_MG196310 | White-bellied pangolin  (*Phataginus tricuspis*) | No, *N* = 7 | GenBank |
| PTR01 ~ PTR20 |  | Yes, *N* = 20 | ^23^ |
| PTE_MF509825, PTE_MG196299 | Black-bellied pangolin  (*Phataginus tetradactyla*) | No, *N* = 2 | GenBank |
| PTE01 ~PTR20 |  | Yes, *N* = 20 | ^23^ |
| CAT_KP202278 | Outgroups (*Felis catus*) | / | GenBank |
| DOG_AB499817 | Outgroups (*Canis lupus familiaris*) | / | GenBank |

**Supplementary Table 8. Information of skulls.**

| **Group** | **Skull number** | **Genetic number** | **Group** | **Skull number** | **Genetic number** |
| --- | --- | --- | --- | --- | --- |
| MJ3 | 1 | MJ-DCW-1 | MJ3 | 48 | MJ-DCW-84 |
| MJ3 | 2 | MJ-DCW-2 | MJ3 | 51 | MJ-DCW-54 |
| MJ3 | 3 | MJ-DCW-3 | MJ3 | 53 | MJ-DCW-65 |
| MJ3 | 4 | MJ-DCW-4 | MJ3 | 56 | MJ-DCW-70 |
| MJ3 | 5 | MJ-DCW-5 | MJ3 | 57 | MJ-DCW-71 |
| MJ3 | 10 | MJ-DCW-8 | MJ3 | 58 | MJ-DCW-67 |
| MJ2 | 14 | MJ-DCW-10 | MJ3 | 59 | MJ-DCW-68 |
| MJ3 | 15 | MJ-DCW-11 | MJ3 | 61 | MJ-DCW-73 |
| MJ2 | 16 | MJ-DCW-12 | MJ3 | 67 | MJ-DCW-80 |
| MJ3 | 17 | MJ-DCW-13 | MJ3 | 68 | MJ-DCW-81 |
| MJ3 | 18 | MJ-DCW-14 | MJ3 | 72 | MJ-DCW-45 |
| **MJ_outlier_** | 19 | MJ-DCW-116 | MJ3 | 75 | MJ-DCW-48 |
| MJ3 | 24 | MJ-DCW-17 | MJ3 | 77 | MJ-DCW-55 |
| MJ3 | 26 | MJ-DCW-18 | MJ2 | 79 | MJ-DCW-26 |
| MJ1 | 34 | MJ-DCW-21 | MJ2 | CSJ-252 | CSJ-252 |
| MJ3 | 35 | MJ-DCW-22 | MJ3 | CSJ-253 | CSJ-253 |
| MJ1 | 36 | MJ-DCW-23 | MJ3 | CSJ-254 | CSJ-254 |
| MJ3 | 37 | MJ-DCW-24 | MJ3 | CSJ-256 | CSJ-256 |
| MJ3 | 40 | MJ-DCW-57 | MJ3 | CSJ-330 | CSJ-330 |
| MJ3 | 43 | MJ-DCW-50 | MJ1 | CSJ-439 | CSJ-439 |
| MJ3 | 44 | MJ-DCW-52 | MJ3 | CSJ-88 | CSJ-88 |

**Supplementary Table 9.** **Mahalanobis distances in the MJ_outlier_ and the MJ_main_.**

|  | **MJ_outlier_** | **MJ1** | **MJ2** |
| --- | --- | --- | --- |
| **MJ1** | 9.60 |  |  |
| **MJ2** | 10.27 | 4.40 |  |
| **MJ3** | 9.88 | 5.25 | 3.69 |

**Supplementary Table 10.** **Procrustes distances in the MJ_outlier_ and the MJ_main_.**

|  | **MJ_outlier_** | **MJ1** | **MJ2** |
| --- | --- | --- | --- |
| **MJ1** | 0.0757 |  |  |
| **MJ2** | 0.0765 | 0.0403 |  |
| **MJ3** | 0.0788 | 0.0425 | 0.0246 |

**Supplementary Table 11.** **Mahalanobis distances in** **the MJ_outlier_ and four Asian pangolin species.**

|  | ***M.javanica*** | ***M.culionensis*** | ***M.pentadactyla*** | ***M.crassicaudata*** |
| --- | --- | --- | --- | --- |
| *M.culionensis* | 17.27 |  |  |  |
| *M.pentadactyla* | 22.37 | 16.92 |  |  |
| *M.crassicaudata* | 22.86 | 23.27 | 22.98 |  |
| **MJ_outlier_** | 15.79 | 22.99 | 27.35 | 21.82 |

**Supplementary Table 12. Procrustes distances in the MJ_outlier_ and four Asian pangolin species.**

|  | ***M.javanica*** | ***M.culionensis*** | ***M.pentadactyla*** | ***M.crassicaudata*** |
| --- | --- | --- | --- | --- |
| ***M.culionensis*** | 0.0811 |  |  |  |
| ***M.pentadactyla*** | 0.1008 | 0.1005 |  |  |
| ***M.crassicaudata*** | 0.0983 | 0.0952 | 0.0675 |  |
| **MJ_outlier_** | 0.076 | 0.1138 | 0.1445 | 0.134 |

**Supplementary Table 13. Comparison of Morphological Differences Landmark Site Numbers, Landmark Site Names, and Morphological Differences in MJ1 and MJ2.**

| **Landmarks** | | **Location Name** | **Morphological characteristics** |
| --- | --- | --- | --- |
|  | 3 | Most posterior point of the premaxillary on the midline. | MJ1 is more posterior to the point of the posterior end of the premaxillary on the midline than MJ2. |
| 50 | 27 | Most lateral point of the occipital condyle. | The distance between the most lateral points on either side of the occipital condyles is narrower in MJ2 than in MJ1. |
| 37 | 11 | Most dorsal point of orbital edge. | MJ1 has a narrower orbital margin distance between the two sides than MJ2, and the rim is positioned farther away from the dorsal side. |
| 57 | 71 | Most dorsal max/palatine contact. | MJ1 is positioned closer to the anastomotic end of the skull and farther away from the dorsal aspect of the skull at the dorsal max/palatine contact. |
| 63 | 74 | Intersection between squamosal-parietal-exoccipital. | MJ1 is wider than MJ2 on both sides of the parietal bone. |
|  | 64 | Posteriormost point of the skull roof portion of the supraoccipital. | The posteriormost point of the skull roof portion of the supraoccipital is higher and closer to the end of the muzzle in MJ1 than in MJ2. |
| 65 | 75 | Concavity located above the condyle. | MJ1 has a larger condyle than MJ2. |

**Supplementary Table 14. Comparison of Morphological Differences Landmark Site Numbers, Landmark Site Names, and Morphological Differences in MJ1 and MJ3.**

| **Landmarks** | | **Location Name** | **Morphological characteristics** |
| --- | --- | --- | --- |
| 63 | 74 | Intersection between squamosal-parietal-exoccipital. | MJ1 is wider than MJ3 on both sides of the parietal bone. |
| 61 | 73 | Intersection between frontal-parietal-squamosal. | MJ1 has a larger squamosal bone than MJ3, and the parietal-frontal junction is more distal on both sides of the skull. |
| 57 | 71 | Most dorsal max/palatine contact. | The position of the dorsal max/palatal contact is farther away from the dorsal aspect of the skull in MJ1 than in MJ3. |
|  | 64 | Posteriormost point of the skull roof portion of the supraoccipital. | The posteriormost point of the skull roof portion of the supraoccipital is more distant from the muzzle end in MJ1 than in MJ3. |
| 37 | 11 | Most dorsal point of orbital edge. | MJ1 has a narrower orbital margin distance between the two sides than MJ3, and the rim is positioned farther away from the dorsal side. |
|  | 60 | Intersection between inter-parietal and inter-frontal sutures. | The intersection between inter-parietal and interfrontal sutures is higher and closer to the muzzle end in MJ1 than in MJ3. |

**Supplementary Table 15. Comparison of Morphological Differences Landmark Site Numbers, Landmark Site Names, and Morphological Differences in MJ2 and MJ3.**

| **Landmarks** | | **Location Name** | **Morphological characteristics** |
| --- | --- | --- | --- |
| 61 | 73 | Intersection between frontal-parietal-squamosal. | MJ2 has a wider distance between the parietal and frontal bone junctions on both sides of the skull than MJ3. |
|  | 64 | Posteriormost point of the skull roof portion of the supraoccipital. | The posteriormost point of the skull roof portion of the supraoccipital is more distant from the muzzle end in MJ2 than in MJ3. |
|  | 60 | Intersection between inter-parietal and inter-frontal sutures. | The intersection between inter-parietal and interfrontal sutures is higher in MJ2 than in MJ3. |
|  | 62 | Intersection between interparietal suture with supraoccipital. | The intersection between interparietal suture with supraoccipital is higher in MJ2 than in MJ3. |
|  | 3 | Most posterior point of the premaxillary on the midline. | MJ3 is more posterior to the point of the posterior end of the premaxillary on the midline than MJ2. |

**Supplementary Table 16. Information of 131 published Malayan pangolin individuals used in haplotype network analysis (Fig. 3C). Combined with the 582 Malayan pangolins sequenced in this study (*N* = 582), a total of 715 Malayan pangolins were used for haplotype network analysis to explore the origin and distribution of the Malayan pangolin population (*N* = 715).**

| **Sample name** | **NCBI ID** | **Location** | **reference** |
| --- | --- | --- | --- |
| MG196302.1 | MG196302.1 | Tailand | ^6^ |
| KP306515.1 | KP306515.1 | Tailand | ^4^ |
| KT445979.1 | KT445979.1 | Malaysia | GenBank |
| Singapore_8b | MG825495.1 | Singapore/Sumatra | ^25^ |
| Singapore_1 | MG825496.1 | Singapore/Sumatra | ^25^ |
| MZBR_1190 | MG825497.1 | Java | ^25^ |
| MZBR_1189 | MG825498.1 | Java | ^25^ |
| MZBR_1188 | MG825499.1 | Java | ^25^ |
| MZBR_1055 | MG825500.1 | Borneo | ^25^ |
| MZBR_1087 | MG825501.1 | Borneo | ^25^ |
| MZBR_1078 | MG825502.1 | Singapore/Sumatra | ^25^ |
| MZBR_1043 | MG825503.1 | Borneo | ^25^ |
| MZBR_1064 | MG825504.1 | Borneo | ^25^ |
| MZBR_0274 | MG825505.1 | Singapore/Sumatra | ^25^ |
| MZBR_0276 | MG825506.1 | Borneo | ^25^ |
| MZBR_1074 | MG825507.1 | Java | ^25^ |
| MZBR_0275 | MG825508.1 | Singapore/Sumatra | ^25^ |
| MZBR_1040 | MG825509.1 | Borneo | ^25^ |
| MZBR_1044 | MG825510.1 | Borneo | ^25^ |
| MZBR_1162 | MG825511.1 | Borneo | ^25^ |
| MZBR_1163 | MG825512.1 | Java | ^25^ |
| MZBR_0273 | MG825513.1 | Singapore/Sumatra | ^25^ |
| MZBR_1166 | MG825514.1 | Borneo | ^25^ |
| MZBR_1057 | MG825515.1 | Borneo | ^25^ |
| MZBR_1042 | MG825516.1 | Borneo | ^25^ |
| MZBR_1034 | MG825517.1 | Borneo | ^25^ |
| MZBR_1178 | MG825518.1 | Indonesia | ^25^ |
| MZBR_1076 | MG825519.1 | Java | ^25^ |
| MZBR_1071 | MG825520.1 | Java | ^25^ |
| MZBR_1184 | MG825521.1 | Java | ^25^ |
| MZBR_1181 | MG825522.1 | Java | ^25^ |
| MZBR_1164 | MG825523.1 | Borneo | ^25^ |
| MZBR_1030 | MG825524.1 | Singapore/Sumatra | ^25^ |
| MZBR_1048 | MG825525.1 | Borneo | ^25^ |
| MZBR_1179 | MG825526.1 | / | ^25^ |
| MZBR_1183 | MG825527.1 | Java | ^25^ |
| MZBR_1180 | MG825528.1 | Java | ^25^ |
| MZBR_1038 | MG825529.1 | Borneo | ^25^ |
| MZBR_1072 | MG825530.1 | Java | ^25^ |
| MZBR_1046 | MG825531.1 | Indonesia | ^25^ |
| MZBR_1054 | MG825532.1 | Borneo | ^25^ |
| MZBR_1083 | MG825533.1 | Borneo | ^25^ |
| MZBR_1053 | MG825534.1 | Borneo | ^25^ |
| MZBR_1070 | MG825535.1 | Borneo | ^25^ |
| MZBR_1073 | MG825536.1 | Borneo | ^25^ |
| MZBR_1157 | MG825537.1 | Borneo | ^25^ |
| MZBR_1084 | MG825538.1 | Indonesia | ^25^ |
| MZBR_1052 | MG825539.1 | Borneo | ^25^ |
| MZBR_1165 | MG825540.1 | Indonesia | ^25^ |
| MZBR_1082 | MG825541.1 | Java | ^25^ |
| MZBR_1036 | MG825542.1 | Borneo | ^25^ |
| MZBR_1081 | MG825543.1 | Borneo | ^25^ |
| MZBR_1086 | MG825544.1 | Indonesia | ^25^ |
| MZBR_1050 | MG825545.1 | Indonesia | ^25^ |
| MZBR_1063 | MG825546.1 | Borneo | ^25^ |
| MZBR_1177 | MG825547.1 | Indonesia | ^25^ |
| MZBR_1069 | MG825548.1 | Java | ^25^ |
| MZBR_1085 | MG825549.1 | Borneo | ^25^ |
| MZBR_1182 | MG825550.1 | Borneo | ^25^ |
| MZBR_1077 | MG825551.1 | Indonesia | ^25^ |
| MJ-PRE-(1-30) | SRR9018586 ~ SRR9018589  SRR9018597 ~ SRR9018599  SRR9018610 ~ SRR9018632 | / | ^7^ |
| MJ-PRE-31 | SRR9018633 | Myanmar | ^7^ |
| MJ-PRE-(32-54) | SRR9018634 ~ SRR9018657 | / | ^7^ |
| MJ-PRE-55 | SRR9018658 | Yunnan, China | ^7^ |
| MJ-PRE-(56-60) | SRR9018659 ~ SRR9018663 | / | ^7^ |
| MJ-PRE-61 | SRR9018664 | Yunnan, China | ^7^ |
| MJ-PRE-62 | SRR9018665 | Yunnan, China | ^7^ |
| MJ-PRE-(63-72) | SRR9018666 ~ SRR9018675 | / | ^7^ |
| MJ-PRE-73 | SRR3949728 | Malaysia | ^8^ |

**Supplementary Table 17.** **Haplotype grouping in the Malayan pangolin haplotype network** (**Fig. 3c, *N* = 715**).

| **Haplotype** | **Number** | **Number of samples Sample name** |
| --- | --- | --- |
| Hap_1 | 8 | P10594, P10595, P10596, MJ-DCW-55, MJ-DCW-68, MJ-DCW-83, Singapore8b, Singapore1 |
| Hap_2 | 68 | P10600, CSJ-106, CSJ-109, CSJ-110, CSJ-115, CSJ-127, CSJ-13, CSJ-138, CSJ-139, CSJ-14, CSJ-143, CSJ-145, CSJ-152, CSJ-153, CSJ-157, CSJ-172, CSJ-181, CSJ-191, CSJ-192, CSJ-21, CSJ-216, CSJ-220, CSJ-222, CSJ-224, CSJ-230, CSJ-234, CSJ-244, CSJ-26, CSJ-267, CSJ-271, CSJ-272, CSJ-275, CSJ-28, CSJ-287, CSJ-289, CSJ-291, CSJ-302, CSJ-306, CSJ-309, CSJ-317, CSJ-322, CSJ-327, CSJ-328, CSJ-331, CSJ-336, CSJ-337, CSJ-404, CSJ-408, CSJ-41, CSJ-410, CSJ-414, CSJ-418, CSJ-430, CSJ-433, CSJ-434, CSJ-442, CSJ-448, CSJ-459, CSJ-58, CSJ-7, CSJ-79, CSJ-93, MJ-DCW-46, MJ-DCW-56, MJ-DCW-65, MJ-DCW-92, MZBR1078, MZBR0276 |
| Hap_3 | 1 | P336744 |
| Hap_4 | 1 | P442979 |
| Hap_5 | 1 | P442980 |
| Hap_6 | 1 | P442988 |
| Hap_7 | 2 | P442989, CSJ-439 |
| Hap_8 | 10 | P442990, MJ-DCW-105, MJ-DCW-108, MJ-DCW-114, MJ-DCW-12, MJ-DCW-15, MJ-DCW-19, MJ-DCW-28, MZBR1164, MZBR1048 |
| Hap_9 | 9 | CSJ-1, CSJ-11, CSJ-134, CSJ-146, CSJ-177, CSJ-241, CSJ-296, CSJ-30, CSJ-99 |
| Hap_10 | 33 | CSJ-10, CSJ-100, CSJ-103, CSJ-112, CSJ-123, CSJ-150, CSJ-159, CSJ-161, CSJ-17, CSJ-198, CSJ-203, CSJ-215, CSJ-217, CSJ-23, CSJ-235, CSJ-264, CSJ-273, CSJ-298, CSJ-299, CSJ-316, CSJ-338, CSJ-349, CSJ-364, CSJ-394, CSJ-406, CSJ-445, CSJ-48, CSJ-71, CSJ-73, CSJ-90, CSJ-97, MJ-PRE-1, MZBR1064 |
| Hap_11 | 23 | CSJ-101, CSJ-133, CSJ-140, CSJ-163, CSJ-176, CSJ-185, CSJ-199, CSJ-221, CSJ-226, CSJ-263, CSJ-266, CSJ-286, CSJ-300, CSJ-304, CSJ-310, CSJ-452, CSJ-454, CSJ-456, CSJ-458, CSJ-82, CSJ-94, MJ-DCW-49, MJ-DCW-7 |
| Hap_12 | 1 | CSJ-102 |
| Hap_13 | 11 | CSJ-104, CSJ-125, CSJ-218, CSJ-240, CSJ-29, CSJ-33, CSJ-353, CSJ-450, MJ-DCW-35, MJ-DCW-74, MJ-DCW-84 |
| Hap_14 | 1 | CSJ-105 |
| Hap_15 | 26 | CSJ-107, CSJ-122, CSJ-131, CSJ-174, CSJ-175, CSJ-19, CSJ-202, CSJ-242, CSJ-243, CSJ-25, CSJ-277, CSJ-288, CSJ-31, CSJ-321, CSJ-403, CSJ-405, CSJ-407, CSJ-444, CSJ-59, CSJ-60, CSJ-65, CSJ-84, CSJ-85, CSJ-9, CSJ-91, MJ-DCW-39 |
| Hap_16 | 1 | CSJ-108 |
| Hap_17 | 1 | CSJ-111 |
| Hap_18 | 3 | CSJ-113, CSJ-312, CSJ-63 |
| Hap_19 | 5 | CSJ-114, CSJ-18, CSJ-200, CSJ-239, CSJ-75 |
| Hap_20 | 2 | CSJ-116, CSJ-436 |
| Hap_21 | 6 | CSJ-117, CSJ-250, CSJ-261, CSJ-424, MJ-DCW-59, MJ-DCW-60 |
| Hap_22 | 17 | CSJ-118, CSJ-178, CSJ-179, CSJ-197, CSJ-283, CSJ-319, CSJ-329, CSJ-35, CSJ-40, CSJ-411, CSJ-50, CSJ-72, CSJ-77, CSJ-96, MJ-DCW-11, MZBR1057, MZBR1034 |
| Hap_23 | 8 | CSJ-119, CSJ-147, CSJ-260, CSJ-269, CSJ-278, CSJ-294, CSJ-451, MJ-DCW-45 |
| Hap_24 | 17 | CSJ-12, CSJ-206, CSJ-22, CSJ-276, CSJ-281, CSJ-284, CSJ-313, CSJ-315, CSJ-333, CSJ-355, CSJ-393, CSJ-429, CSJ-431, CSJ-437, CSJ-438, CSJ-45, CSJ-457 |
| Hap_25 | 5 | CSJ-120, CSJ-196, CSJ-314, CSJ-415, CSJ-441 |
| Hap_26 | 1 | CSJ-121 |
| Hap_27 | 2 | CSJ-124, CSJ-325 |
| Hap_28 | 35 | CSJ-126, CSJ-130, CSJ-144, CSJ-149, CSJ-15, CSJ-158, CSJ-166, CSJ-169, CSJ-186, CSJ-2, CSJ-210, CSJ-225, CSJ-229, CSJ-262, CSJ-265, CSJ-274, CSJ-279, CSJ-295, CSJ-303, CSJ-311, CSJ-318, CSJ-335, CSJ-34, CSJ-346, CSJ-37, CSJ-395, CSJ-397, CSJ-398, CSJ-42, CSJ-51, CSJ-62, CSJ-67, CSJ-89, CSJ-95, MJ-DCW-53 |
| Hap_29 | 22 | CSJ-128, CSJ-132, CSJ-136, CSJ-167, CSJ-201, CSJ-212, CSJ-227, CSJ-228, CSJ-236, CSJ-237, CSJ-258, CSJ-293, CSJ-350, CSJ-43, CSJ-435, CSJ-443, CSJ-453, CSJ-47, CSJ-55, CSJ-92, MJ-DCW-41, MJ-PRE-72 |
| Hap_30 | 4 | CSJ-129, CSJ-348, CSJ-36, CSJ-400 |
| Hap_31 | 4 | CSJ-135, CSJ-170, CSJ-189, CSJ-259 |
| Hap_32 | 13 | CSJ-141, CSJ-164, CSJ-209, CSJ-290, CSJ-396, CSJ-399, CSJ-417, CSJ-46, CSJ-52, CSJ-57, CSJ-70, CSJ-87, MJ-DCW-81 |
| Hap_33 | 1 | CSJ-142 |
| Hap_34 | 1 | CSJ-151 |
| Hap_35 | 13 | CSJ-154, CSJ-211, CSJ-223, CSJ-231, CSJ-238, CSJ-297, CSJ-307, CSJ-359, CSJ-421, CSJ-423, CSJ-449, CSJ-69, CSJ-74 |
| Hap_36 | 2 | CSJ-156, CSJ-409 |
| Hap_37 | 1 | CSJ-16 |
| Hap_38 | 3 | CSJ-160, CSJ-280, MJ-DCW-70 |
| Hap_39 | 1 | CSJ-162 |
| Hap_40 | 1 | CSJ-168 |
| Hap_41 | 1 | CSJ-171 |
| Hap_42 | 13 | CSJ-180, CSJ-213, CSJ-24, CSJ-285, CSJ-358, CSJ-401, CSJ-413, CSJ-420, CSJ-49, CSJ-61, CSJ-80, CSJ-83, CSJ-98 |
| Hap_43 | 7 | CSJ-188, CSJ-208, CSJ-246, CSJ-282, CSJ-81, MJ-DCW-100, MJ-DCW-37 |
| Hap_44 | 1 | CSJ-194 |
| Hap_45 | 1 | CSJ-20 |
| Hap_46 | 6 | CSJ-205, CSJ-233, CSJ-268, CSJ-3, CSJ-308, CSJ-392 |
| Hap_47 | 2 | CSJ-207, CSJ-27 |
| Hap_48 | 2 | CSJ-219, CSJ-32 |
| Hap_49 | 1 | CSJ-232 |
| Hap_50 | 1 | CSJ-245 |
| Hap_51 | 2 | CSJ-247, MZBR0273 |
| Hap_52 | 1 | CSJ-248 |
| Hap_53 | 3 | CSJ-249, CSJ-367, CSJ-467 |
| Hap_54 | 11 | CSJ-251, CSJ-383, CSJ-464, MJ-DCW-61, MJ-DCW-75, MJ-PRE-33, MJ-PRE-35, MJ-PRE-47, MJ-PRE-48, MJ-PRE-50, MJ-PRE-52 |
| Hap_55 | 3 | CSJ-252, MJ-DCW-25, MJ-DCW-78 |
| Hap_56 | 5 | CSJ-253, MZBR1188, MZBR1179, MZBR1183, MZBR1182 |
| Hap_57 | 1 | CSJ-254 |
| Hap_58 | 3 | CSJ-255, MJ-DCW-23, MJ-DCW-87 |
| Hap_59 | 1 | CSJ-256 |
| Hap_60 | 1 | CSJ-257 |
| Hap_61 | 1 | CSJ-270 |
| Hap_62 | 1 | CSJ-292 |
| Hap_63 | 2 | CSJ-305, CSJ-53 |
| Hap_64 | 1 | CSJ-330 |
| Hap_65 | 2 | CSJ-332, CSJ-447 |
| Hap_66 | 1 | CSJ-340 |
| Hap_67 | 1 | CSJ-352 |
| Hap_68 | 4 | CSJ-357, CSJ-4, CSJ-54, CSJ-88 |
| Hap_69 | 2 | CSJ-361, CSJ-38 |
| Hap_70 | 3 | CSJ-362, MJ-DCW-76, MJ-DCW-79 |
| Hap_71 | 12 | CSJ-363, CSJ-368, CSJ-427, CSJ-460, MJ-DCW-109, MJ-DCW-112, MJ-DCW-146, MJ-DCW-3, MJ-DCW-34, MJ-DCW-54, MJ-DCW-67, MJ-PRE-24 |
| Hap_72 | 3 | CSJ-365, MJ-DCW-99, MJ-PRE-12 |
| Hap_73 | 5 | CSJ-370, CSJ-477, CSJ-479, MJ-DCW-103, MJ-DCW-13 |
| Hap_74 | 2 | CSJ-371, CSJ-375 |
| Hap_75 | 1 | CSJ-372 |
| Hap_76 | 4 | CSJ-373, CSJ-471, CSJ-478, MJ-DCW-58 |
| Hap_77 | 5 | CSJ-374, MG196302.1, MJ-DCW-30, MJ-DCW-50, MJ-PRE-21 |
| Hap_78 | 1 | CSJ-376 |
| Hap_79 | 6 | CSJ-377, CSJ-422, CSJ-465, MJ-DCW-107, MJ-DCW-77, MJ-PRE-18 |
| Hap_80 | 10 | CSJ-378, CSJ-425, CSJ-463, MJ-DCW-27, MJ-DCW-48, MJ-DCW-8, MJ-PRE-26, MJ-PRE-27, MJ-PRE-68, PA-DCW-7 |
| Hap_81 | 9 | CSJ-379, CSJ-380, CSJ-481, MJ-DCW-144, MJ-DCW-51, MJ-DCW-80, MJ-PRE-8, MJ-PRE-9, PA-DCW-5 |
| Hap_82 | 3 | CSJ-382, CSJ-474, MJ-DCW-43 |
| Hap_83 | 4 | CSJ-384, CSJ-462, CSJ-475, MJ-DCW-4 |
| Hap_84 | 2 | CSJ-385, MJ-PRE-20 |
| Hap_85 | 1 | CSJ-386 |
| Hap_86 | 1 | CSJ-388 |
| Hap_87 | 2 | CSJ-389, MJ-DCW-72 |
| Hap_88 | 1 | CSJ-39 |
| Hap_89 | 1 | CSJ-390 |
| Hap_90 | 1 | CSJ-391 |
| Hap_91 | 3 | CSJ-402, CSJ-412, CSJ-68 |
| Hap_92 | 1 | CSJ-416 |
| Hap_93 | 2 | CSJ-419, CSJ-44 |
| Hap_94 | 3 | CSJ-426, MJ-DCW-73, MJ-PRE-14 |
| Hap_95 | 1 | CSJ-428 |
| Hap_96 | 2 | CSJ-432, CSJ-56 |
| Hap_97 | 1 | CSJ-440 |
| Hap_98 | 1 | CSJ-446 |
| Hap_99 | 1 | CSJ-455 |
| Hap_100 | 1 | CSJ-461 |
| Hap_101 | 1 | CSJ-466 |
| Hap_102 | 1 | CSJ-468 |
| Hap_103 | 6 | CSJ-469, CSJ-476, MJ-DCW-115, MJ-DCW-2, MJ-DCW-62, MJ-DCW-9 |
| Hap_104 | 1 | CSJ-470 |
| Hap_105 | 2 | CSJ-472, MJ-PRE-6 |
| Hap_106 | 1 | CSJ-473 |
| Hap_107 | 1 | CSJ-480 |
| Hap_108 | 1 | CSJ-482 |
| Hap_109 | 1 | CSJ-483 |
| Hap_110 | 1 | CSJ-484 |
| Hap_111 | 1 | CSJ-5 |
| Hap_112 | 1 | CSJ-6 |
| Hap_113 | 1 | CSJ-64 |
| Hap_114 | 1 | CSJ-66 |
| Hap_115 | 1 | CSJ-76 |
| Hap_116 | 1 | CSJ-78 |
| Hap_117 | 1 | CSJ-8 |
| Hap_118 | 1 | KP306515.1 |
| Hap_119 | 2 | KT445979.1, MJ-PRE-73 |
| Hap_120 | 1 | MJ-DCW-1 |
| Hap_121 | 3 | MJ-DCW-10, MJ-DCW-64, MZBR1036 |
| Hap_122 | 1 | MJ-DCW-101 |
| Hap_123 | 2 | MJ-DCW-102, MJ-DCW-32 |
| Hap_124 | 1 | MJ-DCW-104 |
| Hap_125 | 3 | MJ-DCW-106, MJ-DCW-14, MJ-DCW-66 |
| Hap_126 | 4 | MJ-DCW-110, MJ-DCW-113, MJ-DCW-122, MJ-DCW-24 |
| Hap_127 | 1 | MJ-DCW-111 |
| Hap_128 | 1 | MJ-DCW-117 |
| Hap_129 | 2 | MJ-DCW-121, MJ-DCW-94 |
| Hap_130 | 1 | MJ-DCW-127 |
| Hap_131 | 1 | MJ-DCW-142 |
| Hap_132 | 1 | MJ-DCW-16 |
| Hap_133 | 1 | MJ-DCW-17 |
| Hap_134 | 2 | MJ-DCW-18, MJ-DCW-82 |
| Hap_135 | 1 | MJ-DCW-20 |
| Hap_136 | 2 | MJ-DCW-21, MJ-DCW-88 |
| Hap_137 | 1 | MJ-DCW-22 |
| Hap_138 | 3 | MJ-DCW-26, MJ-DCW-85, MJ-DCW-91 |
| Hap_139 | 1 | MJ-DCW-29 |
| Hap_140 | 2 | MJ-DCW-31, MJ-PRE-3 |
| Hap_141 | 2 | MJ-DCW-33, MJ-DCW-36 |
| Hap_142 | 1 | MJ-DCW-38 |
| Hap_143 | 1 | MJ-DCW-40 |
| Hap_144 | 1 | MJ-DCW-42 |
| Hap_145 | 1 | MJ-DCW-44 |
| Hap_146 | 1 | MJ-DCW-47 |
| Hap_147 | 1 | MJ-DCW-5 |
| Hap_148 | 1 | MJ-DCW-52 |
| Hap_149 | 1 | MJ-DCW-57 |
| Hap_150 | 2 | MJ-DCW-6, MJ-DCW-93 |
| Hap_151 | 1 | MJ-DCW-63 |
| Hap_152 | 1 | MJ-DCW-69 |
| Hap_153 | 1 | MJ-DCW-71 |
| Hap_154 | 1 | MJ-DCW-86 |
| Hap_155 | 2 | MJ-DCW-90, MZBR1083 |
| Hap_156 | 1 | MJ-DCW-95 |
| Hap_157 | 1 | MJ-DCW-96 |
| Hap_158 | 1 | MJ-DCW-97 |
| Hap_159 | 1 | MJ-DCW-98 |
| Hap_160 | 1 | MJ-PRE-1 |
| Hap_161 | 1 | MJ-PRE-10 |
| Hap_162 | 1 | MJ-PRE-11 |
| Hap_163 | 1 | MJ-PRE-13 |
| Hap_164 | 32 | MJ-PRE-15, MJ-PRE-16, MJ-PRE-17, MJ-PRE-28, MJ-PRE-29, MJ-PRE-30, MJ-PRE-31, MJ-PRE-32, MJ-PRE-34, MJ-PRE-36, MJ-PRE-37, MJ-PRE-38, MJ-PRE-39, MJ-PRE-40, MJ-PRE-41, MJ-PRE-42, MJ-PRE-43, MJ-PRE-44, MJ-PRE-45, MJ-PRE-46, MJ-PRE-49, MJ-PRE-54, MJ-PRE-55, MJ-PRE-56, MJ-PRE-57, MJ-PRE-58, MJ-PRE-59, MJ-PRE-61, MJ-PRE-62, MJ-PRE-63, MJ-PRE-65, MJ-PRE-66 |
| Hap_165 | 1 | MJ-PRE-19 |
| Hap_166 | 1 | MJ-PRE-22 |
| Hap_167 | 1 | MJ-PRE-23 |
| Hap_168 | 1 | MJ-PRE-25 |
| Hap_169 | 1 | MJ-PRE-4 |
| Hap_170 | 1 | MJ-PRE-5 |
| Hap_171 | 4 | MJ-PRE-51, MJ-PRE-53, MJ-PRE-60, MJ-PRE-64 |
| Hap_172 | 2 | MJ-PRE-67, MJ-PRE-70 |
| Hap_173 | 1 | MJ-PRE-69 |
| Hap_174 | 1 | MJ-PRE-7 |
| Hap_175 | 1 | MJ-PRE-71 |
| Hap_176 | 1 | PA-DCW-8 |
| Hap_177 | 2 | MZBR1190, MZBR1180 |
| Hap_178 | 4 | MZBR1189, MZBR1076, MZBR1184, MZBR1181 |
| Hap_179 | 5 | MZBR1055, MZBR1157, MZBR1081, MZBR1086, MZBR1050 |
| Hap_180 | 3 | MZBR1087, MZBR1162, MZBR1052 |
| Hap_181 | 1 | MZBR1043 |
| Hap_182 | 1 | MZBR0274 |
| Hap_183 | 1 | MZBR1074 |
| Hap_184 | 1 | MZBR0275 |
| Hap_185 | 1 | MZBR1040 |
| Hap_186 | 2 | MZBR1044, MZBR1163 |
| Hap_187 | 3 | MZBR1166, MZBR1178, MZBR1177 |
| Hap_188 | 1 | MZBR1042 |
| Hap_189 | 1 | MZBR1071 |
| Hap_190 | 1 | MZBR1030 |
| Hap_191 | 1 | MZBR1038 |
| Hap_192 | 1 | MZBR1072 |
| Hap_193 | 2 | MZBR1046, MZBR1077 |
| Hap_194 | 1 | MZBR1054 |
| Hap_195 | 2 | MZBR1053, MZBR1070 |
| Hap_196 | 1 | MZBR1073 |
| Hap_197 | 1 | MZBR1084 |
| Hap_198 | 1 | MZBR1165 |
| Hap_199 | 1 | MZBR1082 |
| Hap_200 | 1 | MZBR1063 |
| Hap_201 | 1 | MZBR1069 |
| Hap_202 | 1 | MZBR1085 |

**Supplementary Table 18.** **Comparison of heterozygosity among endangered species.**

| Species | Samples | SNPs | Heterozygosity | Reference genome size/Gb | Conservation status | Reference |
| --- | --- | --- | --- | --- | --- | --- |
| CMA:  Brown eared pheasant  (*C. mantchuricum*) | 37 | 49,701 | 0.000095 | 1.0 | VU | ^26^ |
| ANI: Chinese alligator (*A. sinensis*) | 23 | 312,525 | 0.00012 | 2.3 | CR | ^27^ |
| LVE: Baiji  (*L. vexillifer*) | / | / | 0.00026 | 2.4 | CR | ^28^ |
| RRO: Golden snub-nosed monkey (*R. roxellana*) | 27 | / | 0.000405 | 2.9 | EN | ^29^ |
| PTI: Mixed population of South China tiger (*P. tigris amoyensis*) and Siberian tiger (*P. tigris altaica*) | 28 | 6,289,055 | 0.0006695 | 2.5 | CR | ^16^ |
| AME: Giant panda (*A. melanoleuca*) | 25 | 3,289,986 | 0.001198 | 2.4 | VU | ^11^ |
| GGG: Western lowland gorilla  (*G. gorilla gorilla*) | 27 | 1,755,172 | 0.001438 | 3.1 | CR | ^30^ |
| MPE: Chinese pangolin  (*M. pentadactyla*) | 37 | 35,023,399 | 0.00182 | 2.4 | CR | ^5^ |
| ULI: Island fox  (*U. littoralis*) | / | / | 0.00197 | 2.4 | NT | ^31^ |
| PPP: African leopard (*P. pardus pardus*) | 53 | / | 0.001979 | 2.6 | NT | ^17^ |
| EFP: Przewalski's horse  (*E. ferus przewalskii*) | / | / | 0.00363 | 2.4 | EN | ^32^ |

**Supplementary Table 19.** **The average number (N_ROH_) of different lengths of ROH per individual in pangolin populations.**

|  | >100 kb | 100 kb<X<1 Mb | >1 Mb |
| --- | --- | --- | --- |
| MJ1 | 1188.86 ± 218.74 | 1153.86 ± 207.54 | 35.00 ± 30.04 |
| MJ2 | 770.77 ± 74.69 | 735.45 ± 69.50 | 35.32 ± 20.19 |
| MJ3 | 920.35 ± 499.93 | 867.94 ± 471.32 | 52.41 ± 49.27 |
| MJ | 917.65 ± 488.59 | 866.19 ± 460.79 | 51.46 ± 48.32 |

**Supplementary Table 20.** **The mean cumulative fraction (F_ROH_) of different lengths of ROH per individual in pangolin populations**.

|  | >100 kb | 100 kb<X<1 Mb | >1 Mb |
| --- | --- | --- | --- |
| MJ1 | 13.63 ± 1.62 % | 11.44 ± 1.13 % | 2.19 ± 0.76 % |
| MJ2 | 10.16 ± 0.52% | 7.52 ± 0.20 % | 2.64 ± 0.37 % |
| MJ3 | 13.29 ± 0.40 % | 8.74 ± 0.24 % | 4.55 ± 0.21 % |
| MJ | 13.16 ± 0.38 % | 8.73 ± 0.22 % | 4.43 ± 0.21 % |

**Supplementary Table 21. Summary of three type mutational loads in pangolin populations.**

| Type | | MJ1 | MJ2 | MJ3 |
| --- | --- | --- | --- | --- |
| Missense | Heterozygous | 10678.14 ± 387.81 | 12306.27 ± 1919.58 | 10732.57 ± 1795.35 |
|  | Homozygous | 11544.57 ± 302.91 | 10705.18 ± 185.60 | 11128.89 ± 702.07 |
|  | Ratio/% | 68.37 ± 1.28 | 63.65 ± 3.08 | 67.56 ± 4.87 |
| LoF | Heterozygous | 416.43 ± 32.20 | 467.27 ± 152.63 | 379.28 ± 76.19 |
|  | Homozygous | 331.57 ± 21.10 | 298.50 ± 11.22 | 309.89 ± 25.31 |
|  | Ratio/% | 61.39 ± 2.81 | 56.78 ± 5.28 | 62.22 ± 5.98 |

**Supplementary Figures**

**

**

**Supplementary Figure 1 | Detailed information of pangolin samples used in this study, including published (in light brown) and newly sequenced (in dark brown).**

**
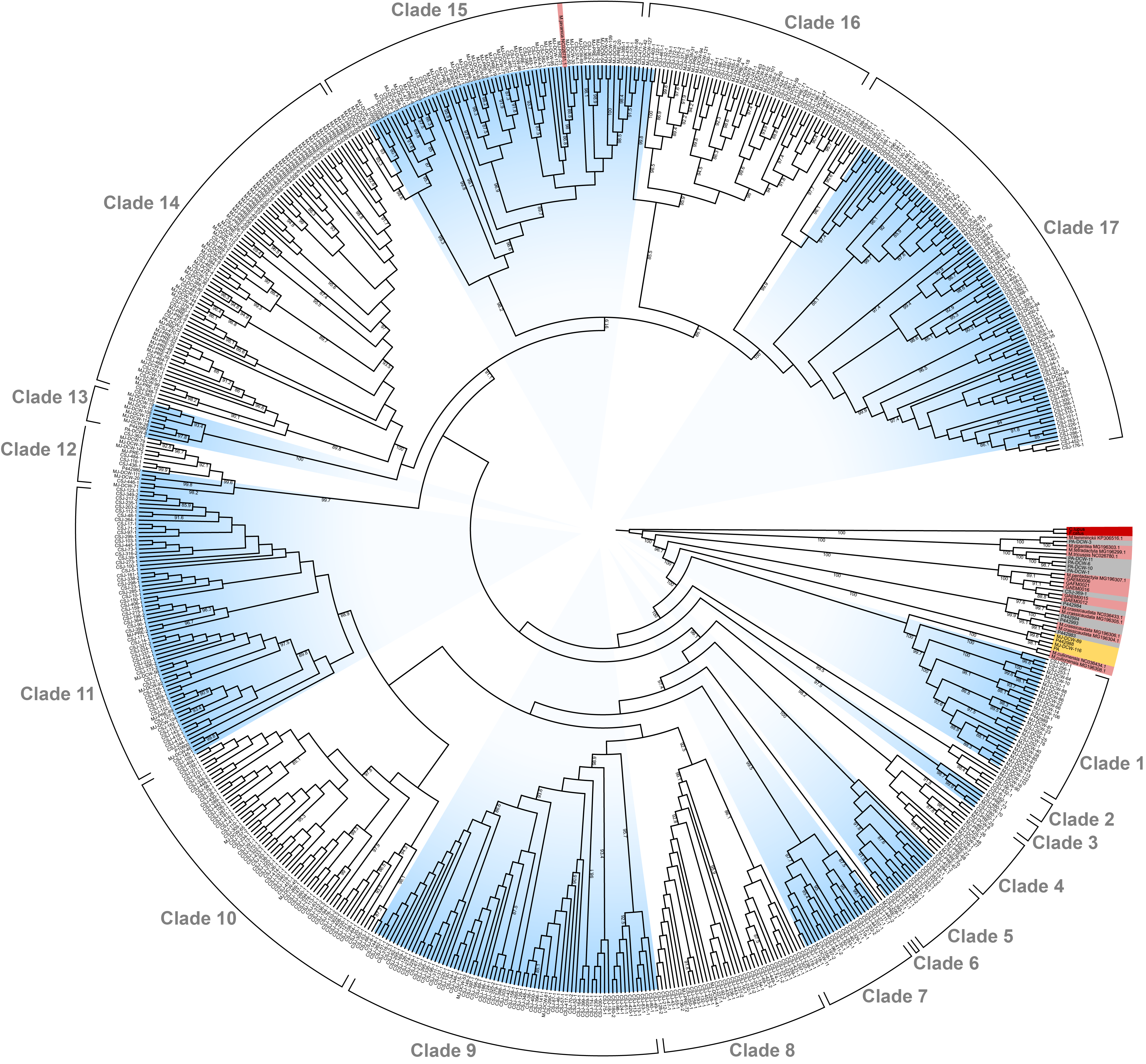
**

**Supplementary Figure 2 | Maximum likelihood (ML) tree was constructed using the mitochondrial genes of 687 pangolins, including 594 pangolins sequenced in this study and 93 pangolins that have been published.** Colored labels represent individuals of different population: Blue and white represent the 17 Malayan pangolin clades roughly divided in this study, red represents the reference mitochondrial genome and outgroups, and yellow represents the four focused individuals (MJ-DCW-89, MJ-DCW-116, P442988, PA).


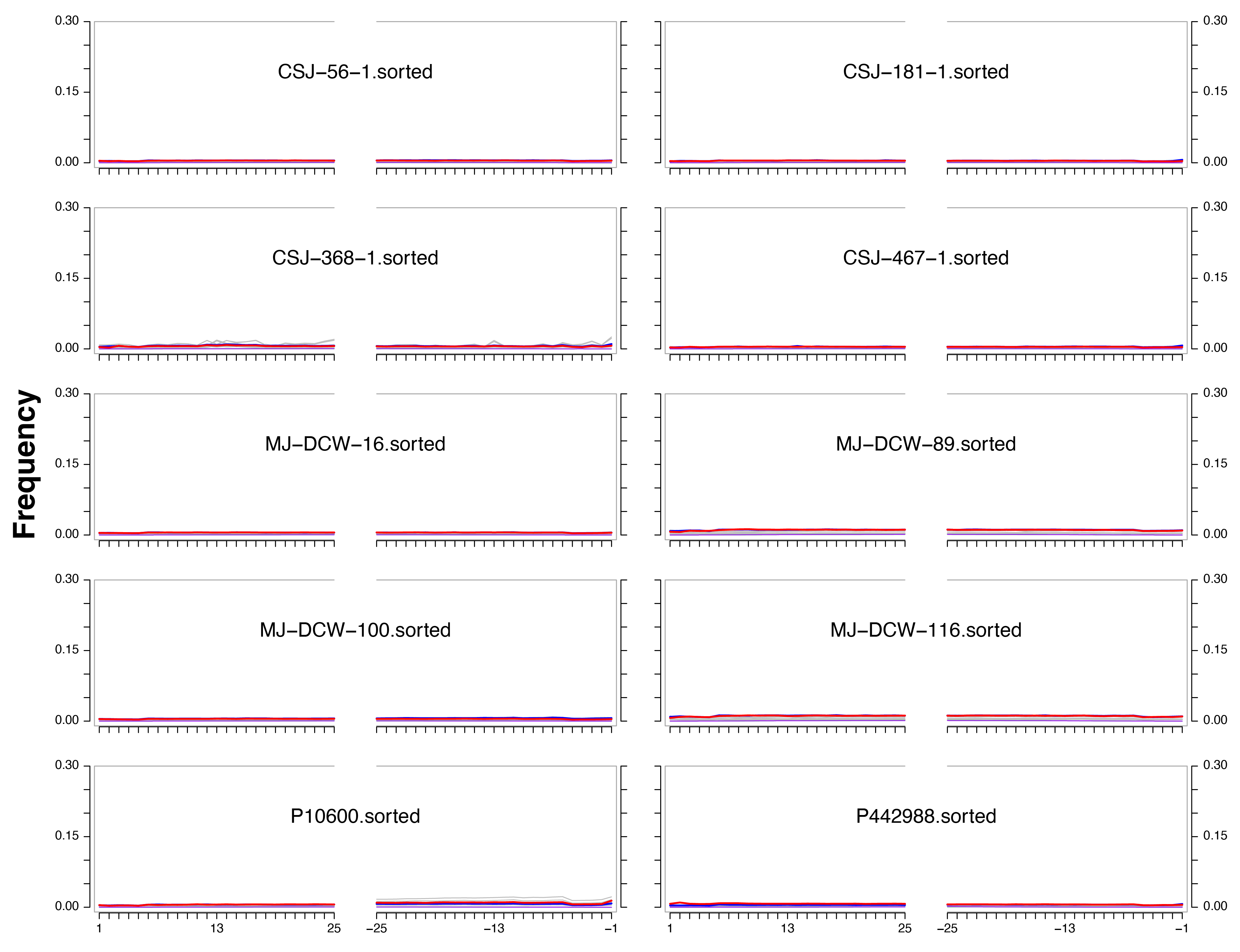


**Supplementary Figure 3 | Deamination plot of pangolin individuals showing frequency of C to T (red) and G to A (blue) substitutions in sequencing reads aligned to the reference.** The plots are the positions specific substitutions from the 5’ (left) and the 3’ end (right). Purple: Insertions relative to the reference. Green: Deletions relative to the reference. Orange: Soft-clipped bases. Grey: All other substitutions.

**
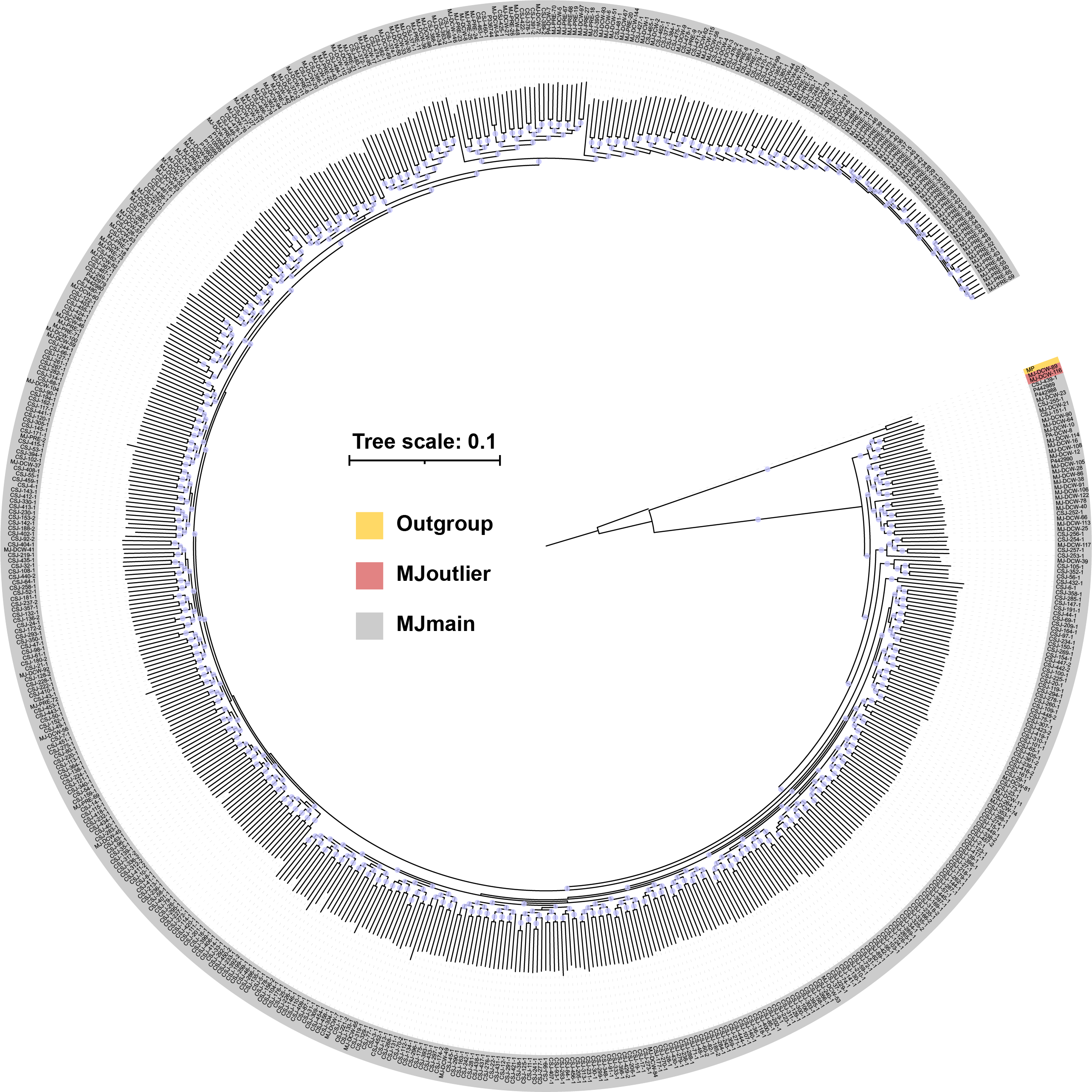
**

**Supplementary Figure 4 | Maximum likelihood (ML) phylogenetic trees based on autosomal SNPs of 598 pangolin genomes for distinguishing MJoutlier from MJmain.**

**
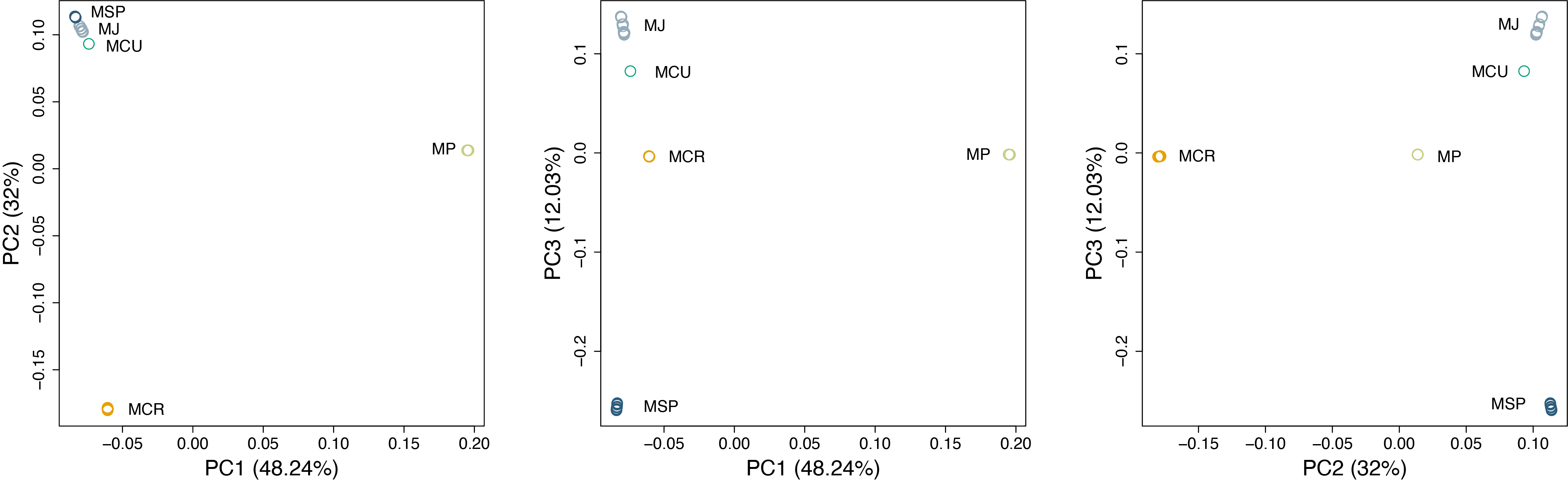
**

**Supplementary Figure 5 | PCA results of five Asian pangolin species.**


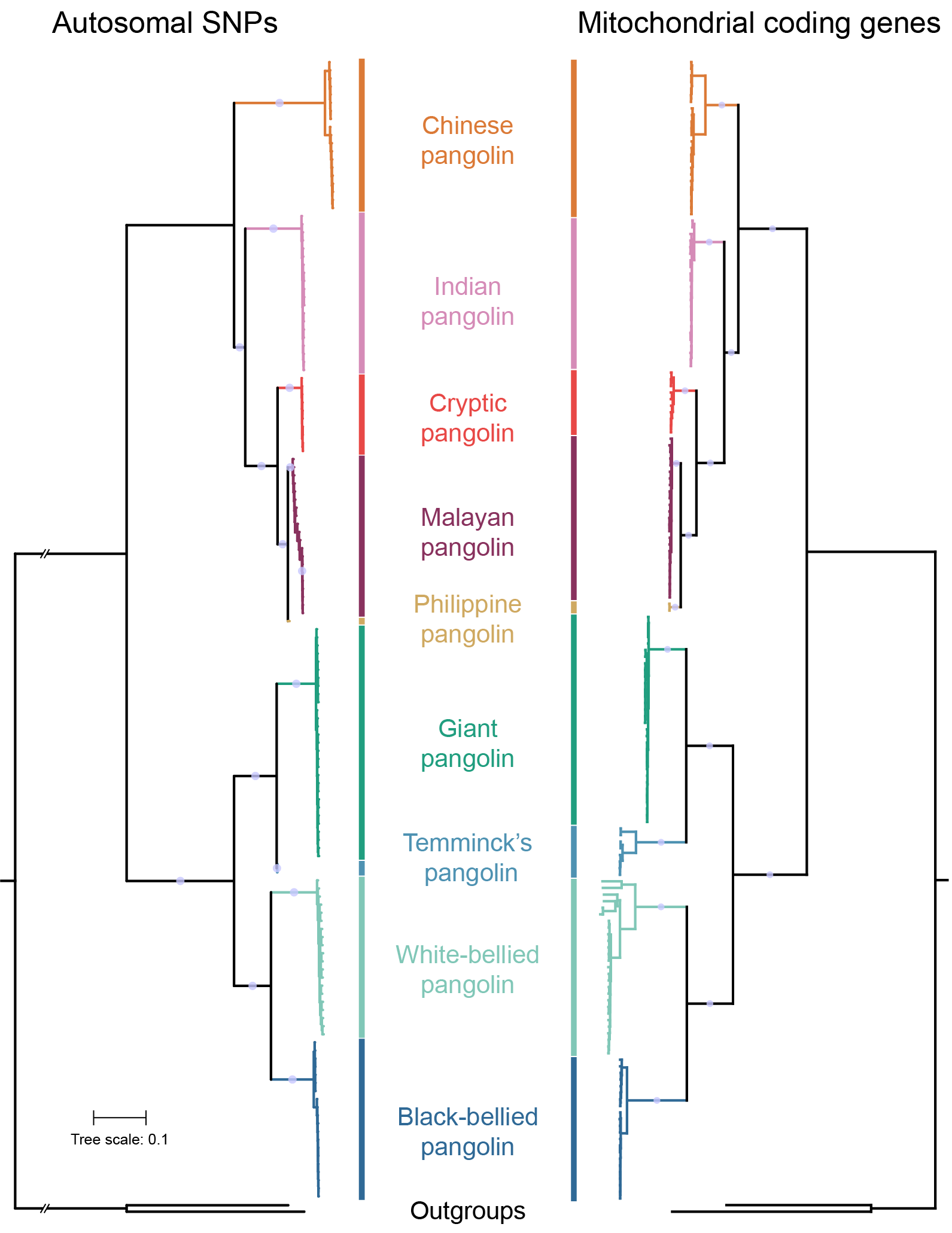


**Supplementary Figure 6 | Maximum likelihood (ML) phylogenetic tree of nine pangolin species (N=141) based on 13 mitochondrial-encoded genes (right) and autosomal SNPs (left).** The domestic dog and domestic cat from the Order Carnivora were regarded as outgroups.


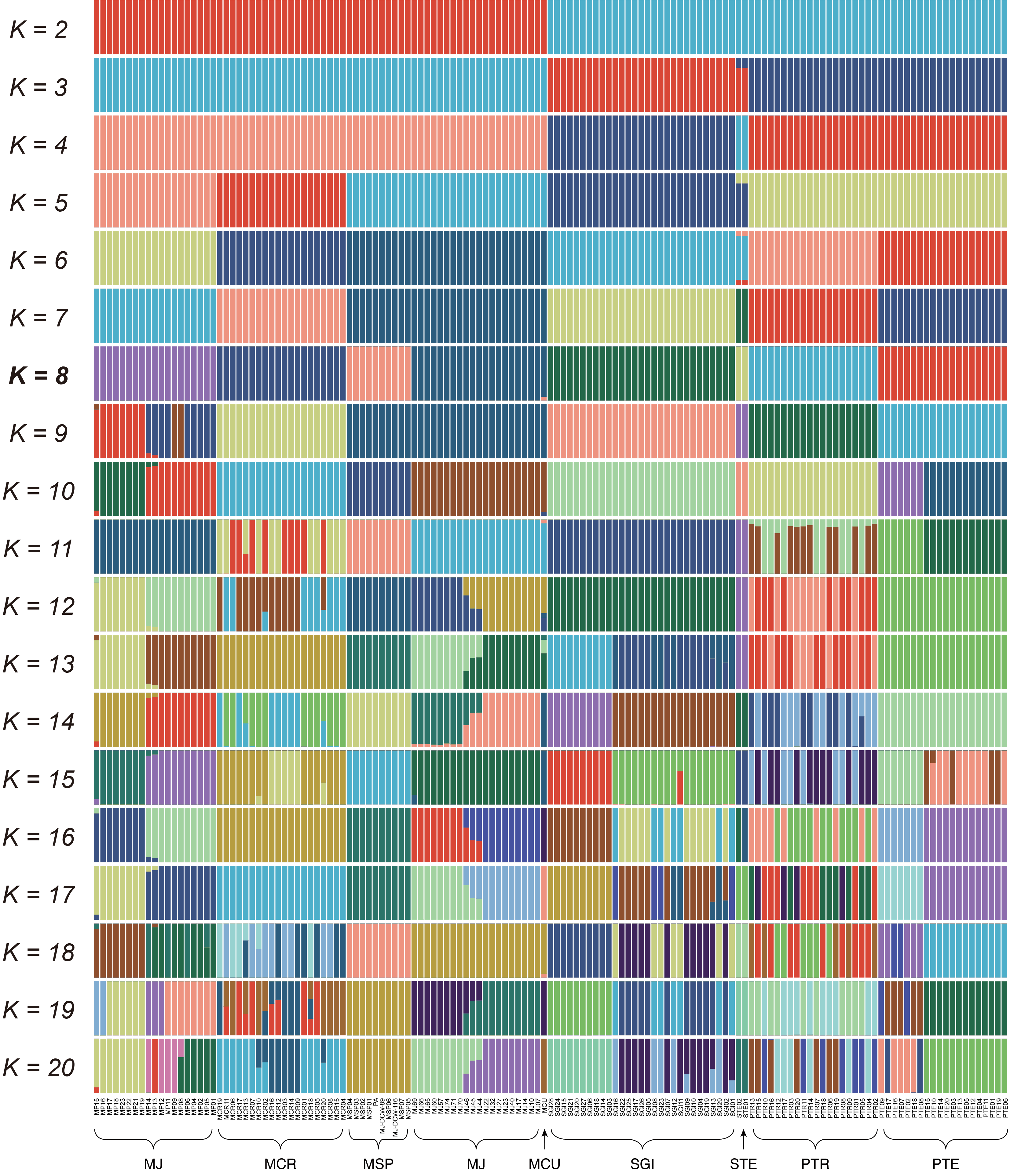


**Supplementary Figure 7 | Admixture analysis of nine pangolin species (N=141) datasets with *K*=2~20.**

**
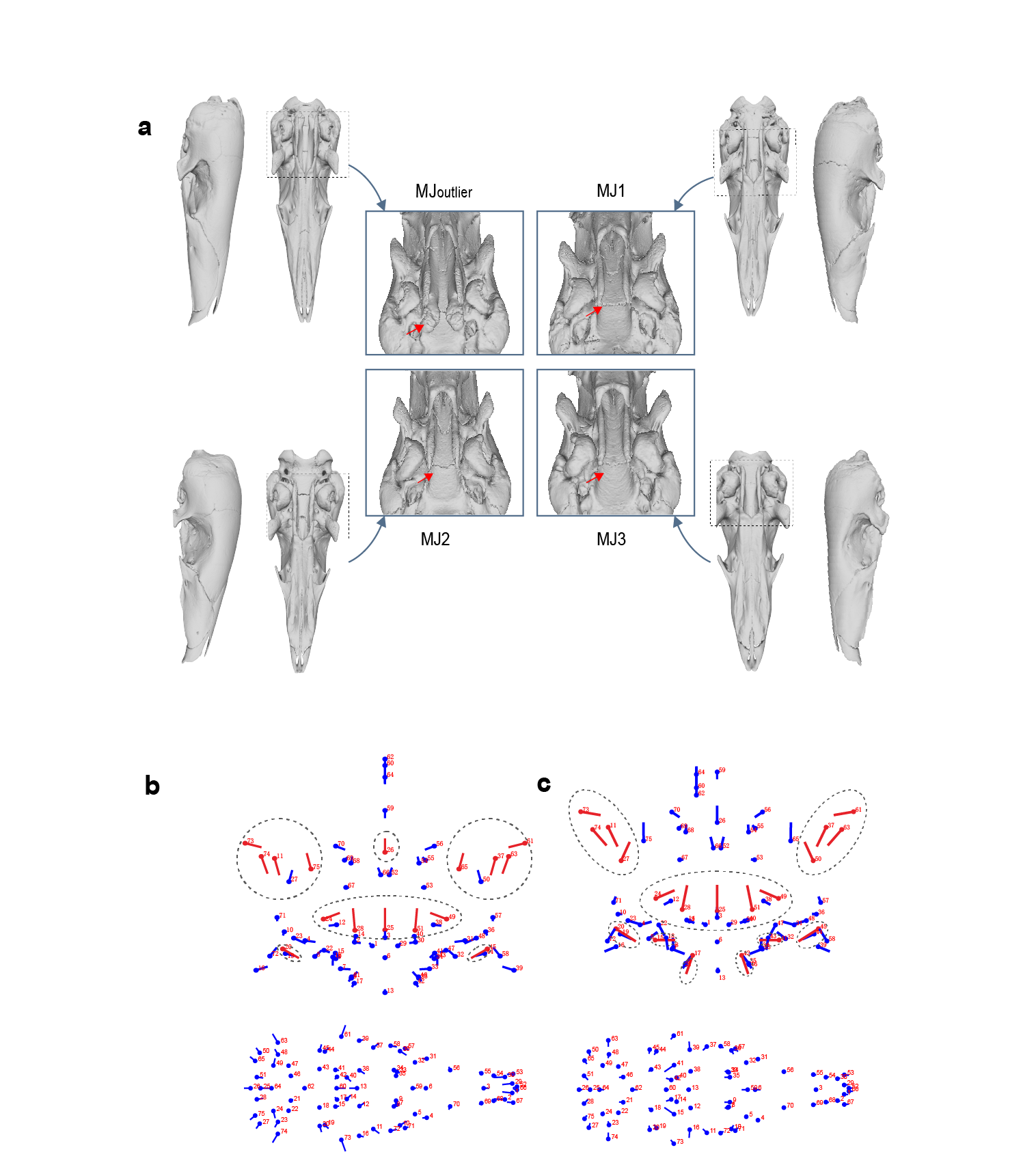
**

**Supplementary Figure 8 | Morphology and morphometric analysis of pangolin skulls.** **a** The skull of MJ_outlier_ compared to skulls from Malayan pangolin subpopulations; The region that MJ_outlier_ differ from the MJ_main_ is indicated by red arrows. **b** The upper figure shows the shape changes at landmarks on the MJ_outlier_ skull (red lollipops in dashed circles) comparing to the MJ_main_ (blue lollipops). The lower figure shows the overall shape variation of the MJ_main_ skulls. **c** The upper figure shows the shape changes at landmarks on the MJ_outlier_ skull (red lollipops in dashed circles) comparing to four species of Asian pangolin including the MJ_main_. The lower figure shows overall shape variation in the four Asian pangolin species including the MJ_main_.

**
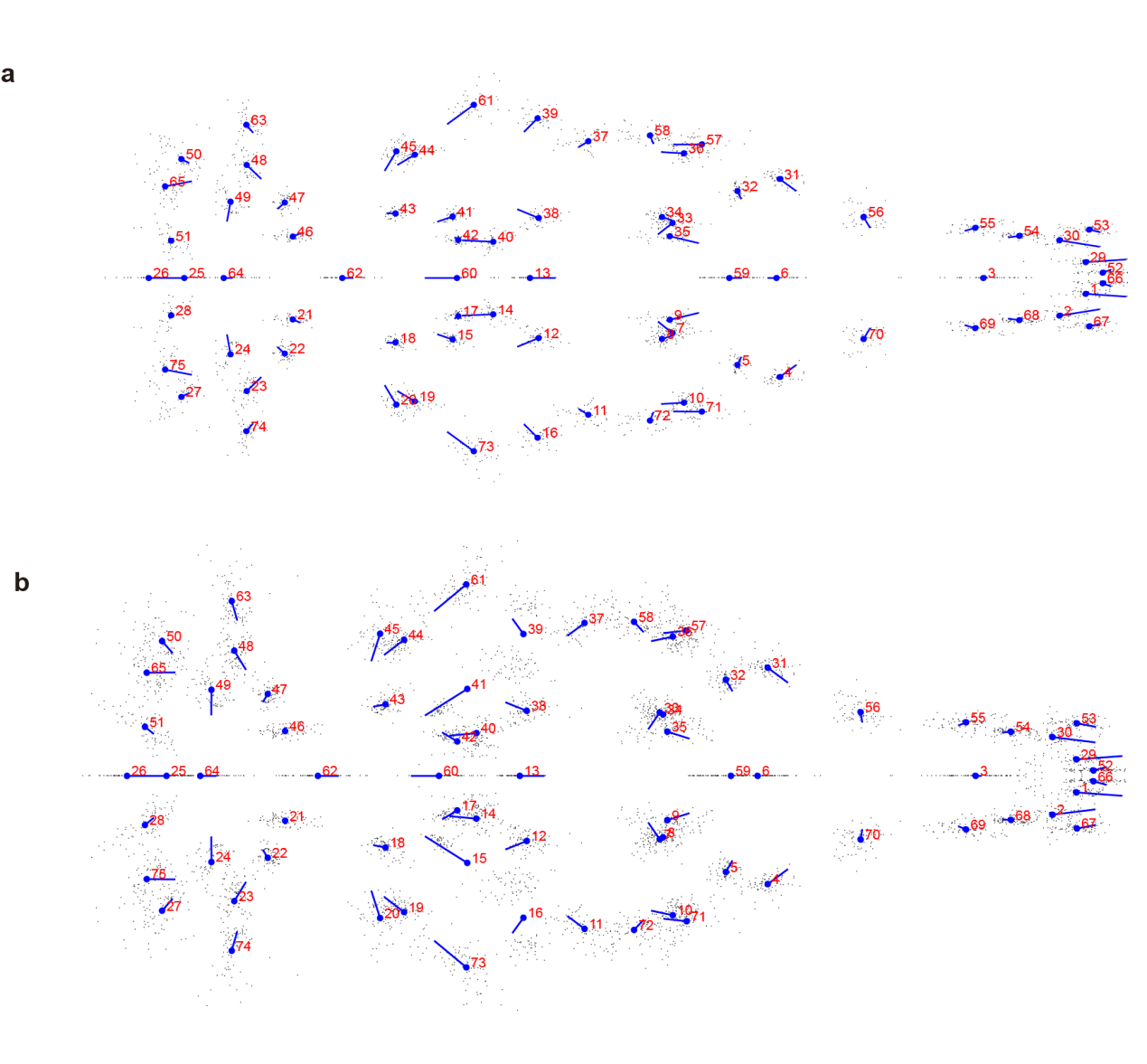
**

**Supplementary Figure 9 | Individual deformation of MJ_outlier_ and four Asian pangolin species (including Malayan pangolin).** The plot of the surface points of each individual after the Procrustes fit is overlaid with the individual deformation plot of MJ_outlier_. **a,** The morphogram representing MJ_outlier_ overlaps with the MJ_main_. **b,** The morphogram representing MJ_outlier_ with the four Asian pangolin group. The lollipop graph shows the shifts of landmark positions with straight lines. Each blue line starts with a mean shape dot at the location of the landmark in the starting shape. The length and direction of this line indicate the shifting change of the MJ_outlier_ landmark.


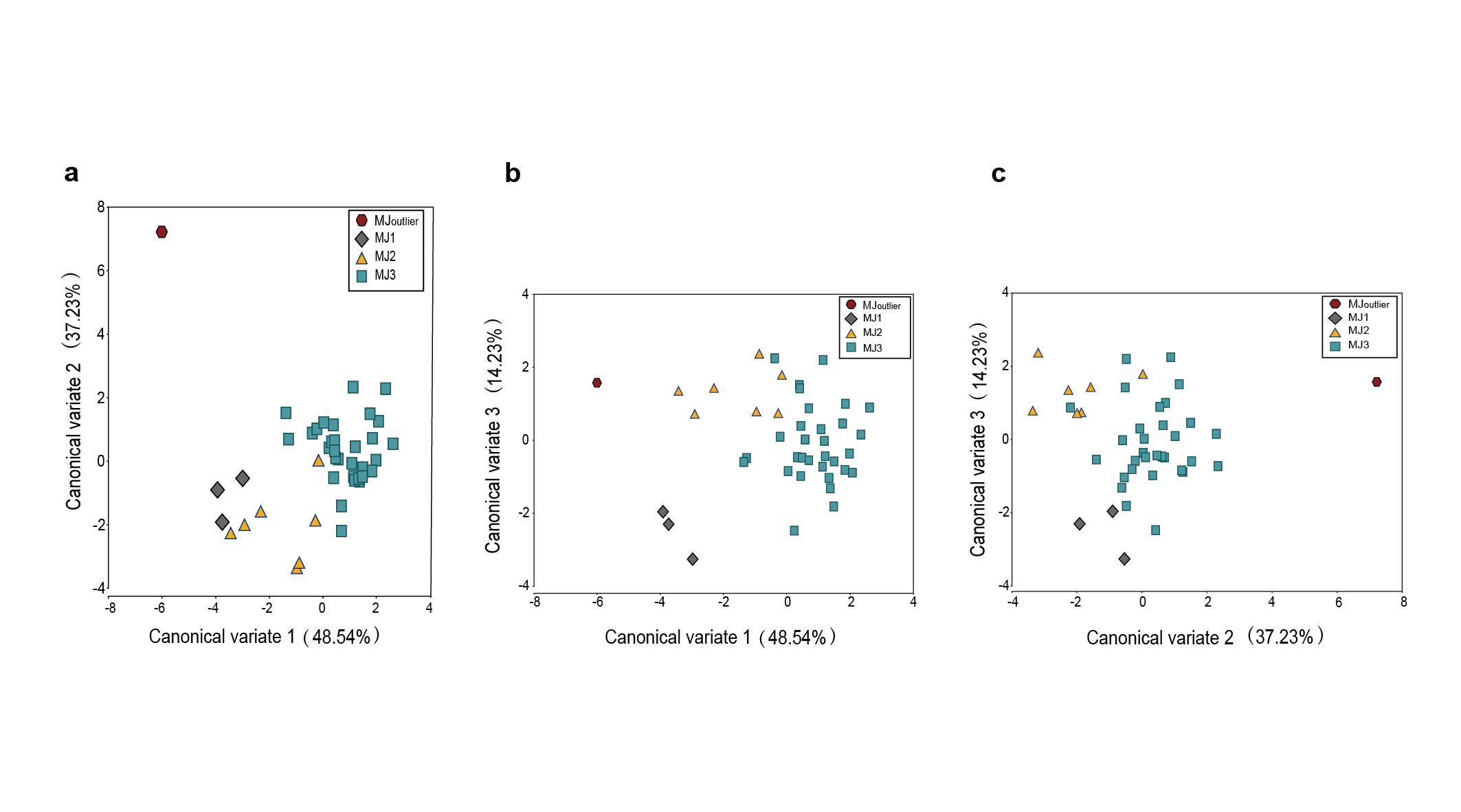


**Supplementary Figure 10 | Scatter plot showing the variation in shape of skulls of MJ_outlier_ and the MJ_main_ along the first three canonical variate (CV1, CV2 andCV3) axes.**

**
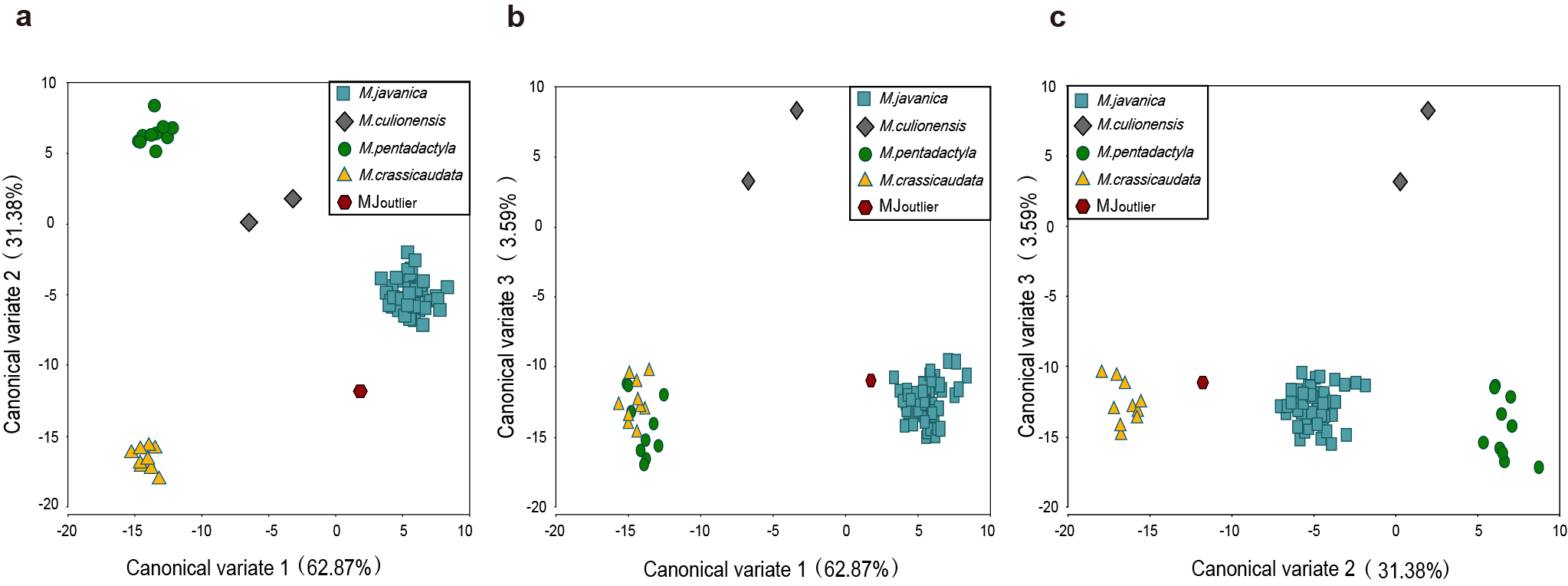
**

**Supplementary Figure 11 | Scatter plot showing the variation in shape of skulls of MJ_outlier_ and four Asian pangolin species (including** **the MJ_main_) along the first three canonical variate (CV1, CV2 and CV3).**


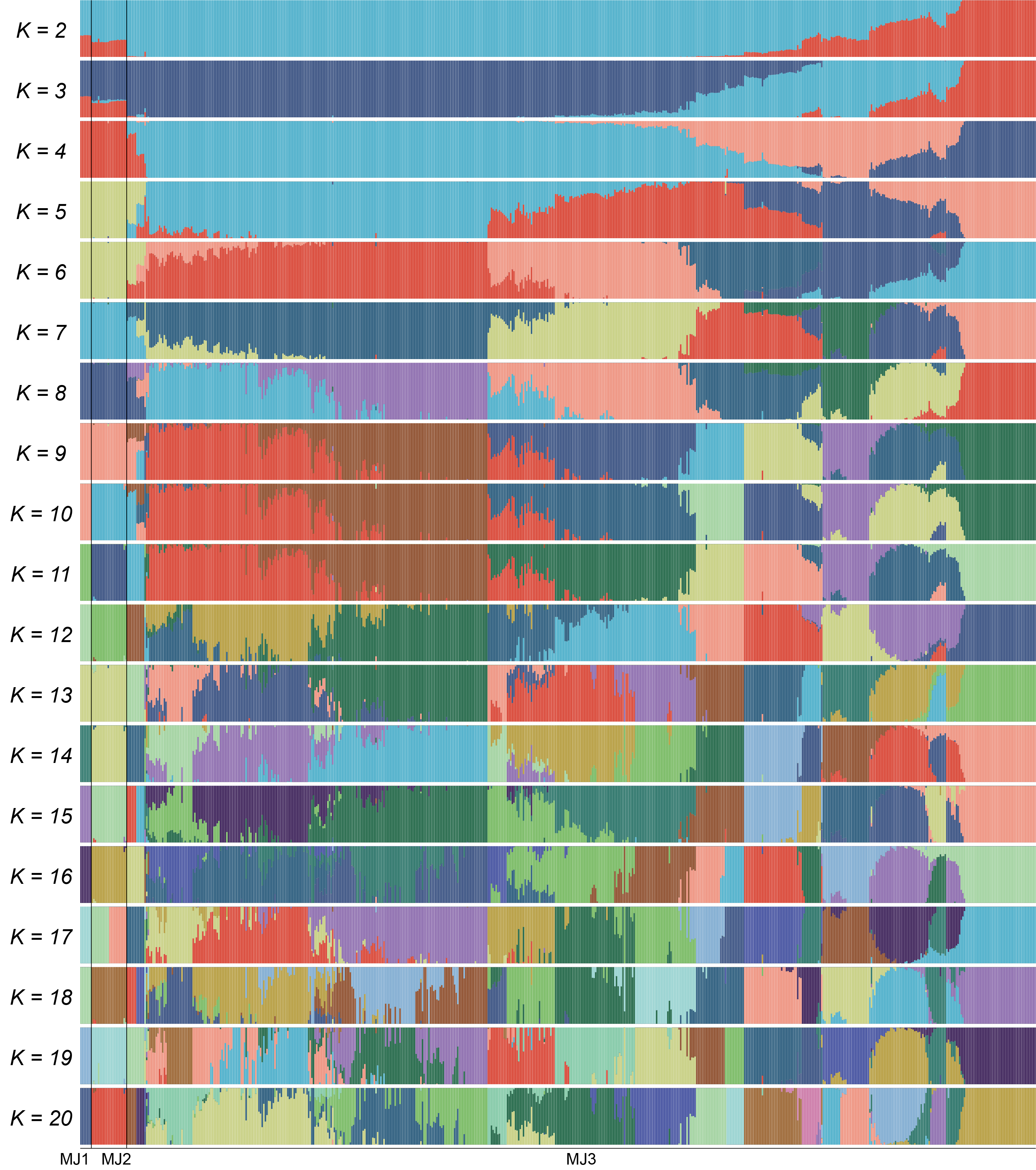


**Supplementary Figure 12 |** **Admixture analysis of 596 Malayan pangolins (MJmain) datasets with the *K*= 2 ~ 20.**


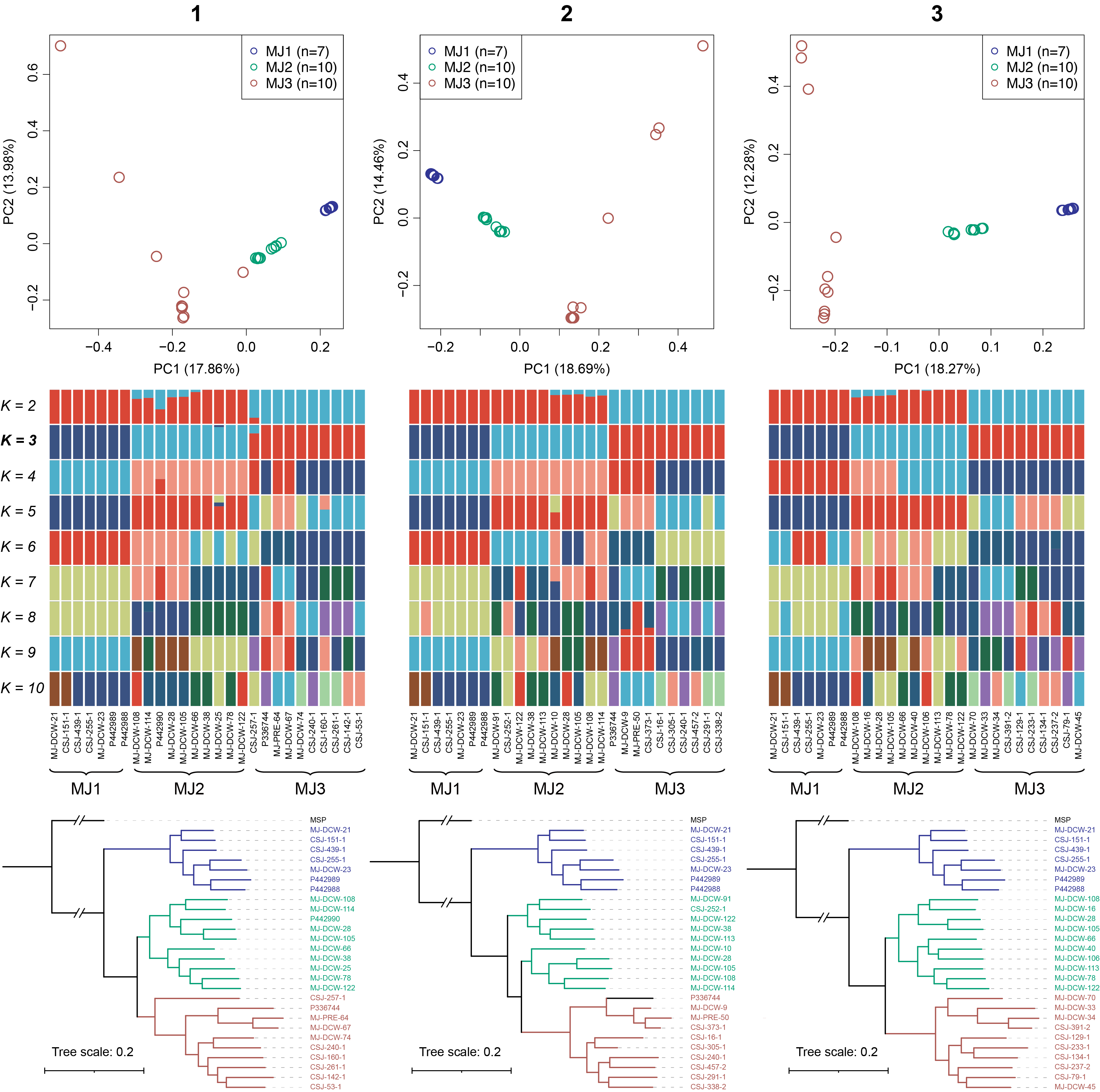


**Supplementary Figure 13 |** **PCA, admixture clustering, and phylogeny analyses of 27 individuals randomly selected from three Malayan pangolin populations.** This was used to eliminate the effect of population size differences on genetic structure, and the random sample was repeated three times.


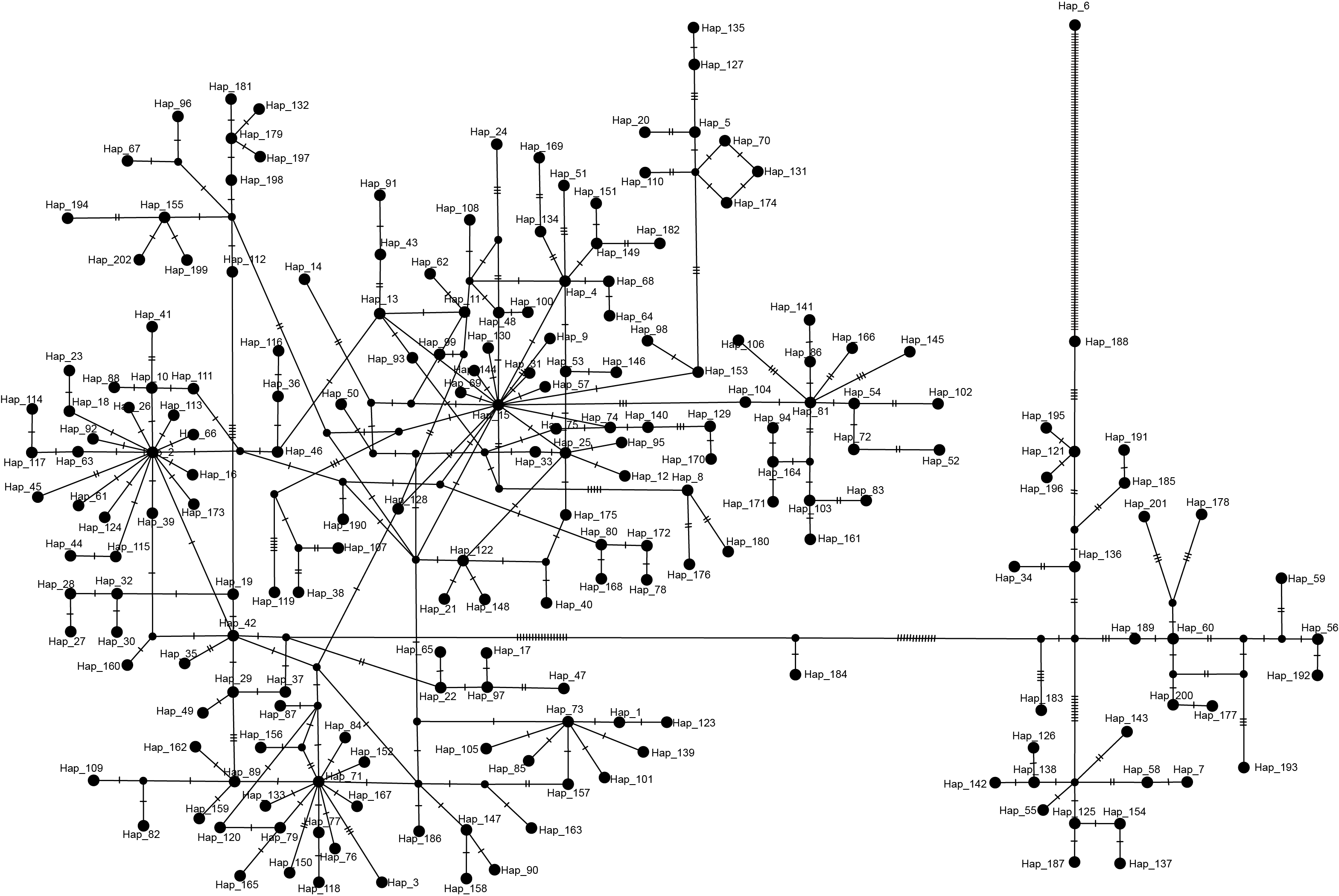


**Supplementary Figure 14 | Specific haplotype markers for haplotype network (Fig. 3C).**

**
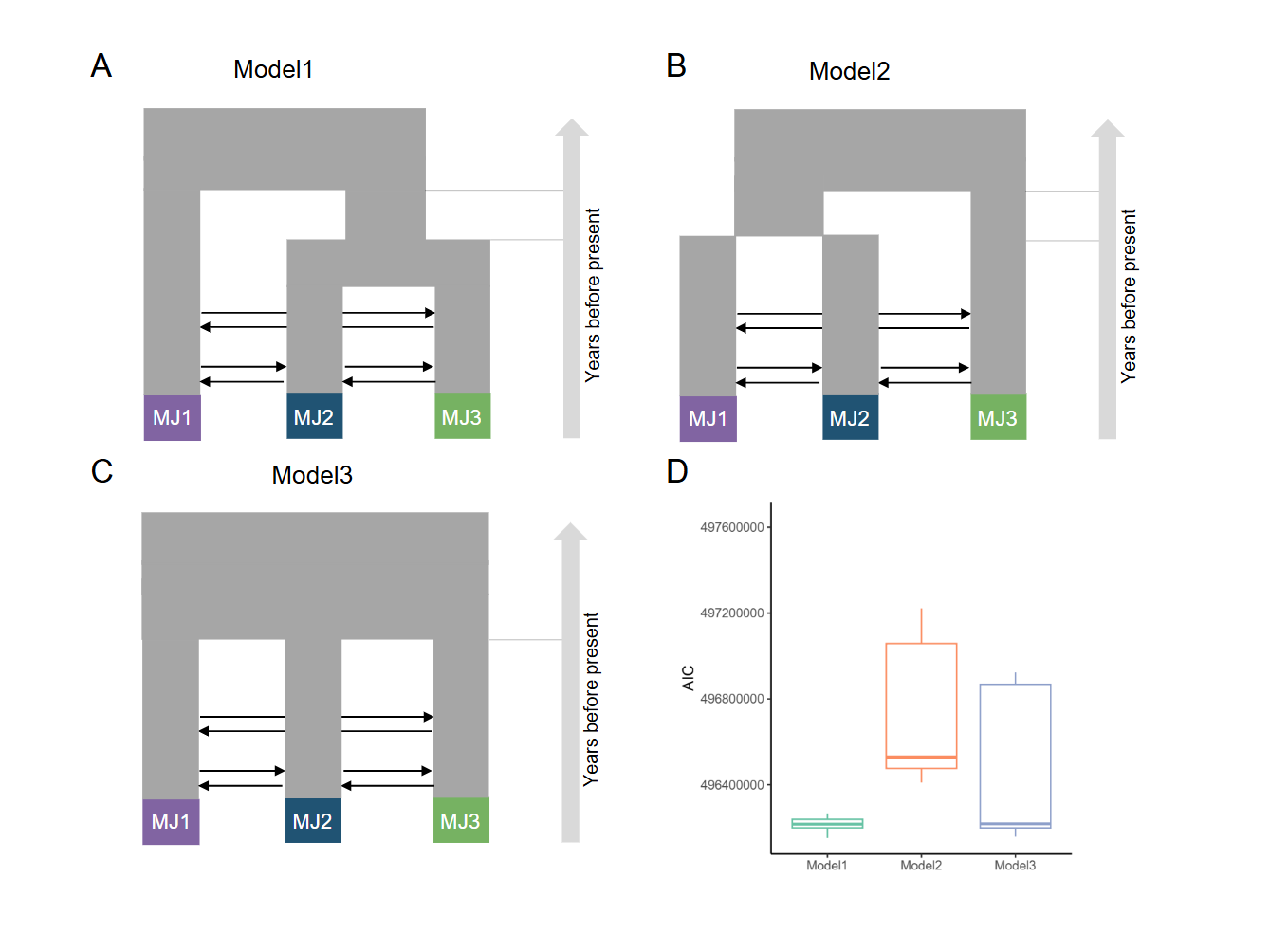
**

**Supplementary Figure 15 | Demographic model inferred from fastsimcoal2**


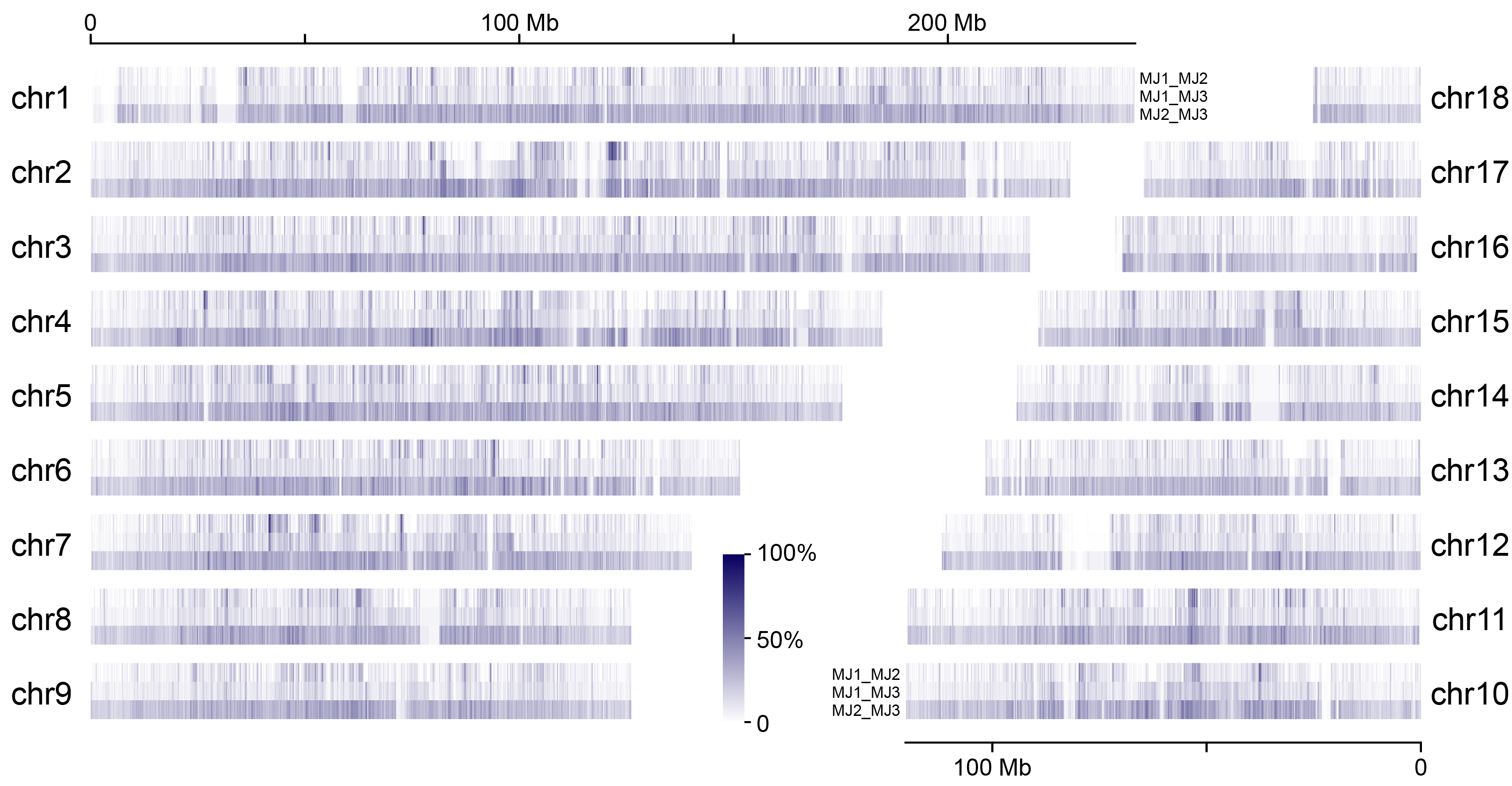


**Supplementary Figure 16 | Distribution of IBD on chromosomes. The number of IBD occurrences was counted in a 50 Kb window and normalized by the total number of individual pairs.**


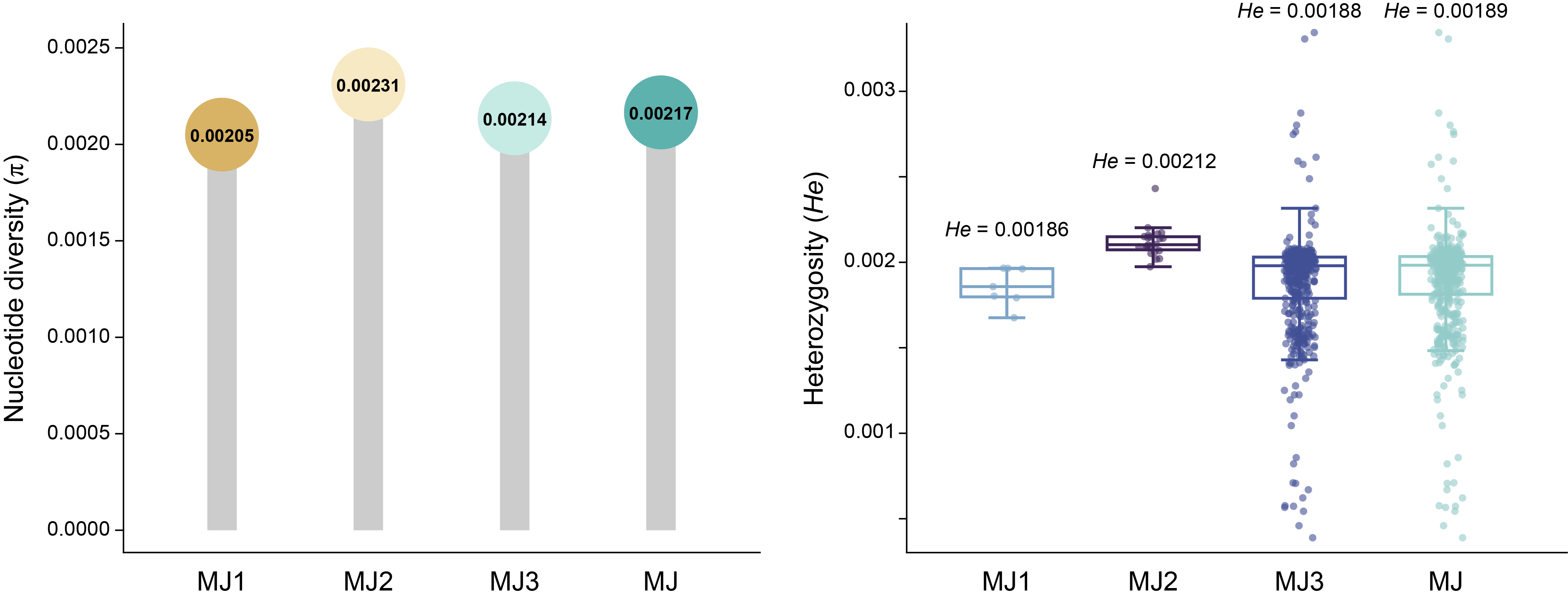


**Supplementary Figure 17 | Genetic diversity of each pangolin population. a,** The average nucleotide diversity (π) in non-overlapping 50kb windows of three Malayan pangolins populations and the total Malayan pangolins (MJ). **b,** Heterozygosity per individual in three Malayan pangolins populations and the total Malayan pangolins (MJ).


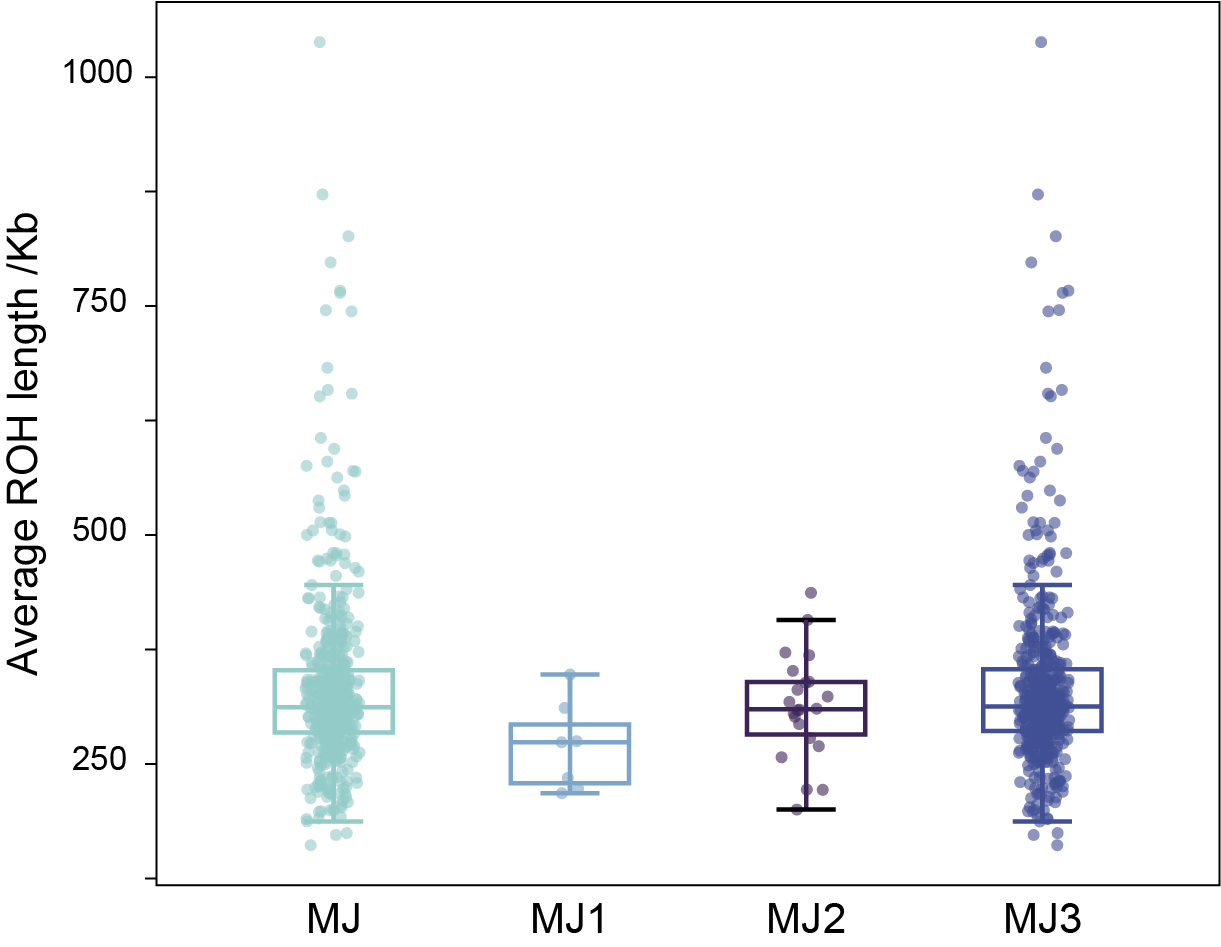

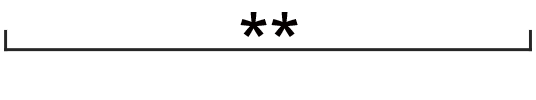


**Supplementary Figure 18 |** **The average length of run of homozygosity (ROH) in the genomes of each population.**


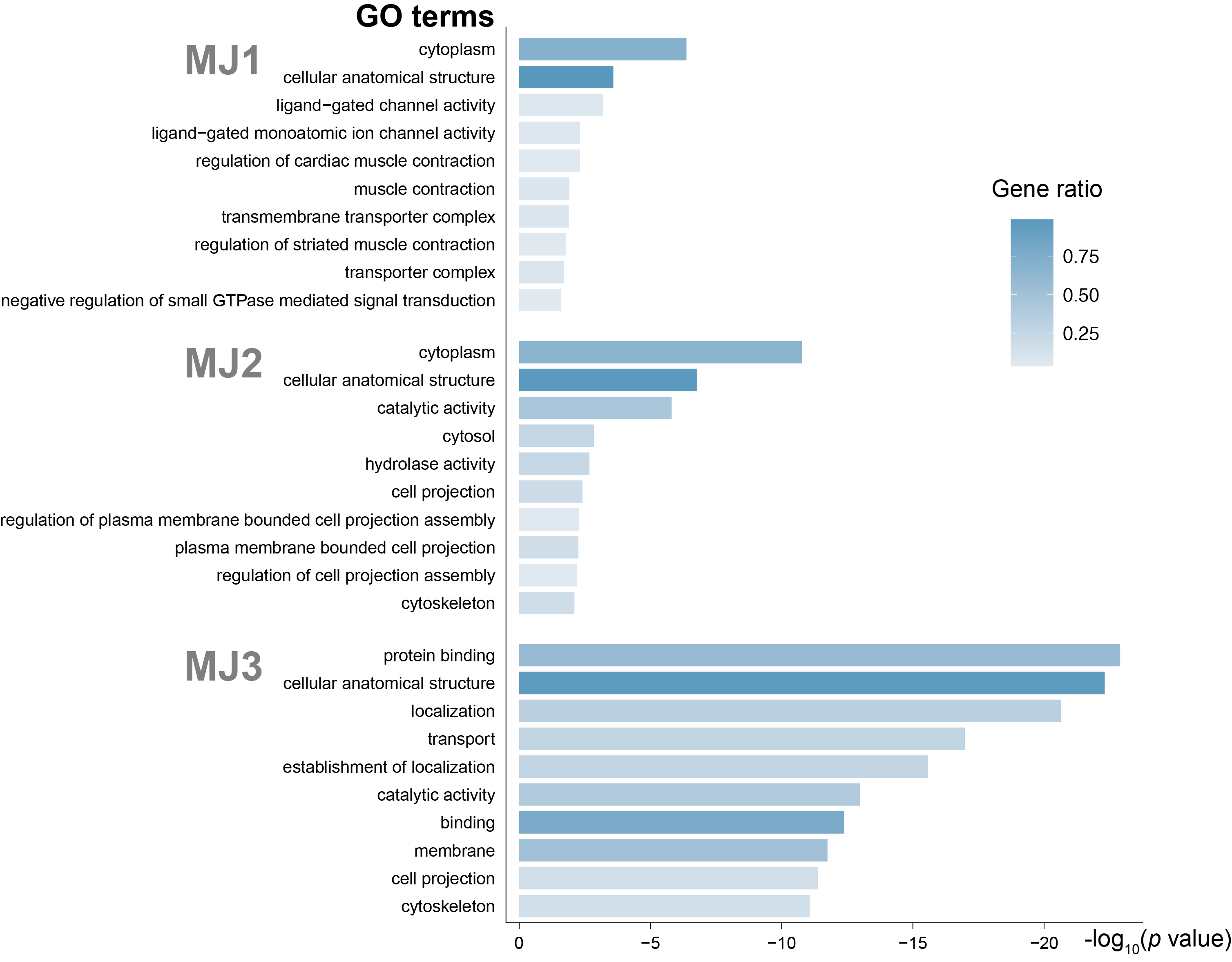


**Supplementary Figure 19 | GO enrichment results of the gene set associated with Loss of Function variants.**

**Reference** **for supplementary information**

1 Cao, P. *et al.* Genome-wide signatures of mammalian skin covering evolution. *Science China Life Sciences*, 1-16 (2021).

2 Lopez, J. V., Cevario, S. & O'Brien, S. J. Complete nucleotide sequences of the domestic cat (*Felis catus*) mitochondrial genome and a transposed mtDNA tandem repeat (Numt) in the nuclear genome. *Genomics* **33**, 229-246 (1996).

3 Kim, K. S., Lee, S. E., Jeong, H. W. & Ha, J. H. The complete nucleotide sequence of the domestic dog (*Canis familiaris*) mitochondrial genome. *Molecular phylogenetics and evolution* **10**, 210-220 (1998).

4 Hassanin, A., Hugot, J.-P. & van Vuuren, B. J. Comparison of mitochondrial genome sequences of pangolins (Mammalia, Pholidota). *Comptes rendus biologies* **338**, 260-265 (2015).

5 Wang, Q. *et al.* Whole-genome resequencing of Chinese pangolins reveals a population structure and provides insights into their conservation. *Communications Biology* **5**, 821 (2022).

6 Gaubert, P. *et al.* The complete phylogeny of pangolins: scaling up resources for the molecular tracing of the most trafficked mammals on earth. *Journal of Heredity* **109**, 347-359 (2018).

7 Hu, J.-Y. *et al.* Genomic consequences of population decline in critically endangered pangolins and their demographic histories. *National Science Review* **7**, 798-814, doi:10.1093/nsr/nwaa031 (2020).

8 Choo, S. W. *et al.* Pangolin genomes and the evolution of mammalian scales and immunity. *Genome research* **26**, 1312-1322 (2016).

9 vonHoldt, B. M. *et al.* Whole-genome sequence analysis shows that two endemic species of North American wolf are admixtures of the coyote and gray wolf. *Science Advances* **2**, e1501714 (2016).

10 Ming, L. *et al.* Whole-genome sequencing of 128 camels across Asia reveals origin and migration of domestic Bactrian camels. *Communications Biology* **3**, 1 (2020).

11 Guang, X. *et al.* Chromosome-scale genomes provide new insights into subspecies divergence and evolutionary characteristics of the giant panda. *Science bulletin* **66**, 2002-2013 (2021).

12 Hansen, C. C. R. *et al.* The muskox lost a substantial part of its genetic diversity on its long road to Greenland. *Current Biology* **28**, 4022-4028. e4025 (2018).

13 Pfeifer, S. P. The demographic and adaptive history of the African green monkey. *Molecular biology and evolution* **34**, 1055-1065 (2017).

14 Carneiro, M. *et al.* The genomic architecture of population divergence between subspecies of the European rabbit. *PLoS Genetics* **10**, e1003519 (2014).

15 Armstrong, E. E. *et al.* Recent evolutionary history of tigers highlights contrasting roles of genetic drift and selection. *Molecular biology and evolution* **38**, 2366-2379 (2021).

16 Zhang, L. *et al.* Chromosome‐scale genomes reveal genomic consequences of inbreeding in the South China tiger: A comparative study with the Amur tiger. *Molecular Ecology Resources* **23**, 330-347, doi:<https://doi.org/10.1111/1755-0998.13669> (2023).

17 Pečnerová, P. *et al.* High genetic diversity and low differentiation reflect the ecological versatility of the African leopard. *Current Biology* **31**, 1862-1871. e1865 (2021).

18 Consortium, G. P. A map of human genome variation from population scale sequencing. *Nature* **467**, 1061 (2010).

19 Reeves, I. M. *et al.* Population genomic structure of killer whales (Orcinus orca) in Australian and New Zealand waters. *Marine Mammal Science* **38**, 151-174 (2022).

20 Fünfstück, T. *et al.* The genetic population structure of wild western lowland gorillas (*Gorilla gorilla gorilla*) living in continuous rain forest. *American Journal of Primatology* **76**, 868-878 (2014).

21 Liu, G. *et al.* Population genomics reveals moderate genetic differentiation between populations of endangered Forest Musk Deer located in Shaanxi and Sichuan. *BMC genomics* **23**, 1-11 (2022).

22 Khan, A. *et al.* Genomic evidence for inbreeding depression and purging of deleterious genetic variation in Indian tigers. *Proceedings of the National Academy of Sciences* **118**, e2023018118, doi:<https://doi.org/10.1073/pnas.2023018118> (2021).

23 Gu, T.-T. *et al.* Genomic analysis reveals a cryptic pangolin species. *Proceedings of the National Academy of Sciences* **120**, e2304096120, doi:doi:10.1073/pnas.2304096120 (2023).

24 Heighton, S. P. *et al.* Pangolin Genomes Offer Key Insights and Resources for the World’s Most Trafficked Wild Mammals. *Molecular Biology and Evolution* **40**, doi:10.1093/molbev/msad190 (2023).

25 Nash, H. C. *et al.* Conservation genomics reveals possible illegal trade routes and admixture across pangolin lineages in Southeast Asia. *Conservation Genetics* **19**, 1083-1095 (2018).

26 Wang, P. *et al.* Genomic consequences of long-term population decline in brown eared pheasant. *Molecular Biology and Evolution* **38**, 263-273 (2021).

27 Yang, S. *et al.* Genomic investigation of the Chinese alligator reveals wild‐extinct genetic diversity and genomic consequences of their continuous decline. *Molecular Ecology Resources* **23**, 294-311 (2023).

28 Zhou, X. *et al.* Baiji genomes reveal low genetic variability and new insights into secondary aquatic adaptations. *Nature communications* **4**, 2708 (2013).

29 Zhou, X. *et al.* Population genomics reveals low genetic diversity and adaptation to hypoxia in snub-nosed monkeys. *Molecular Biology and Evolution* **33**, 2670-2681 (2016).

30 Xue, Y. *et al.* Mountain gorilla genomes reveal the impact of long-term population decline and inbreeding. *Science* **348**, 242-245 (2015).

31 Robinson, J. A. *et al.* Genomic flatlining in the endangered island fox. *Current Biology* **26**, 1183-1189 (2016).

32 Corbett-Detig, R. B., Hartl, D. L. & Sackton, T. B. Natural selection constrains neutral diversity across a wide range of species. *PLoS biology* **13**, e1002112 (2015).
